# Hepatic ribosomal protein S6 (Rps6) insufficiency results in failed bile duct development and loss of hepatocyte viability; a ribosomopathy-like phenotype that is partially p53-dependent

**DOI:** 10.1371/journal.pgen.1010595

**Published:** 2023-01-19

**Authors:** Sarah A. Comerford, Elizabeth A. Hinnant, Yidong Chen, Robert E. Hammer

**Affiliations:** 1 Department of Molecular Genetics, University of Texas Southwestern Medical Center, Dallas, Texas, United States of America; 2 Department of Biochemistry, University of Texas Southwestern Medical Center, Dallas, Texas, United States of America; 3 Department of Population Health Sciences, University of Texas Health San Antonio, San Antonio, Texas, United States of America; 4 Greehey Children’s Cancer Research Institute, University of Texas Health San Antonio, San Antonio, Texas. United States of America; Seattle Children’s Research Institute, UNITED STATES

## Abstract

Defective ribosome biogenesis (RiBi) underlies a group of clinically diverse human diseases collectively known as the ribosomopathies, core manifestations of which include cytopenias and developmental abnormalities that are believed to stem primarily from an inability to synthesize adequate numbers of ribosomes and concomitant activation of p53. The importance of a correctly functioning RiBi machinery for maintaining tissue homeostasis is illustrated by the observation that, despite having a paucity of certain cell types in early life, ribosomopathy patients have an increased risk for developing cancer later in life. This suggests that hypoproliferative states trigger adaptive responses that can, over time, become maladaptive and inadvertently drive unchecked hyperproliferation and predispose to cancer. Here we describe an experimentally induced ribosomopathy in the mouse and show that a normal level of hepatic ribosomal protein S6 (Rps6) is required for proper bile duct development and preservation of hepatocyte viability and that its insufficiency later promotes overgrowth and predisposes to liver cancer which is accelerated in the absence of the tumor-suppressor PTEN. We also show that the overexpression of c-Myc in the liver ameliorates, while expression of a mutant hyperstable form of p53 partially recapitulates specific aspects of the hepatopathies induced by Rps6 deletion. Surprisingly, co-deletion of p53 in the Rps6-deficient background fails to restore biliary development or significantly improve hepatic function. This study not only reveals a previously unappreciated dependence of the developing liver on adequate levels of Rps6 and exquisitely controlled p53 signaling, but suggests that the increased cancer risk in ribosomopathy patients may, in part, stem from an inability to preserve normal tissue homeostasis in the face of chronic injury and regeneration.

## Author summary

Ribosomopathies are a group of human diseases caused by mutations in genes that are required for ribosome biogenesis (RiBi), the process by which ribosomes, the molecular machines that synthesize all of the proteins in our bodies, are made. Here we show that mice that lack sufficient levels of one such gene, ribosomal protein S6 (*Rps6*) as the liver develops, fail to develop primary bile ducts resulting in disease reminiscent of North American Indian childhood cirrhosis (NAIC), a liver-specific ribosomopathy caused by mutations in a different RiBi gene, *UTP4*. Hepatocytes in Rps6-deficient livers also die resulting in small, abnormal livers that are forced to regenerate and predisposed to develop cancer. To determine the contribution of p53, a primary effector of the anti-proliferative response that is often triggered by dysfunctional RiBi to the liver disease caused by Rps6-deficiency, we find that forced activation of p53 inhibits bile duct development, but that the disease in Rps6-deficient livers is not solely p53-dependent. This study reveals a previously unappreciated vulnerability of the liver to defective RiBi or inappropriate activation of p53 and implicates both as possible drivers of a subset of congenital biliary or hepatic insufficiency syndromes for which there is currently no known etiology.

## Introduction

The production of mature ribosomes, the protein synthesizing factories of the cell, is an essential and highly conserved process that occurs in the nucleolus of every cell in the body. As a complex, highly regulated process, ribosome biogenesis (RiBi) requires >200 factors to coordinate the synthesis and processing of ribosomal RNA (rRNA) with the production and assembly of ribosomal proteins (rps) into the large (60S) and small (40S) ribosomal subunits to ensure a constant supply of ribosomes for maintaining proteostasis [1,2]. While the importance of this process for sustaining growth and maintaining tissue homeostasis is exemplified by the Minutes, a series of *Drosophila melanogaster* mutants that exhibit developmental delay and shortened bristles due to mutations in rp genes [3], whole genome sequencing has identified a diverse set of congenital human diseases collectively known as the ribosomopathies that are due to haploinsufficient mutations in ribosomal proteins or other essential RiBi factors that have the potential to disrupt rRNA synthesis, rRNA processing or ribosomal subunit assembly or maturation [4–6]. Ribosomopathies are characterized by developmental abnormalities and cytopenias that are not only believed to reflect activation of the ribosomal or nucleolar stress response, a surveillance mechanism that is triggered to prevent progression through the cell cycle when ribosomes are in short supply [7], but also the collateral loss of extraribosomal functions that have been attributed to many RiBi genes [8,9]. While bone marrow failure, skeletal and craniofacial defects are common across many of the ribosomopathies [6,10,11], developmental defects also occur in a variety of other organs including the pancreas [12], spleen [13] and testes [14] illustrating the breadth of the impact that RiBi dysfunction can have on organogenesis and tissue homeostasis.

Although stabilization of the tumor suppressor p53 has been identified as a key effector of the nucleolar stress response [7,15,16], studies in experimental systems indicate that p53-dependent and -independent mechanisms participate in driving cells into arrest or senescence, or triggering apoptosis when RiBi is compromised [17–23]. Given the large number of genes involved in orchestrating RiBi, the complexity of p53 signaling [24] and the broad range of organs that are affected in the ribosomopathies, major challenges in the field are to determine why specific cell types are preferentially impacted by mutations in a particular RiBi gene and to understand the extent to which acute and long-term responses that are triggered to mitigate these cellular deficiencies influence disease progression and outcomes. This is important in light of the fact that an undesirable long-term consequence of some ribosomopathies is an elevated risk of developing cancer later in life [25–27]. While the molecular and cellular basis for the increased cancer risk is poorly understood, the paradox in which hyperproliferative disease follows a hypoproliferative state is consistent with observations that haploinsufficient ribosomal protein mutations in *D*. *melanogaster* and *Danio rerio* initially impair growth, but later result in overgrowth phenotypes [28,29] or cancer [30–32], suggesting that the persistent engagement of compensatory mechanisms that are triggered to re-balance cellular homeostasis may inadvertently promote hyperplastic growth and/or tumor development.

Despite being one of the most quiescent organs in the body, the liver is unmatched in its ability to respond to unscheduled gains or losses in mass [33], making it ideal for studying the ribosomal stress-activated response when challenged with differing protein synthesis demands. Previous studies have shown that acute ablation of ribosomal protein S6 (Rps6/eS6) prior to 70% hepatectomy in adult mouse liver blocks regeneration [34], demonstrating the exquisite dependence of highly proliferative hepatocytes with a heightened demand for protein synthesis on a fully functioning RiBi machinery. However, given that many ribosomopathy patients display congenital defects that reflect the impact of RiBi dysfunction during development as protein synthesis demands fluctuate, we wanted to determine the extent to which liver development and homeostasis depends on adequate levels of Rps6. We did this by conditionally deleting Rps6 at distinct times and in specific cell types; namely hepatoblasts of the embryonic liver that give rise to mature hepatocyes and biliary cells that form the bile ducts, and in post-mitotic hepatocytes of the adult liver. Our results show that both immature and adult hepatocytes depend on Rps6 for survival and that the developmental timing of deletion profoundly impacts the severity of liver disease. Moreover, we find that loss of Rps6 from hepatoblasts as they differentiate into biliary cells inhibits bile duct development resulting in cholestasis and a near-fatal hepatic failure that stunts neonatal growth and forces livers to regenerate via the proliferation of Rps6-expressing cells, a subset of which demonstrate activation of mTOR. Chronic hepatic Rps6 insufficiency also predisposed to hepatomegaly and spontaneous tumor development that was accelerated by loss of the tumor suppressor Pten. Using additional strains of mice that either overexpress or lack genes that are known to influence RiBi, we found that modest overexpression of c-Myc in the liver is sufficient to rescue the hepatocyte death caused by loss of Rps6, and in doing so, alters the immediate-early, but not long-term hepatic response to Rps6-insufficiency. Moreover, using gain- and loss-of-function genetic approaches to investigate the role of p53 in the phenotypes resulting from loss of hepatic Rps6, we find that hepatoblast-specific stabilization of an Mdm2-resistant p53 mutant only partially mimics the liver disease in S6-deficient livers, while the loss of p53 fails to improve disease induced by Rps6 deficiency indicating that p53 is not the sole pathogenic driver in this model. These studies not only reveal a previously unappreciated dependence of biliary development and hepatocyte survival on adequate levels of Rps6, but also implicate unscheduled activation of p53 in a subset of idiopathic cholangiopathies. Our data also suggest that the increased cancer risk in ribosomopathies may, in part, reflect unstable tissue environments resulting from the persistent engagement of mechanisms that are triggered to limit tissue damage while also promoting compensatory proliferation in an attempt to re-establish cellular and functional homeostasis.

## Results

### Perinatal deletion of hepatic Rps6 stunts growth and induces severe neonatal liver hypoplasia

**Fig 1:**
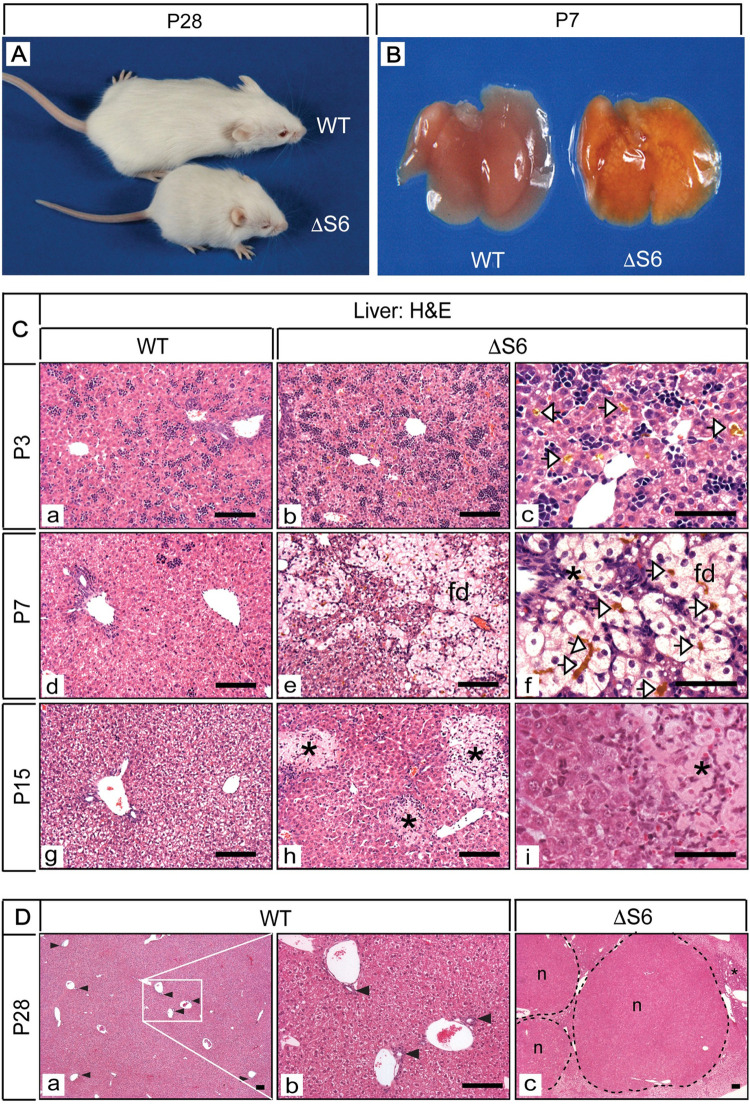
Perinatal deletion of hepatic Rps6 retards growth and results in cholestatic liver disease. (A) A WT and runted ΔS6 mouse at postnatal day 28 showing that hepatic *Rps6*- deficiency retards neonatal growth. (B) Gross appearance of livers from a WT and a ΔS6 mouse at post-natal day 7 (P7). Severe yellowing of the ΔS6 liver is indicative of cholestatic disease. (C) Photomicrographs of H&E stained sections of liver from WT mice at P3, P7 and P15 (a, d and g) and age-matched ΔS6 mice (b-i). Canalicular accumulation of bile (yellow deposits, open arrowheads) is evident in ΔS6 livers at P3 and P7 and feathery degeneration (fd) of hepatocytes is evident at P7. At P15, bile infarcts (_*_) resulting from bile leakage due to canalicular or hepatocyte membrane rupture can be seen throughout the parenchyma of ΔS6 livers (Original magnifications, a, b, d, e, g, h (x 125; 50μ scale bars); c, f and i (x 375; 25μ scale bars). (D) Photomicrographs of H&E stained sections of liver from a WT mouse (a and b) and a ΔS6 littermate (c) at 4 weeks of age. In contrast to the WT liver (a and b) which shows an abundance of bile ducts (arrowheads), ΔS6 livers appear to either lack or have a paucity of bile ducts (c) while nodules (n) and areas of biliary hyperplasia (*) are prominent indicating that Rps6 insufficiency has severely disrupted liver architecture. (Original magnifications, a and c (x 32.5); b (x 125)). Scale bars; all 50μ.

To delete hepatic Rps6 prior to birth, mice harboring a conditional *Rps6*^*lox/lox*^ allele [34] were bred to mice expressing Albumin-Cre (*Alb-Cre*) which specifies gene targeting in hepatoblasts of the embryonic liver from ~E15 onwards [35,36]. Progeny with all of the genotypes from matings of *Rps6*^*lox/wt*^ mice to *Rps6*^*lox/wt*^:*Alb-Cre* mice were born at the expected Mendelian ratio indicating that targeted deletion of hepatic Rps6 prior to birth in *Rp6*^*lox/lox*^:*Alb-Cre* mice (herein referred to as ΔS6 mice) did not cause embryonic lethality. However, monitoring of post-natal body weight showed that ΔS6 mice were significantly smaller than their wild-type (WT, Cre-) and S6^lox/wt;^*Alb-Cre* littermates between ~2–6 weeks of age (Figs 1A, S1A, and S1B) and had livers that were disproportionately small in relation to body size (S1C and S1D Fig). Analysis of the targeted *Rps6*^*lox/lox*^ allele (ΔS6^*del*^) in liver showed that recombination was low at birth, but increased to maximal levels of ~50–60% by ~2 weeks of age, coinciding with the onset of growth retardation and liver hypoplasia (S2A and S2B Fig). Northern blotting (S2C and S2D Fig) and immunohistochemistry of ΔS6 livers at post-natal day 15 (P15) (S2E Fig) confirmed that Rps6 deletion was incomplete, regional and varied between mice with livers expressing 30–50% of the normal amount of *Rps6* mRNA. Despite being runted as neonates, ΔS6 body weight gradually increased suggesting that hepatic Rps6 deficiency was delaying, rather than permanently stunting growth (S1A and S1B Fig). Given the hypoplastic nature of ΔS6 livers and the liver’s ability to re-establish functional tissue mass through regeneration, we asked if the catch-up in body weight reflected improved liver function by performing liver function tests (LFTs) on plasma collected from WT and ΔS6 mice at 4–5 weeks of age when ΔS6 mice were at their most runted, and again at 8–9 weeks of age as they neared normal weight. Analysis showed that while biliary function (Alk-Phos and T-Bil) had improved within this period, markers of hepatocellular damage (ALT and AST) remained elevated (S3 Fig) indicating that livers of ΔS6 mice remained functionally compromised well into adulthood.

### Hepatic Rps6 deficiency inhibits bile duct development and induces cholestatic liver injury provoking regeneration

**Fig 2:**
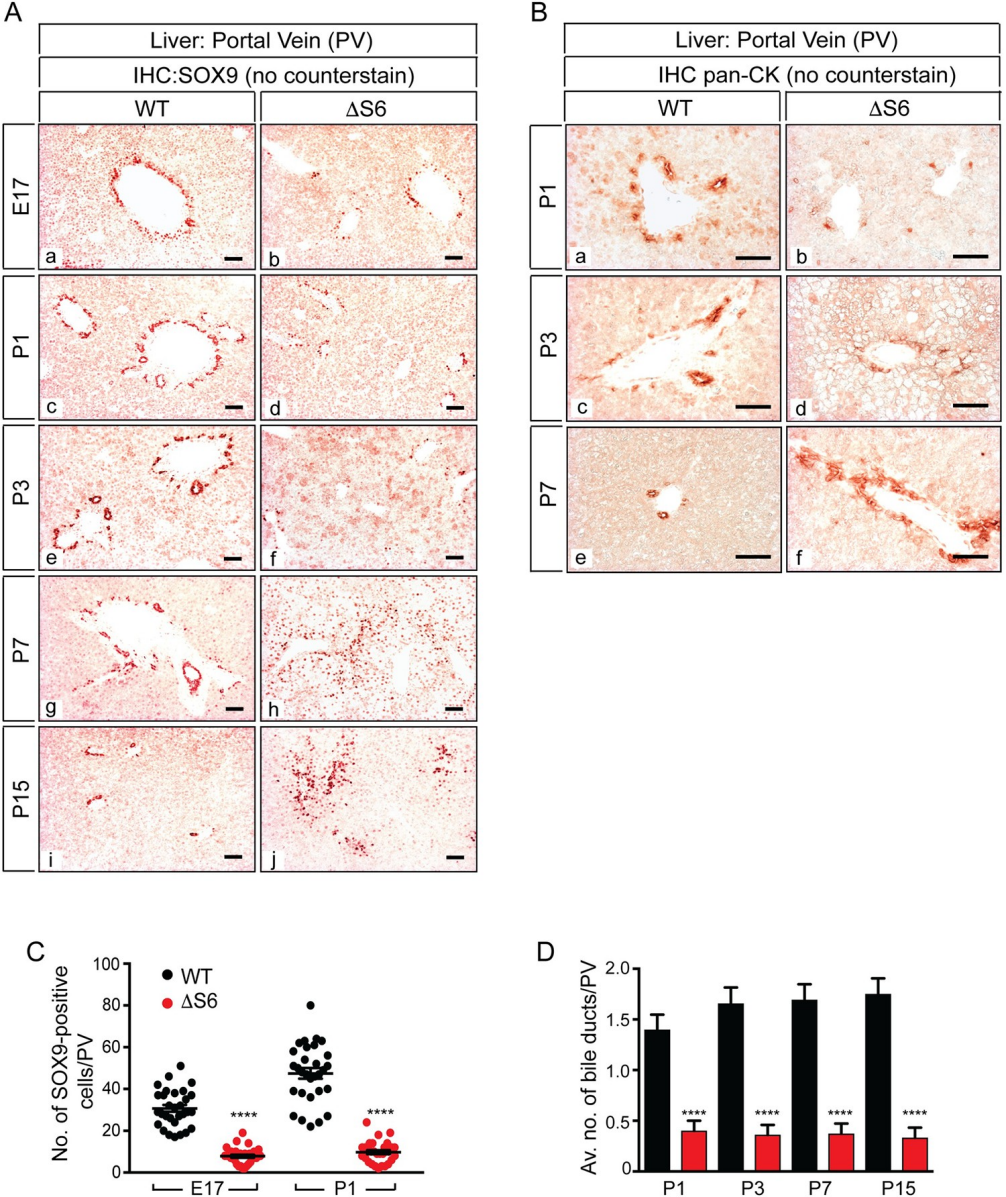
Hepatic *Rps6*-deficiency inhibits bile duct development. (A) Photomicrographs of SOX9 IHC of WT (a, c, e, g, i) and ΔS6 (b, d, f, h, j) livers from E17 to P15 (Original magnifications, all x 125; 50μ scale bars) (AEC chromogen (red/orange), no counterstain). Note the gradual disappearance of SOX9-positive cells from the ductal plate in ΔS6 livers between E17-P3 and the abnormal position and expansion of SOX9-positive cells throughout the parenchyma from P7 onwards. (B) Photomicrographs of pan-CK IHC of WT (a, c, e) and ΔS6 (b, d, f) livers from P1 to P7 showing that the number of pan-CK-positive biliary cells in ΔS6 livers is also reduced at P1 and P3 (b and d) while their expansion at P7 (f) mirrors the increase in the number of SOX9-positive cells (A (h)) signifying emergence of a nascent ductular reaction. (Original magnifications, all x 250; 50μ scale bars) (AEC chromagen (red/orange), no counterstain). (C) Graph depicting quantitative analysis of the number of SOX9-positive cells around portal veins (PVs) in WT and ΔS6 livers at E17 and P1. ΔS6 livers have 20–25% of the normal number of SOX9-positive cells (mean values 7.8 vs 30.7 at E17; *P* < .0001 and 9.8 vs 47.5 at P1; *P* < .0001); 2-tailed unpaired Student’s *t*-test. (D) Graph showing average number of bile ducts per portal vein (PV) (bds/PV) in WT and ΔS6 livers at P1, P3, P7 and P15. While WT livers have an average of 1–2 fully formed bds/PV, ΔS6 livers have an average of < 0.5. Data are mean ± SEM; **** *P* < .0001; 2-tailed unpaired Student’s *t*-test.

The perinatal/neonatal period constitutes the most dynamic period for the liver during which bile duct development is nearing completion at a time when hepatic mass is increasing at its fastest rate while also responding to the dramatic metabolic adaptations that occur at birth and at weaning [37,38]. Given that hepatoblast-specific ablation of Rps6 has the potential to impact hepatocytes and biliary cells, we analyzed ΔS6 livers from the perinatal period to ~10 weeks of age to determine how loss of Rps6 impacted the liver at this crucial time as it transitioned from an immature to fully mature functional organ. Gross inspection of ΔS6 livers revealed marked yellowing by P7 (Fig 1B) consistent with jaundice. Histological evaluation of H&E stained sections of ΔS6 livers showed evidence of canalicular bile accumulation at P3 (panel c of Fig 1C) and cholate stasis at P7 as determined by the presence of widespread feathery degeneration of hepatocytes, a form of non-apoptotic inflammatory-induced cholestatic death characterized by hepatocyte ballooning and flocculent cytoplasmic inclusions, akin to necroptosis [39] (panels e and f of Fig 1C). Foci of dead hepatocytes or bile infarcts were also evident at P15 (panels h and i of Fig 1C), consistent with toxic bile acid-mediated degeneration leading to confluent hepatocyte necrosis. Examination of H&E stained liver sections from young adult mice also indicated that liver architecture was dramatically altered in ΔS6 livers and that they appeared to have fewer bile ducts than their WT counterparts (Fig 1D). To determine if Rps6-insufficiency was interfering with bile duct development or causing the loss of pre-existing bile ducts, we performed IHC of WT and ΔS6 livers from E17, shortly after establishment of the ductal plate, to P15 when biliary development and morphogenesis is complete [40]. Using an antibody specific for Sox9, a transcription factor that marks immature biliary cells and mature bile ducts but not mature hepatocytes, we found that ΔS6 livers had fewer Sox9-positive cells surrounding the portal vein than WT livers by E17 (panels a and b of Fig 2A). Moreover, of the few Sox9-positive cells that were visible, none became incorporated into structures resembling bile ducts as biliary development progressed (panels c-j of Fig 2A), a finding that was confirmed by performing IHC with a pan-cytokeratin (CK) antibody that preferentially recognizes biliary-type CKs (CK7/19) (Fig 2B) Quantitative analysis confirmed that ΔS6 livers had only 25% and 21% of the normal number of Sox9-positive cells at E17 and P1 respectively (Fig 2C) and fewer still at P3 (panel f of Fig 2A), and had either 1 or no bile ducts per portal vein (bds/PV) (mean, < 0.5 bds/PV) in contrast to livers of WT mice which had 1–3 (mean, 1.4 bds/PV) (Fig 2D). Finally, while Sox9- and pan-CK-positive cells that had not been incorporated into bile ducts were rare or absent in WT livers at P7 or P15 (panels g and i of Fig 2A and panel e of Fig 2B), Sox9-expressing cells had begun to proliferate around and radiate out from portal veins in ΔS6 livers (panels h and j of Fig 2A and panel f of Fig 2B) in a pattern that suggested emergence of a nascent ductular reaction (dr) (panels h and j of Fig 2A), which is a hallmark of hepatic progenitor cell (HPC) activation in response to extreme hepatic injury [41].

**Fig 3:**
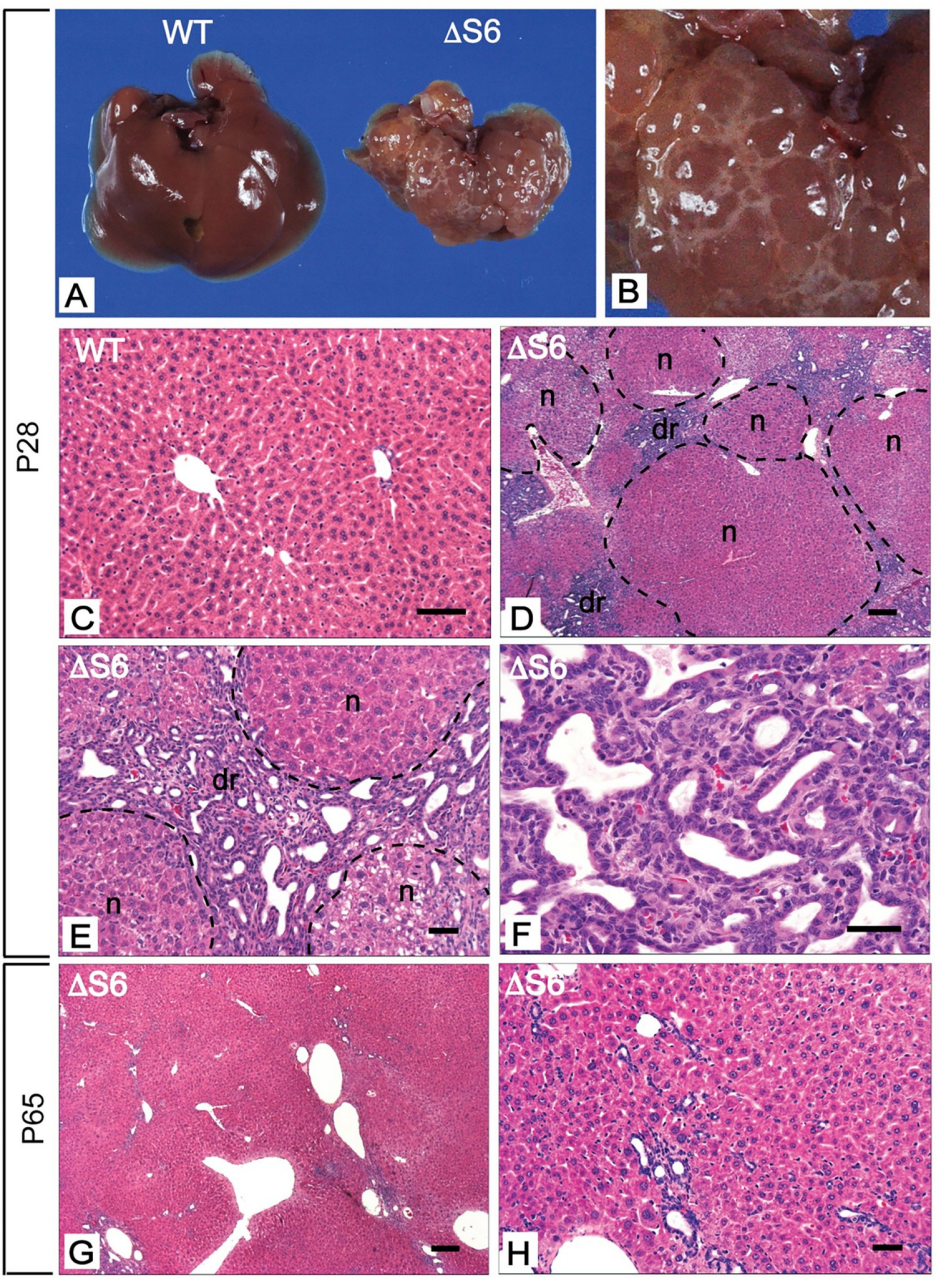
Perinatal deletion of hepatic Rps6 results in hypoplastic livers and triggers regeneration. (A) Gross appearance of livers from a WT and ΔS6 mouse at postnatal day 28 (P28). The ΔS6 liver is smaller than the WT liver, is discolored and has an uneven mottled appearance. (B) Close-up image of ΔS6 liver in a) highlighting “cystic-like” nodules on the surface of the liver. (C-H) Photomicrographs of H&E stained sections of liver from a WT mouse (C) and ΔS6 mice at P28 (D-F) and P65 (G and H). At P28, ΔS6 livers display an abundance of regenerative nodules (n) and a prominent ductular reaction (dr) signifying dynamic regeneration in response to severe injury. By P65, the absence of regenerative nodules indicates that the regenerative response has largely dissipated. However, hepatocyte morphology is heterogeneous and remnants of the dr persist as seen by the presence of irregular luminal structures in the vicinity of portal veins (g, h). (Original magnifications, C (x 112); D, G (x 32); E, H (x 125) and F (x 250)). Scale bars correspond to 50μ for C, E, F and H and 200μ for D and G.

Examination of ΔS6 livers as mice reached adulthood showed that despite the gradual normalization of body weight, livers remained small and were discolored and uneven with a “cystic-like” appearance (Fig 3A and 3B). Histological evaluation of livers at P28 revealed that in contrast to the typical, well ordered lobular architecture of WT liver (Fig 3C), ΔS6 livers contained an abundance of regenerative nodules comprised of cells that resembled immature hepatocytes interspersed by cords of small oval-shaped cells that were attempting to organize into ducts consistent with induction of a full-blown dr (Fig 3D, 3E and 3F) which can facilitate secondary bile duct development [42]. IHC profiling of ΔS6 livers using a panel of antibodies directed against proliferating cell nuclear antigen (PCNA) and a variety of liver cell markers that are selectively expressed in mature and immature liver cells showed that nodules contained highly proliferative immature hepatocytes (S4F Fig) that expressed abundant AFP (S4H Fig) and HNF4α (S4J Fig). This was in contrast to the majority of ductular cells which expressed a variety of biliary (HNF1β, SOX9, (pan)-cytokeratin(CK)) (S4L, S4N and S4P Fig) and HPC (TROP2 and EPCAM) (S4R and S4T Fig) markers, but not HNF4α or AFP. IHC for β-catenin, whose level of expression and sub-cellular localization is an indicator of wnt signaling status, showed that although HPCs and immature nodular hepatocytes both expressed β-catenin, expression was significantly enriched in the ductular cell population compared to nodular hepatocytes (S4V Fig). Moreover, retention of β-catenin at the membrane of nodular hepatocytes, rather than cytoplasmic or nuclear localization (S4V Fig) and the absence of glutamine synthetase expression (GLUL), a classical wnt target within nodules (S4X Fig), indicated that hyperactive wnt signaling was not driving nodular growth in ΔS6 livers. Evaluation of ΔS6 livers at ~8–10 weeks of age showed a marked reduction in the dr and restoration of normal liver architecture (Fig 3G). However morphological and nuclear heterogeneity within hepatocytes persisted indicating that ΔS6 livers remained compromised even after regenerating (Fig 3H). Taken together, these results show that hepatoblast-specific deletion of Rps6 profoundly disrupts perinatal liver development by compromising hepatocyte survival and interfering with bile duct development by limiting the pool of Sox9-expressing biliary precursors within the ductal plate, all of which leads to a ribosomopathy-like phenotype characterized by hepatic hypoplasia, sub-lethal hepatic failure and regeneration.

### Hepatic dysfunction in ΔS6 livers reflects loss of Rps6 in hepatocytes and biliary cells

In addition to being a constituent of the 40S ribosomal subunit, Rps6 is unique among the rps in that it is also a phospho-protein whose phosphorylation is regulated by the rapamycin-sensitive branch of the mTOR/S6K signaling (mTORC1) pathway, a conserved nutrient and energy-sensing pathway that positively regulates metabolism, growth, proliferation and survival [43]. Given that Rps6 phosphorylation has been shown to be required for a diverse array of physiological and pathological functions [44], we were interested in establishing the normal distribution of Rps6 in WT liver and assessing its phosphorylation status in any Rps6-expressing cell populations to determine if the loss of hepatocyte viability or the bile duct defect simply reflected cell-autonomous loss of Rps6 expression in each cell-type or whether they also involved the loss of additional phosphorylation-dependent functions. We therefore performed IHC with an antibody that recognizes Rps6 irrespective of its phosphorylation status (total Rps6) and another that recognizes Rps6 only when phosphorylated on the mTOR-dependent sites Ser^235/236^ (phospho-Rps6). IHC with the total and phospho-specific Rps6 antibodies showed that normal hepatocytes expressed cytosolic Rps6 protein in a decreasing periportal (pp)-to-central vein (cv) gradient (panel a of S5A Fig), with the phospho-Rps6 antibody predominantly staining periportal hepatocytes consistent with the highest degree of phosphorylation being in periportal zone 1 (panel b of S5A Fig). This contrasted with bile ducts which, despite expressing abundant Rps6 (panel c of S5A Fig), failed to demonstrate any phospho-specific Rps6 staining (panel d of S5A Fig). This finding indicates that although Rps6 is expressed in both functional compartments of the liver, both cell types differ with respect to their Rps6 phosphorylation/mTOR activation status at least on Ser^235/6^. Thus, while loss of hepatocyte viability and the cholangiopathy in ΔS6 livers likely reflects the cell-autonomous impact of Rps6 loss on each cell type, it is possible that the loss of phosphorylation-specific functions of Rps6 also contribute to the hepatocyte, but not the biliary defect in ΔS6 livers.

### Regeneration in ΔS6 livers is mediated via the proliferation of 2 different Rps6-expressing cell types: HPCs and mTOR-activated immature hepatocytes

In light of incomplete Alb-Cre-mediated deletion of Rps6 during the perinatal period (S2 Fig), we performed IHC with the same two Rps6-specific antibodies to determine if ΔS6 livers were regenerating via the proliferation of residual Rps6-expressing cells or other cell types, and if so, whether Rps6 was phosphorylated (and thus mTOR activated) in such cells. IHC with the total-Rps6 antibody showed that regenerating ΔS6 livers contained two different Rps6-expressing cell populations; namely Sox9-positive dr/HPC cells and AFP-positive nodular hepatocytes (panel e of S5A Fig). However, only nodular hepatocytes demonstrated phospho-Rps6 immunoreactivity indicating that both cell types differed with respect to their Rps6^235/6^ phosphorylation status (panel f of S5A Fig). Immunoblotting confirmed that another mTOR target, eIF4E binding protein 1 (4E-BP1), was also hyper-phosphorylated in ΔS6 livers (S5B Fig), while Akt, an effector of PI3K signaling that lies upstream of mTOR failed to show any significant increase in phosphorylation above basal levels, indicating that mTOR had been activated in regenerating ΔS6 livers and that its activation was independent of PI3K/Akt. To definitively show that Rps6 phosphorylation in nodular hepatocytes reflected mTOR activation, we treated a cadre of ΔS6 mice with the mTOR inhibitor rapamycin and assessed total and phospho-Rps6 expression by IHC (S5C Fig) and immunoblotting (S5D Fig). As expected, livers of vehicle-treated ΔS6 mice retained strong staining of nodular hepatocytes and dr cells with the total Rps6 antibody reflecting Rps6 expression in both cell types irrespective of its phosphorylation status (panels a, c, and e of S5C Fig). However, rapamycin treatment of ΔS6 mice abolished both total and phospho-Rps6 staining in all but a few solitary parenchymal cells, confirming that all of the Rps6 in nodular hepatocytes was phosphorylated in an mTORC1-dependent manner (panels b, d, and f of S5C Fig). This was in direct contrast to the Rps6-expressing ductular cells whose staining was unaffected by rapamycin, consistent with the absence of a Ser^235/236^-phosphorylated form of Rps6 in these cells (panel fof S5A Fig). Thus, regeneration in ΔS6 livers is mediated by 2 different Rps6-expressing cell types; HPCs without mTOR activation and mTOR-activated, AFP-expressing immature hepatocytes suggesting that Rps6-expression confers a survival and/or proliferative advantage in the context of hepatic Rps6 deficiency.

### Hepatic Rps6-deficiency activates p53, disrupts rRNA processing and activates a transcriptional program indicative of de-differentiation/regeneration, cell cycle arrest/senescence and inflammation

In light of previous reports documenting p53 stabilization and activation of the p53-dependent checkpoint in other mouse models of Rps6 deficiency [34,45,46], we analyzed the p53 status of ΔS6 livers. Immunoblotting of liver lysates from WT and ΔS6 livers with a p53-specific antibody showed that it recognized 2 proteins; a faster migrating, non-specific protein that was present in all samples and a slower migrating protein representing *bona fide* p53 that was abundant in ΔS6 livers and in A431 cells that express high levels of mutant p53, but which was absent from WT livers and p53-deficient Saos2 cells confirming that p53 had been stabilized in ΔS6 livers (S6A Fig). To establish the spatio-temporal pattern of p53 stabilization following Alb-Cre-mediated deletion of Rps6, we performed IHC with the same p53-specific antibody on sections of WT and ΔS6 livers at E17 and P7 after validating it on livers that express SV40 large T-Antigen (TAg) [47], an oncoprotein that binds and stabilizes p53 in the nucleus (panel a of S6B Fig). While the paucity of biliary cells in ΔS6 livers precluded us from determining if p53 had been stabilized in cholangiocytes, IHC showed abundant nuclear p53 in a subset of hepatoblasts in ΔS6 livers at E17 (panels d and f of S6B Fig) and in the majority of hepatocytes undergoing feathery degeneration and in neighboring hepatocytes at P7 (panels e and g of S6B Fig) indicating that p53 had undergone rapid stabilization following Alb-Cre-mediated deletion of Rps6.

As defective rRNA processing is a hallmark of ribosomal stress we also assessed the relative abundance of rRNA intermediates in RNA isolated from WT and ΔS6 liver by Northern Blotting using rRNA-specific radiolabeled probes homologous to regions within intervening sequence 1 and 2 (ITS1 and ITS2) of the 47S rRNA. Hybridization with the ITS1-specific probe showed accumulation of 30S rRNA and a corresponding decrease in the abundance of 21S rRNA in ΔS6 livers (S6C Fig) while hybridization with the ITS2-specific probe showed a reduced abundance of 17S rRNA (S6C Fig) confirming that loss of Rps6 had disrupted rRNA processing. Re-probing of both blots with an 18S rRNA-specific probe showed that the rRNA processing defect was not sufficient to diminish steady state levels of 18S rRNA.

Finally, to obtain an unbiased overview of the transcriptional changes that occurred in response to hepatic Rps6 deficiency, we performed gene expression microarray analysis using mRNA isolated from livers of 5 week old WT and ΔS6 mice which showed that loss of Rps6 perturbed the expression of a large number of mRNAs (S7A Fig). Applying a ≥ 8-fold up or down cut-off for differential mRNA expression, we determined that Rps6-deficiency resulted in the differential expression of 235 mRNAs, of which 184 were upregulated and 52 were downregulated (S1 Table). While the most highly expressed mRNAs in ΔS6 livers included oncofetal and imprinted genes (eg. *H19*, *Bex1* and *Igf2*) or mRNAs that are known to be expressed in immature hepatocytes or liver progenitor cells (eg. *Afp*, *Nope*, *Cd24a*, *Tacstd1*/*Epcam*, *Sox9* and *Krt19*), mRNAs that were downregulated included mature liver genes such as cytochrome P450s and major urinary proteins (*Mup*s), consistent with a shift to a more de-differentiated, immature liver. Classical p53 targets including the cell cycle inhibitor *p21/Cdkn1a*, *Sox4*, a protein required for stabilization of p53 during checkpoint activation, and *Noxa* and *Peg3*, which mediate p53-dependent cell cycle arrest and apoptosis respectively, were also among the upregulated mRNAs. However, an additional group of mRNAs signifying activation of innate immunity and the senescence-associated secretory phenotype or SASP that included chemokines (*Cxcl13*, *Cxcl14)*, damage associated molecular patterns or DAMPs (*S100a8*, *S100a9*, *S100a6*, *S100a11 and S100a14*) and NF-κB-associated genes (*Dmbt1*, *Tff3*, *Muc1* and *Sprr2a*) was also upregulated in ΔS6 livers. Bioinformatics analysis using Ingenuity Pathway Analysis software identified signaling networks associated with cell cycle arrest/senescence (p21/Cdkn1a, MAPK), regeneration (Jun/Spp1) and inflammation/innate immune system activation (NF-κB) as the most prominent networks associated with altered gene expression in ΔS6 livers (S7B Fig). These results confirm that loss of hepatic Rps6 activates p53 and disrupts rRNA processing and that regeneration in ΔS6 livers reflects an attempt to re-establish homeostasis in the face of cell cycle arrest/senescence, injury and inflammation.

### Rps6 is required for hepatocyte survival in adult liver

**Fig 4:**
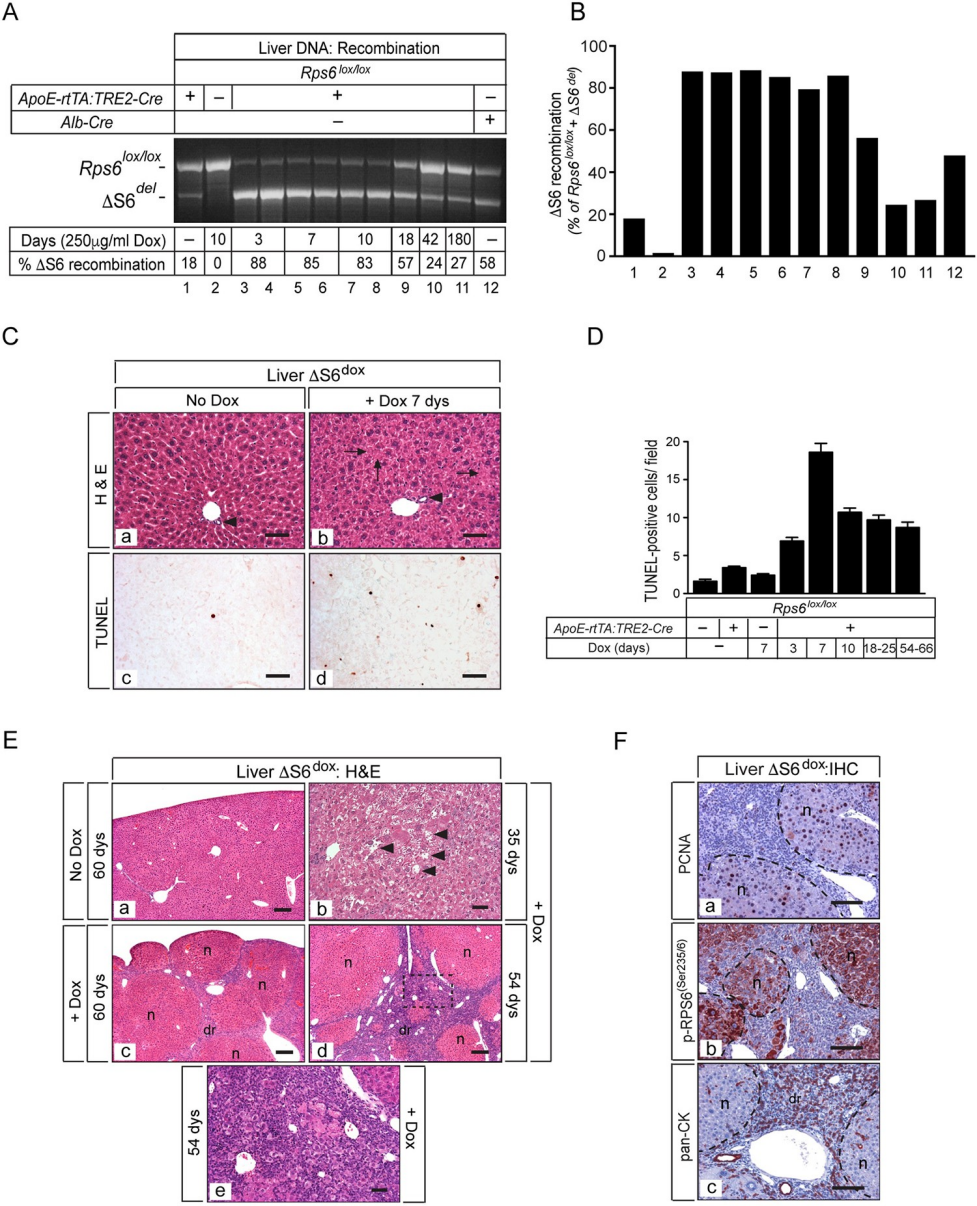
Deletion of *Rps6* in adult liver induces hepatocyte apoptosis and triggers regeneration. (A) Ethidium-stained gel showing recombination of the ΔS6 allele in livers of ΔS6^dox^ mice with and without dox (250μg/ml) for different lengths of time. (B) Graph of quantitation of % ΔS6 recombination from gel shown in A). While there is a low level of recombination (~18%) in the absence of dox (lane 1), it increases to ~85% when mice are provided with 250μg/ml dox representing loss of Rps6 in a majority of hepatocytes, the most abundant cell type in the liver (lanes 3–8). After ~3 weeks, the extent of recombination begins to decrease reflecting the loss of Rps6-deficient hepatocytes and their replacement with Rps6-expressing cells (lanes 9–11) (see F below). Note that the degree of recombination achieved in livers of *Rps6*^*lox/lox*^:*ApoE-rtTA*:*TRE-Cre* mice (provided with dox) before widespread hepatocyte loss occurs (lanes 3–8) is consistently greater than that achieved in livers of *Rps6*^*lox/lox*^:*Alb-Cre* mice (lane 12). (C) Photomicrographs of H&E staining (a and b) or TUNEL staining (c and d) on sections of ΔS6^dox^ livers provided with water (a, c) or water containing 250μg/ml dox (b, d) for 7 days (Original magnifications, all x187; scale bars 50μ). While bile ducts are present and appear normal in ΔS6^dox^ livers (arrow heads), hepatocytes appear disordered, show mild vacuolization and some lack nuclei (arrows). (D) Graph showing quantitation of number of TUNEL-positive cells/field in liver sections of WT and ΔS6^dox^ mice provided with dox for different lengths of time. (E) Photomicrographs of H&E stained sections of ΔS6^dox^ livers provided with water (a) or 250μg/ml dox for different lengths of time (b-f). After ~35 days on dox, hepatocyte blebbing and focal drop-out is visible (b), followed by a full-blown regenerative response involving the emergence of regenerative nodules (n) and a ductular reaction (dr) (c-e). Image in (e) is a higher magnification of the area in (d) bounded by the dotted line (Original magnifications, a, c, d (x31.25); b, e (x125)). Scale bars: a), c) and d), 100μ; b) and e), 50μ. (F) Photomicrographs of IHC for PCNA, phospho-RPS6^(Ser235/6)^ and pan-cytokeratin (CK) on livers of ΔS6^dox^ mice provided with dox for 54 days showing that regenerating nodules contain PCNA- and phospho-RPS6^(Ser235/6)^-positive immature hepatocytes while pan-CK-positive cells comprise the dr. Original magnifications, all x125; scale bars, 50μ.

Our results showing that loss of Rps6 in normal non-regenerating hepatocytes compromised cell viability contrasts with previous reports of Rps6-deficiency blocking hepatocyte proliferation post partial-hepatectomy [34] suggesting that the fate of Rps6-deficient hepatocytes is context- or stimulus-dependent. Moreover, because hepatocytes can be irreversibly damaged by the detergent action of hydrophobic unconjugated bile acids that accumulate in cholestatic disease [48], it was important to determine if the robust hepatocyte death that occurred in neonatal ΔS6 livers reflected an intrinsic dependency on Rps6 for survival, bile-acid induced hepatotoxicity, or a combination of both. To eliminate bile-acid mediated hepatotoxicity as a key mediator of hepatocyte death in ΔS6 livers, we adopted a strategy that allowed us to specifically delete Rps6 in adult hepatocytes while sparing the neonatal period and bypassing biliary development. This was done by breeding *Rps6*^*lox/lox*^ mice to a line of bi-genic mice that co-expresses a modified reverse tetracycline transactivator (rtTA_M2_) [49] driven by the liver-specific regulatory elements of the ApoE gene [50] (*ApoE-rtTA*_*M2*_) and Cre recombinase under control of the minimal CMV promoter and tetracycline responsive elements (S8 Fig) such that Cre could be activated in hepatocytes by providing mice with doxycycline (dox). Empirical determination of the minimal dose of dox required to achieve efficient hepatocyte-specific recombination of the *Rps6*^*lox/lox*^ allele (ΔS6^*del*^) showed that inclusion of dox at a concentration of 200–250 μg/ml in drinking water beginning at 5–6 weeks of age was sufficient to achieve 80–90% recombination in as little as 3–7 days, a significantly higher degree of recombination than had been achieved with Albumin-Cre, although recombination decreased over time (Fig 4A and 4B). Analysis of livers from WT and *Rps6*^*lox/lox*^:*ApoE-rtTA*:*TRE2-Cre* mice (herein referred to as ΔS6^dox^ mice) provided with drinking water without dox or supplemented with 200–250μg/ml dox for various lengths of time showed that, without dox, livers of ΔS6^dox^ mice were mildly discolored, but otherwise normal despite exhibiting a low level of “leaky” (dox-independent) recombination (Fig 4A and 4B). This was in contrast to livers of ΔS6^dox^ mice, which began to show evidence of cellular stress after just 7 days of dox treatment. While bile ducts were normal, hepatocytes appeared crowded due to the narrowing of sinusoidal spaces with some hepatocytes also appearing to lack nuclei suggestive of apoptotic cell death (panel b of Fig 4C). TUNEL analysis of livers from ΔS6^dox^ mice provided with dox for different lengths of time confirmed that hepatocytes were indeed apoptotic with death peaking ~1 week post-dox initiation and continuing at a lower level thereafter (panel d of Fig 4C and Fig 4D). Despite the low level of dox-independent recombination, livers of 8–9 week old ΔS6^dox^ mice without dox remained normal (panel a of Fig 4E). This was in sharp contrast to livers of ΔS6^dox^ mice provided with dox which showed extensive hepatocyte vacuolization and focal drop-out after ~5 weeks (panel b of Fig 4E), which triggered a full-blown regenerative response involving nodular growth (panel c of Fig 4E) and emergence of a dr (panel d of Fig 4E) that often co-existed with the presence of ballooning hepatocytes and piecemeal necrosis consistent with ongoing hepatocyte death (panel e of Fig 4E). Finally, to determine if the progressive decline in recombination of the ΔS6^del^ allele reflected repopulation of ΔS6^dox^ livers with Rps6-expressing cells to replace dying hepatocytes, we performed IHC with PCNA-, phospho^Ser235/6^-Rps6- and pan-CK-specific antibodies. Analysis showed that, like ΔS6 livers, regenerating ΔS6^dox^ livers also contained nodules comprised of PCNA-positive and phospho^Ser235-236^-RpS6-positive hepatocytes and a dr composed of phospho-Rps6-negative oval cells (panels a, b, and c of Fig 4F). While these results show that neonatal and adult hepatocytes both require Rps6 for survival, our results are consistent with the idea that the rapid and near-catastrophic hepatocyte death in neonatal ΔS6 livers reflects Alb-Cre- mediated depletion of Rps6 from both functional compartments of the liver which compounds disease due to exacerbation of an intrinsic susceptibility to death caused by exposure to toxic bile acids resulting from a failure to complete bile duct development.

### Chronic hepatic Rps6 deficiency predisposes to liver overgrowth and tumor development

**Fig 5:**
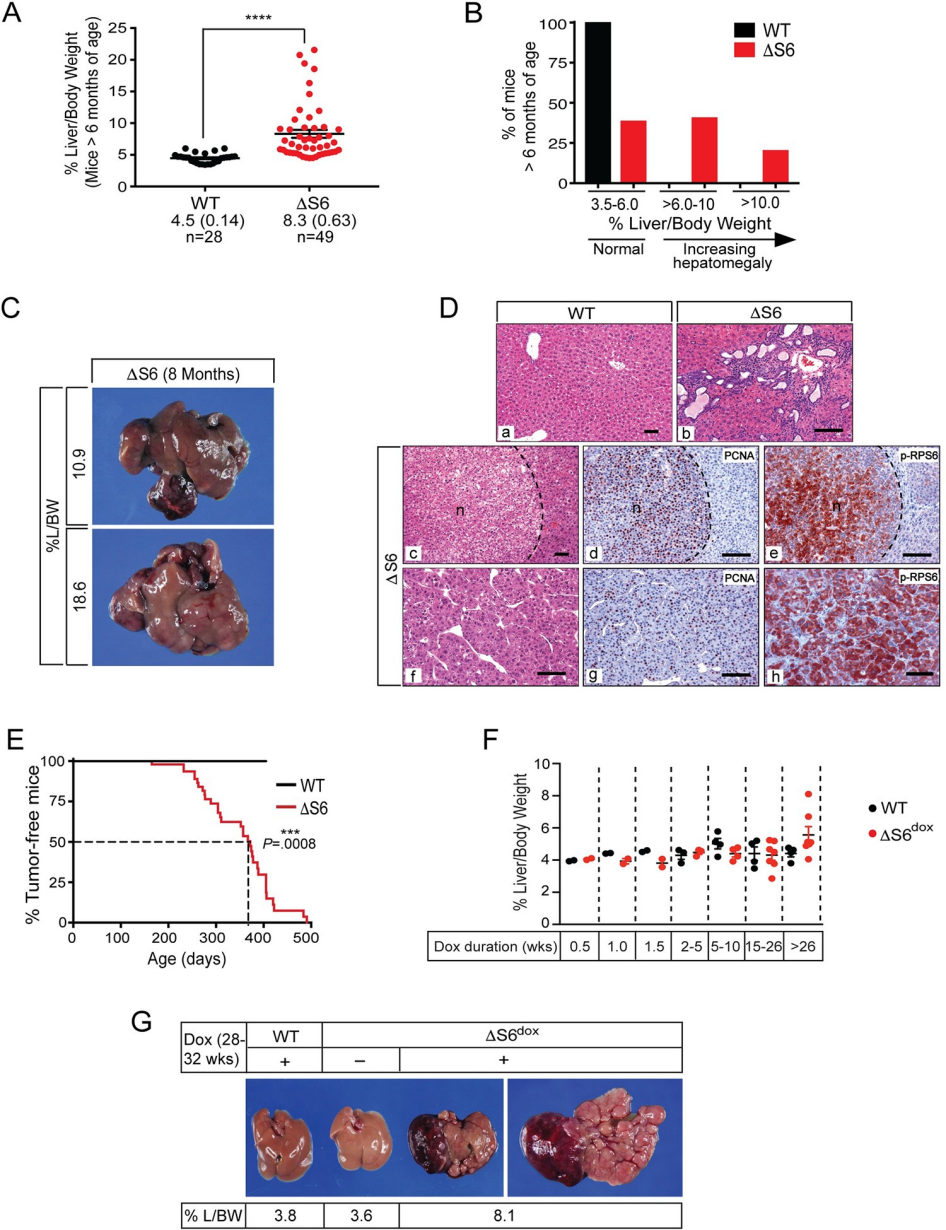
Rps6-deficient livers overgrow and are predisposed to spontaneous tumor development. (A) Graph of % liver/body weights (% L/BW) in WT and ΔS6 mice at ≥ 6 months of age showing that many ΔS6 livers have grown to exceed normal size. Data are mean (± SEM); **** *P* < .0001; 2-tailed unpaired Student’s *t*-test. (B) Graph showing % of WT and ΔS6 mice at ≥ 6 months of age with % L/BWs in normal range (3.5–6%) or larger than normal (> 6%). (C) Photomicrographs of livers from 2 ΔS6 mice at ~8 months of age. Livers have grown to 2–3 times normal size and are grossly misshapen due to abnormal nodular growth. (D) Photomicrographs of H&E stained slides and IHC of livers from WT (a) or ΔS6 mice at ≥ 6 months of age (b-h) showing bile duct hamartomas (b) and tumors with compact nodular (c) or trabecular growth patterns (d). IHC for PCNA (d and g) and phospho-RPS6^(Ser235/6)^ (e and h) show that tumors are comprised of highly proliferative cells that display activation of mTOR. AEC chromagen (red/brown); hematoxylin counterstain (blue). Original magnifications; a, c, x 62.5; b, d-h, x 125 (scale bars, all 50μ). (E) Kaplan-Meier curve showing % of WT and ΔS6 mice that are tumor-free at ≥ 6 months of age. 50% of ΔS6 mice have developed at least 1 tumor by 372 days. *P* = .0008 (Log-rank (Mantel-Cox) test). (F) Graph of %L/BWs of WT and ΔS6^dox^ mice showing that some ΔS6^dox^ livers (2/7 (28.6%)) have also overgrown after being maintained on dox for >26 weeks of age. While none of the dox treatment durations result in significantly different % L/BWs between WT and ΔS6^dox^ mice, there is a trend towards hepatomegaly in ΔS6^dox^ mice with increasing time on dox (2-tailed unpaired Student’s *t*-test). (G) Photomicrographs of livers from a WT mouse provided with 250μg/ml dox for 7 months (3.8% L/BW; left), a ΔS6^dox^ mouse provided with water (no dox) for 6.5 months (3.6% L/BW; middle) and a ΔS6^dox^ mouse provided with 250μg/ml dox for 7 months (8.1% L/BW; right (top of liver) and far right (underside of liver)) that shows hepatomegaly and aberrant nodular growth extending out from a lobe.

The transition from a hypoplastic, cytopenic state to a hyperplastic, cancerous state is a poorly understood aspect of the ribosomopathies. However, ribosomal protein haploinsufficiencies in model organisms have often resulted in overgrowth or cancer phenotypes indicating that mechanisms that normally regulate tissue homeostasis are lost, overridden or actively disabled in the context of chronic RiBi dysfunction. Because liver mass in the mouse is maintained at ~5% of body weight throughout adult life, we took advantage of this strict size control to determine if livers of ΔS6 mice overgrew or developed cancer as they aged. Analysis of liver mass in cohorts of WT and ΔS6 mice at ≥ 6 months showed that in contrast to WT mice, all of which had livers that remained within normal % Liver/Body Weight (%L/BW) range, ~60% of livers from ΔS6 mice displayed hepatomegaly ranging from mild to extreme (Fig 5A and 5B). In most cases, ΔS6 livers were grossly misshapen due to the aberrant growth of one or more lobes, many of which displayed nodular growth indicative of pre-malignant or malignant conversion (Fig 5C). Histological analysis of enlarged ΔS6 livers revealed the presence of biliary malformations such as bile duct hamartomas (panel b of Fig 5D) as well as adenomas that almost invariably contained PCNA- and phospho-Rps6^Ser235/6^-positive cells (panels c, d, and e of Fig 5D). However, most striking of all was our finding that malignant tumors, most of which were moderately differentiated hepatocellular carcinomas (HCCs) with solid or trabecular growth patterns that stained strongly for PCNA and phospho-Rps6^Ser235/6^ (panels f, g, and h of Fig 5D), developed in ~50% of ΔS6 livers by ~1 year of age (Fig 5E). Notably, although hepatomegaly only developed in 2/14 (14%) of ΔS6^dox^ mice maintained on dox for longer than 15 weeks, both cases (%L/BWs of 6.7% and 8.1%) occurred in mice that had received continuous dox treatment for 30 or 38 weeks respectively (Fig 5F) suggestive of a trend towards liver overgrowth as a function of increasing age or continued depletion of Rps6. Moreover, tumors developed in 6/14 (43%) of mice maintained on dox for >15 weeks, 4 (75%) of which developed in mice that had received dox for >26 weeks (Fig 5G). These results suggest that loss of Rps6 from adult hepatocytes disrupts liver homeostasis and predisposes to overgrowth and tumor development, the extent to which appears to depend on developmental context and timing of Rps6 depletion.

**Fig 6:**
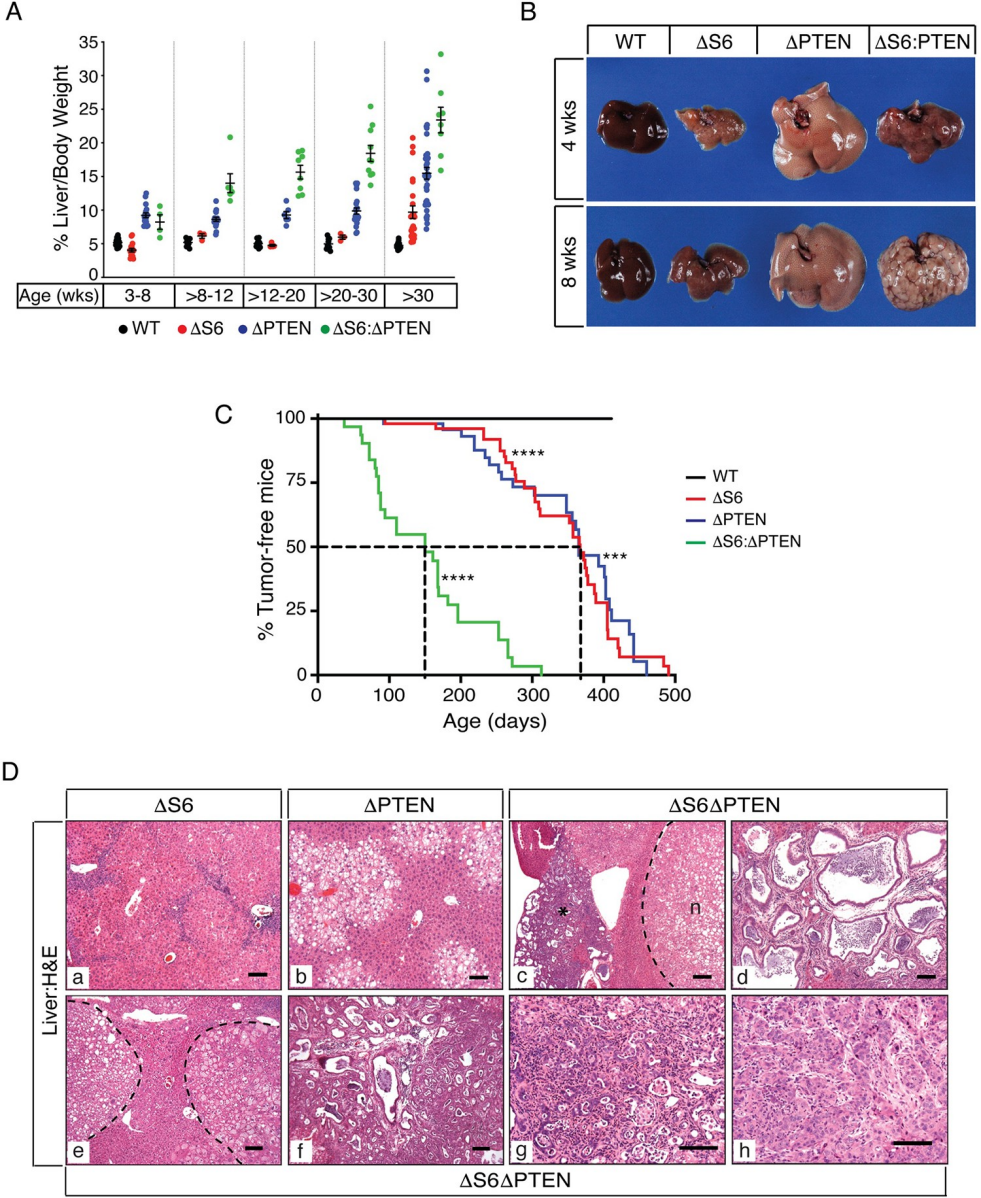
Rps6-deficiency lowers the threshold for tumor development in PTEN-deficient livers. (A) Graph of %L/BWs in WT, ΔS6, ΔPTEN and ΔS6ΔPTEN mice at different ages showing accelerated growth of ΔS6ΔPTEN livers from ~12 weeks of age. For mean %L/BWs and *P* values, please see S2 Table. (B) Gross appearance of WT, ΔS6, ΔPTEN and ΔS6ΔPTEN livers at 4 and 8 weeks of age. Note the dramatic change in appearance of the ΔS6ΔPTEN liver between 4–8 weeks age as fatty nodules develop. (C) Kaplan-Meier curve showing % of liver tumor-free WT, ΔS6, ΔPTEN and ΔS6ΔPTEN mice. Age at which 50% of ΔS6, ΔPTEN and ΔS6ΔPTEN mice develop at least 1 liver tumor is 367, 365 and 150 days respectively. ***, *P* = .0001; ****,*P* < .0001. (Log-rank (Mantel-Cox) test). (D) Photomicrographs of H&E stained liver sections from a) a 3 month old ΔS6 mouse showing regenerative nodules and remnants of the dr, b) a 5.5 month old ΔPTEN mouse showing typical pericentral steatosis, c) a 5.5 month old ΔS6ΔPTEN mouse showing a nodule (n) compressing the parenchyma adjacent to an area displaying bile duct hamartomas (*), d) a 5.5 month old ΔS6ΔPTEN mouse with a bile duct hamartoma, e) a 10 week old ΔS6ΔPTEN mouse with fatty adenomas, f) an 8 week old and g) a 3 month old ΔS6ΔPTEN mouse with cholangiocarcinomas, and h) an 11 month old ΔS6ΔPTEN mouse with a trabecular HCC (f). Dashed lines denote nodule boundaries. Original magnifications; a, b, c, d (x 62.5); e, f (x 56.25); g, h (x 125); Scale bars, all 50μ.

The hepatomegaly and spontaneous tumor development in ΔS6 livers prompted us to further explore the idea that hepatic Rps6 deficiency was a priming event for tumor development. We therefore lowered the threshold for malignant conversion in ΔS6 livers by co-opting the Alb-Cre transgene used to delete Rps6 to also delete the tumor suppressor Pten, a mild, yet reliable oncogenic stimulus, that by itself, results in delayed-onset liver tumor development in mice by ~1 year of age [51,52]. Analysis of recombination of the *Rps6*^*lox/lox*^ (ΔS6^del^) and *Pten*^*loxlox*^ (ΔPTEN) alleles in livers of WT, ΔS6, ΔPTEN and doubly deficient ΔS6:ΔPTEN mice demonstrated that Alb-Cre transgene expression was not limiting for recombination of either allele when both were present in the homozygous *(lox/lox)* state (S9A Fig). Western blotting of liver lysates from each of the parental strains using phospho-Rps6^Ser235/6^- and phospho-Akt^Ser473^-specific antibodies also confirmed that mTOR, but not Akt, was activated in ΔS6 livers, Akt, but not mTOR, was activated in ΔPTEN livers and that both pathways were active in ΔS6:ΔPTEN livers (S9B and S9C Fig). Monitoring of cohorts of WT, ΔS6, ΔPTEN and ΔS6:ΔPTEN mice up to ~1 year of age for signs of hepatomegaly or tumor development showed that, as previously documented, livers of ΔS6 mice that had initially been smaller than normal began to grow and exceed normal size after ~30 weeks of age (Fig 6A and S2 Table). Conversely, ΔPTEN livers, which demonstrate PI3K/Akt driven metabolic changes that promote steatotic hepatocyte hypertrophy, were consistently larger than normal from the outset. However, the growth of ΔS6:ΔPTEN livers began to diverge from each of the parental strains and accelerate at ~8–12 weeks of age, at which time livers became peppered with pale colored nodules that covered the surface (Fig 6B). Sampling of livers from mice of all genotypes at different ages revealed that loss of both Rps6 and Pten accelerated tumor development relative to each of the parental strains to the extent that 50% of ΔS6:ΔPTEN livers had developed at least 1 tumor by ~20 weeks age (Fig 6C) with 1 mouse developing tumors as early as 8 weeks of age. ΔS6:ΔPTEN livers also displayed other hyperproliferative lesions and abnormalities including bile duct hyperplasia and dysplasia, biliary hamartomas, duct ectasia, bile infarcts, altered hepatic foci and fatty adenomas, many of which were present in mice <6 months of age (Fig 6D). Notably, in contrast to ΔS6 livers which typically developed HCCs, ΔS6:ΔPTEN livers developed a wider spectrum of tumors including cholangiocarcinomas and hepatocholangiocellularcarcinomas (S3 Table and Fig 6D). Thus, Rps6-deficiency disrupts normal hepatic homeostasis and primes the liver for malignant conversion and accelerates liver tumor development in the context of hyperactivated PI3K/Akt-mediated growth factor signaling.

### Overexpression of Myc preserves hepatocyte viability and eliminates the need for neonatal ΔS6 livers to regenerate but fails to prevent malignant progression

**Fig 7:**
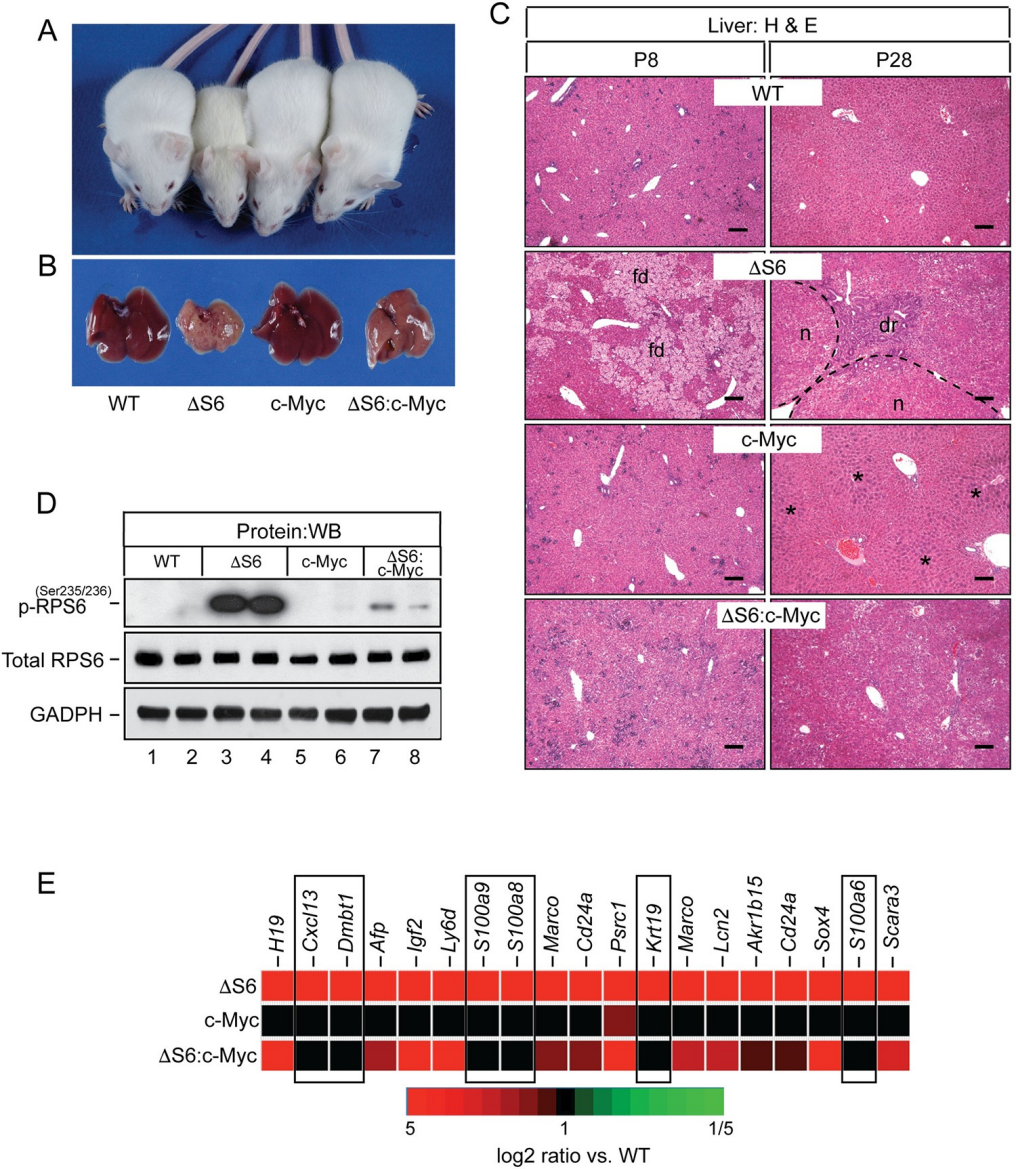
Overexpression of c-Myc rescues the growth defect and eliminates the requirement for ΔS6 livers to regenerate by preserving hepatocyte viability. (A) Picture of a 28 day old WT, ΔS6, c-Myc and ΔS6:c-Myc mouse. The ΔS6:c-Myc mouse (far right) is indistinguishable from the WT mouse (far left) in terms of size and lacks the jaundiced (yellowed) coat of the ΔS6 mouse (second from left). (B) Gross appearance of livers from 28 day old WT, ΔS6, c-Myc and ΔS6:c-Myc mice. Note the smooth and less jaundiced appearance of the ΔS6:c-Myc liver relative to the ΔS6 liver. (C) Photomicrographs of H&E stained liver sections of 8 and 28 day old WT, ΔS6, c-Myc and ΔS6:c-Myc mice showing the absence of feathery degeneration at P8 and lack of regenerative nodules or a dr at P28 in livers of ΔS6:c-Myc compared to ΔS6 mice. Dotted lines depict nodule boundaries. Asterisks (*) denote characteristic regions of hepatocyte hypertrophy in Alb-c-Myc livers (Original magnifications, x 62.5; scale bars 50μ). (D) Western blot showing that mTOR-dependent phosphorylation of RPS6 is suppressed by overexpression of c-Myc in ΔS6:c-Myc livers. GAPDH; protein load control. (E) Heat map showing that overexpression of c-Myc normalizes the innate immunity molecular signature comprising NF-κB target genes and DAMPs (boxed areas), but not imprinted genes, in ΔS6 livers.

Many studies have established bi-directional crosstalk between c-Myc and ribosomal proteins in the regulation of ribosome biogenesis, cell growth and cancer [53–58]. Indeed, decreased expression of c-Myc has been implicated as a driver of pancreatic hypoplasia in a mouse model of Schwachman-Diamond Syndrome (SDS) (OMIM #260400) [59]. Because c-Myc is highly expressed in fetal hepatoblasts of the developing liver yet drops to virtually undetectable levels by birth [60], we were unable to determine if hypoplastic neonatal ΔS6 livers expressed less c-Myc than their WT counterparts. However, array analysis did indicate that *Myc* mRNA was elevated in ΔS6 livers during the regenerative phase despite failing to reach the 8-fold cut-off to be included in the list of differentially regulated genes (S1 Table). Northern blotting confirmed that *Myc* was modestly elevated (~3-5-fold) in ΔS6 livers (S10A and S10B Fig), with IHC with c-Myc-, pan-CK and Rps6-specific antibodies revealing the source of increased c-Myc to be pan-CK-positive ductular cells rather than Rps6-expressing nodular hepatocytes suggesting that nodular growth in regenerating ΔS6 livers was not being driven by an increase in c-Myc (S10C and S10D Fig). Given c-Myc’s role in positively regulating ribosome biogenesis and that rp haploinsufficiency constrains Myc-dependent oncogenesis *in vivo* [53], we asked how increasing the level of c-Myc in the liver altered its response to Rps6-deficiency either during the early hypoplastic phase or the later tumor-prone phase. This approach involved breeding ΔS6 mice to *Albumin-c-Myc* (*Alb-c-Myc*) transgenic mice [61] which express a modest level of c-Myc (~8-fold above normal) in postnatal hepatocytes (S10A and S10B Fig) that is sufficient to stimulate ribosome biogenesis and promote hepatocyte hypertrophy in livers of young mice, but unable to drive fully penetrant HCC development before 1 year of age. Analysis of progeny from matings arranged to generate *S6*^*lox/lox*^:*Alb-Cre*:*Alb-c-Myc* mice (herein referred to as ΔS6:c-Myc mice) confirmed that ΔS6:c-Myc mice were born at the expected frequency. The first indication that elevated c-Myc was altering the liver’s response to Rps6 deficiency came after analyzing recombination of the ΔS6^*del*^ allele which showed that it had increased from ~50% in ΔS6 livers to ~80% in ΔS6:c-Myc livers (S11A Fig), an effect that was not seen in livers of ΔS6:ΔPTEN mice (S11B Fig). Northern blotting confirmed that this increase in abundance of the recombined ΔS6^*del*^ allele in ΔS6:c-Myc livers translated into a further reduction in the level of Rps6 mRNA causing it to fall below the level required to sustain normal levels of 18S rRNA (S11C Fig). Given that ΔS6:c-Myc livers expressed less Rps6 than their ΔS6 counterparts, we expected neonatal ΔS6:c-Myc mice to be just as small, if not smaller than ΔS6 mice. However, to the contrary, ΔS6:c-Myc mice were visually indistinguishable from their WT or Alb-c-Myc littermates (Figs 7A, and S12A and S4 Table). Moreover, livers of young adult ΔS6:c-Myc mice were neither small nor mottled (Figs 7B and S12B) suggesting that c-Myc was suppressing, rather than enhancing the neonatal growth defect and hepatic dysfunction in ΔS6 mice. Histological evaluation of ΔS6:c-Myc livers at P7 showed the complete absence of cholestatic hepatocyte degeneration indicating that c-Myc was exerting a hepatoprotective effect by neutralizing the hepatocyte death caused by loss of Rps6 (Fig 7C). Consequently, ΔS6:c-Myc livers no longer needed to regenerate as seen by the absence of regenerative nodules or evidence of a ductular reaction (Fig 7C). Unexpectedly, we found that in contrast to Rps6 mRNA levels which were decreased in ΔS6:c-Myc livers (S11D Fig), RPS6 protein levels remained unchanged (Fig 7D), suggesting that one mechanism by which c-Myc could be neutralizing hepatocyte death in ΔS6 livers was by influencing the post-transcriptional processing of *Rps6*, either at the level of splicing or translation. In searching for molecular correlates of c-Myc-dependent hepatoprotection, analysis showed that c-Myc blunted Rps6-phosphorylation/mTOR activation (Fig 7D) and normalized the expression of mRNAs associated with activation of NF-κB and induction of the innate immune response in ΔS6 livers (Figs 7E and S11D). However, it failed to normalize the expression of oncofetal genes such as *H19* or *Igf2* or the classical p53-dependent targets *p21/Cdkn1a* and *Sox4* (Figs 7E and S11D). Finally, to determine if c-Myc overexpression altered progression to hepatomegaly or malignancy in ΔS6 livers, we monitored cohorts of ΔS6:c-Myc mice at ≥ 6 months of age. Analysis showed that livers of ΔS6:c-Myc mice had a higher propensity to develop moderate to severe hepatomegaly than their ΔS6 or Alb-c-Myc counterparts (S13A and S13B Fig) and that tumors developed slightly earlier in ΔS6:c-Myc mice relative to each of the parental strains (S13C Fig), but not as early as ΔS6:ΔPTEN mice (Fig 6C). Thus, while a modest increase in c-Myc alters the fate of neonatal hepatocytes in ΔS6 livers by preserving hepatocyte viability and eliminating the need for ΔS6 livers to regenerate, it is unable to overcome all of the derangements caused by Rps6 insufficiency and fails to prevent liver overgrowth or tumor development as mice age.

### Hepatoblast-specific expression of p53^QS^ mimics the biliary, but not the hepatocyte defect in ΔS6 livers

**Fig 8:**
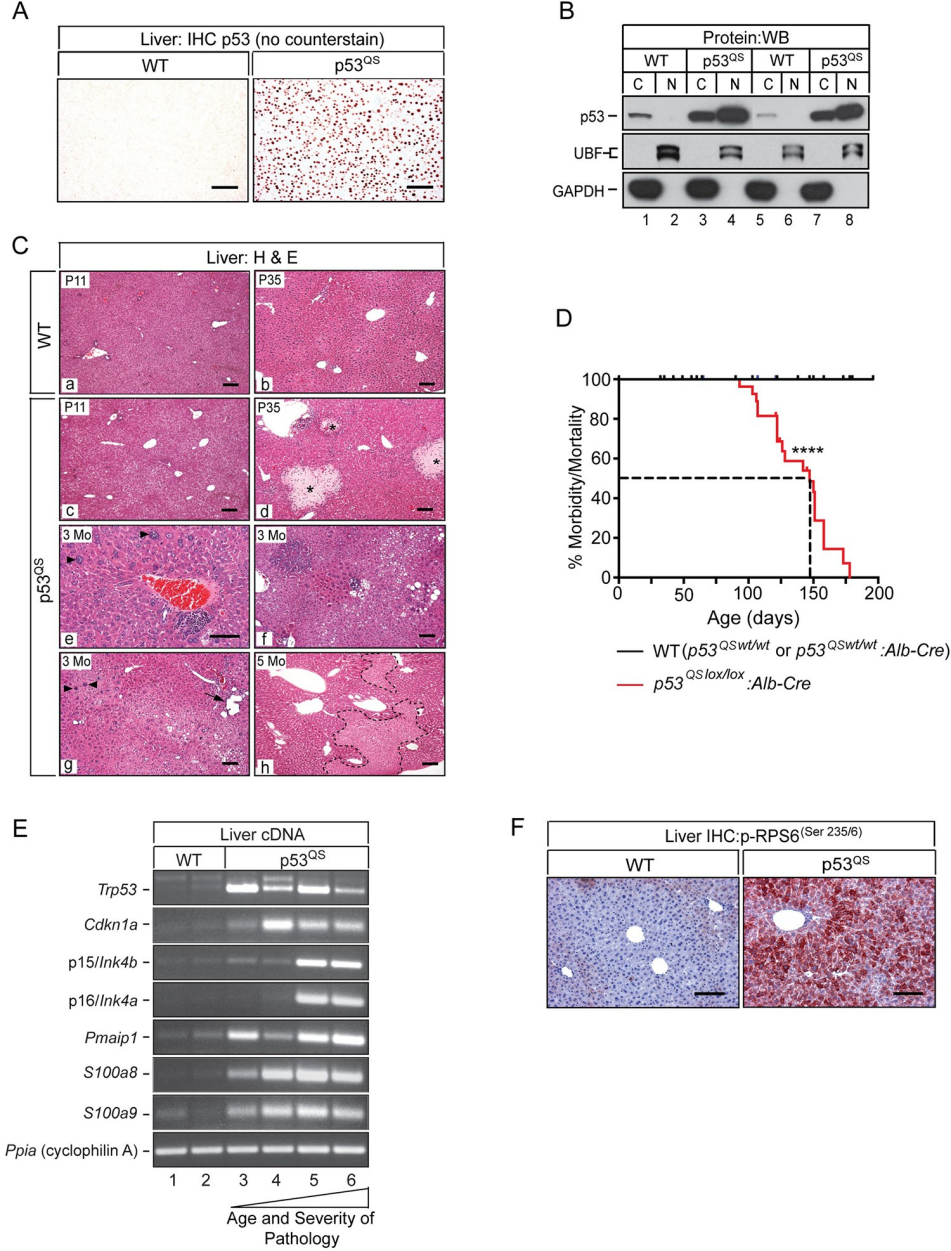
Hepatoblast-specific expression of p53^QS^ results in chronic liver failure preceded by induction of senescence and innate immunity and activation of mTOR. (A) p53 IHC performed on liver sections from a WT (left) and p53^QS^ expressing mouse (right) showing robust nuclear expression of the p53^QS^ mutant in hepatocytes. Original magnifications, x125. (AEC Chromogen (orange/red), no counterstain). (B) Western blot of fractionated cytoplasmic (C) and nuclear (N) proteins isolated from the livers of 2 WT mice (lanes 1, 2, 5 and 6) and 2 p53^QS^ mice (lanes 3, 4, 7 and 8) with a p53-specific antibody. Immunoblotting of the same lysates using antibodies specific for the nuclear protein UBF (upstream binding factor) and the cytoplasmic protein GADPH confirm enrichment of nuclear and cytoplasmic proteins after fractionation. (C) H&E stained sections of WT (a, b) and p53^QS^ livers (c-h) showing age-dependent progression of disease in p53^QS^ livers. Regional hepatocyte vacuolization, evident at P11 (c) is followed by focal hepatocyte necrosis (biliary infarcts, *) by ~5 weeks of age (d). After ~3 months, hepatocytes show increasing heterogeneity across the lobule (e, f, g). Karyomegaly (enlarged nuclei (arrowheads, e and g)), regional hepatocyte vacuolization and biliary dilatation (g, arrow) are common. By ~5 months of age, p53^QS^ livers show evidence of widespread parenchymal loss (h, necrotic areas bounded by dashed lines) signifying ongoing liver decompensation in the absence of regeneration. Original magnifications; a, b, c, d, g, h and f, x62.5; e, x125. (D) Kaplan-Meier curve of morbidity and mortality in WT and *p53*^*QSlox/lox*^:*Alb-Cre* mice. Median survival of *p53*^*QSlox/lox*^:*Alb-Cre* mice; 147 days. **** *P* < .0001 (Log Rank (Mantel Cox) test). (E) Ethidium-stained gels of Sq-PCR for p53, *p21/Cdkn1a*, *Noxa*, senescence markers (*p15*^*INK4b*^ and *p16*^*INK4a*^) and DAMPs (*S100a8* and *S100a9*) in two 4–6 week old WT mice (lanes 1 and 2), two 4–6 week old p53^QS^ mice with moderate disease (lanes 3 and 4) and two 4 month old p53QS mice with advanced disease (lanes 4–6). (F) IHC with a phospho-RPS6^(Ser235/6)^-specific antibody showing regional phospho-RPS6^(Ser235/6)^ staining in WT liver and pan-lobular staining in p53^QS^ livers. Original magnifications, x125. (AEC Chromogen red, hematoxylin counterstain, blue). Scale bars for all images, 50μ.

Having confirmed that p53 had been stabilized in ΔS6 livers, we set out to address p53’s role in the failure of ΔS6 livers to form bile ducts or maintain hepatocyte viability by generating mice in which p53 is artificially stabilized in hepatoblasts of the developing liver. This was done using a strain of mice that expresses a conditional (lox-stop-lox (LSL)) knock-in mutant allele of *Trp53* harboring substitutions at amino acids L25Q and W26S (*p53*^*LSL-Q25S26*^) that abolishes binding to p53’s negative regulator Mdm2 [62]. Although this mutant is hypomorphic for transactivation of a select cadre of p53 target genes, it retains DNA binding capability [63] and has been shown to be sufficient to phenocopy the pigmentation defects that develop in *Dsk* mouse mutants harboring naturally occurring mutations in *Rps19* and *Rps20* or genetically engineered mice with keratinocyte-specific deletion of *Rps6* [64]. Successive rounds of breeding of *p53*^*LSL-Q25S26*^ mice to *Alb-Cre* mice produced mice in which both copies of the p53 mutant were targeted to hepatoblasts in an otherwise p53-null background (herein referred to as p53^QS^ mice). Monitoring of litters from birth showed that female mice that were homozygous for the mutant *p53* allele irrespective of Alb-Cre status were underrepresented in litters, consistent with previously reported lethality associated with *trp53-*nullizygosity in females [65,66]. Male p53^QS^ mice were, however viable and although body weights trended lower than their WT counterparts, statistical significance was not reached indicating that hepatic expression of p53^QS^ did not stunt neonatal growth (S14A Fig). IHC (Fig 8A) and immunoblotting (Fig 8B) confirmed that p53^QS^ was robustly expressed in >95% of hepatocytes, the majority of which was localized to the nucleus with a smaller amount present in the cytoplasm reflecting nucleo-cytoplasmic shuttling of p53 between both compartments. While gross inspection of livers of neonatal p53^QS^ mice revealed discoloration indicative of mild jaundice, livers were otherwise unremarkable in that they were neither small (S14B Fig) nor mottled. Moreover, histological evaluation showed that in contrast to ΔS6 livers which were already losing hepatocytes by 2 weeks of age (panels c and f of S14C Fig), p53^QS^-expressing hepatocytes remained viable (panels b and e of S14C Fig), with small bile-acid induced infarcts only appearing after mice reached adulthood (panel d of Fig 8C and panel h of S14C Fig). Biochemical analysis of liver function in p53^QS^ mice revealed evidence of hepatocellular and biliary dysfunction at 4–5 weeks of age, both of which had improved, but not completely resolved by 7–9 weeks of age (S15 Fig). In stark contrast to ΔS6 livers in which liver function had improved commensurate with nodular growth and induction of a robust dr (panel i of S14C Fig), liver function in p53^QS^ improved without any histological evidence of a regenerative response suggesting that livers were able to tolerate and adapt to p53^QS^ expression much better than loss of Rps6 (panel h of S14C Fig). In determining the basis for the biliary dysfunction in p53^QS^ livers, we performed Sox9 IHC which showed that p53^QS^ livers had fewer Sox9-expressing ductal plate cells at P8 and P15 than WT livers, and that of those that were visible, none were being incorporated into bile ducts (S16A-S16C Fig). However, in contrast to ΔS6 livers which had already begun to show signs of a nascent Sox9-positive dr by P15 (Fig 2A), this response was both delayed and muted in p53^QS^ livers (S16D Fig).

Having found that p53^QS^ expression generally mimicked the biliary defect, but was not able to induce the robust or widespread hepatocyte loss that accompanied loss of Rps6, we continued to monitor p53^QS^ mice to determine how chronic stabilization of p53 impacted the liver as mice aged. While p53^QS^ livers showed evidence of mild hepatic dysplasia and focal necrosis at ~5–6 weeks of age (panel d of Fig 8C), livers became increasingly unstable over time as seen by increasing hepatocyte heterogeneity in terms of size, nuclear morphology and degree of vacuolization. Moreover, small foci of atrophic or dying hepatocytes that had been evident at 4–6 weeks continued to expand to encompass large swathes of the parenchyma suggesting that livers were beginning to fail (panels e, f, g, and h of Fig 8C). Indeed, all p53^QS^ mice ultimately became moribund, requiring euthanization at or before 6 months of age (median survival: 147 days. Fig 8D). Autopsies performed on three p53^QS^ mice between 3–5 months of age revealed hepatomegaly and abnormal liver growth with histological analysis showing that much of the parenchyma had been replaced by small hepatoblast-like cells (panels a and b of S17 Fig). IHC with the p53-specific antibody revealed that liver failure in p53^QS^ mice was being driven by the death of p53^QS^-expressing hepatocytes with livers being repopulated by small immature cells that failed to express the p53^QS^ mutant (panels c and d of S17 Fig). Immunoprofiling of these p53^QS^-naïve cells showed that all expressed MYC (panels e and f of S17 Fig), while a subset also expressed EPCAM (panels g and h of S17 Fig) suggesting that p53^QS^ livers were being repopulated by maturation-arrested cells with immunoprofiles similar to, but distinct from E9.5-E11.5 (EPCAM+) or E12-14 (EPCAM-) hepatoblasts.

The protracted viability of p53^QS^-expressing hepatocytes together with the absence of any histological evidence of regeneration in livers of young p53^QS^ mice suggested the possibility that hepatocytes were being driven into a state of cell-cycle arrest or senescence before livers failed. We therefore performed Sq-PCR analysis of cell-cycle-, senescence- and apoptosis-associated genes *p21/Cdkn1a*, *p15/Ink4b*, *p16/Ink4a* and *Noxa* in livers of mice at different ages and stages of disease to determine if this was the case and, if so, how changes in expression correlated with disease as livers progressed from dysplasia to failure. Analysis showed that *p21/Cdkn1a* and *Noxa* were elevated in p53^QS^ livers irrespective of age or stage of disease (Fig 8E), a finding that was unexpected given that the p53^QS^ mutant is defective for transcriptional activation of both of these genes [63]. *p15/Ink4b* and *p16/Ink4a* were also induced in p53^QS^ livers; however unlike *p21/Cdkn1a* and *Noxa*, upregulation was only seen in end-stage livers containing immature p53^QS^-naïve cells. In light of the fact that senescence can induce innate immunity and the SASP [67,68], we analyzed mRNA expression of the DAMPs *S100a8* and S100a9, both of which had been induced in ΔS6 livers, and found that like *Cdkn1a* and *Noxa*, both were upregulated in p53^QS^ livers independent of age or disease state (Fig 8E). Finally, given mTOR’s ability to promote senescence and the SASP [69,70] we performed IHC with the phospho-Rps6^Ser235/236^-specific antibody on sections of WT and dysplastic p53^QS^ livers from 3 month old mice which revealed strong pan-lobular phospho-Rps6 staining in p53^QS^ liver indicating activation of mTOR/S6K signaling (Fig 8F). Taken together, these results show that while expression of p53^QS^ mimics loss of Rps6 by inhibiting bile duct development, hepatocytes tolerate p53^QS^ much better than loss of Rps6 and only die after a protracted period of cell-cycle arrest or senescence. Hepatocytes and biliary cells thus diverge in their response to p53 stabilization or loss of Rps6.

### Loss of p53 fails to improve liver disease in ΔS6 mice

**Fig 9:**
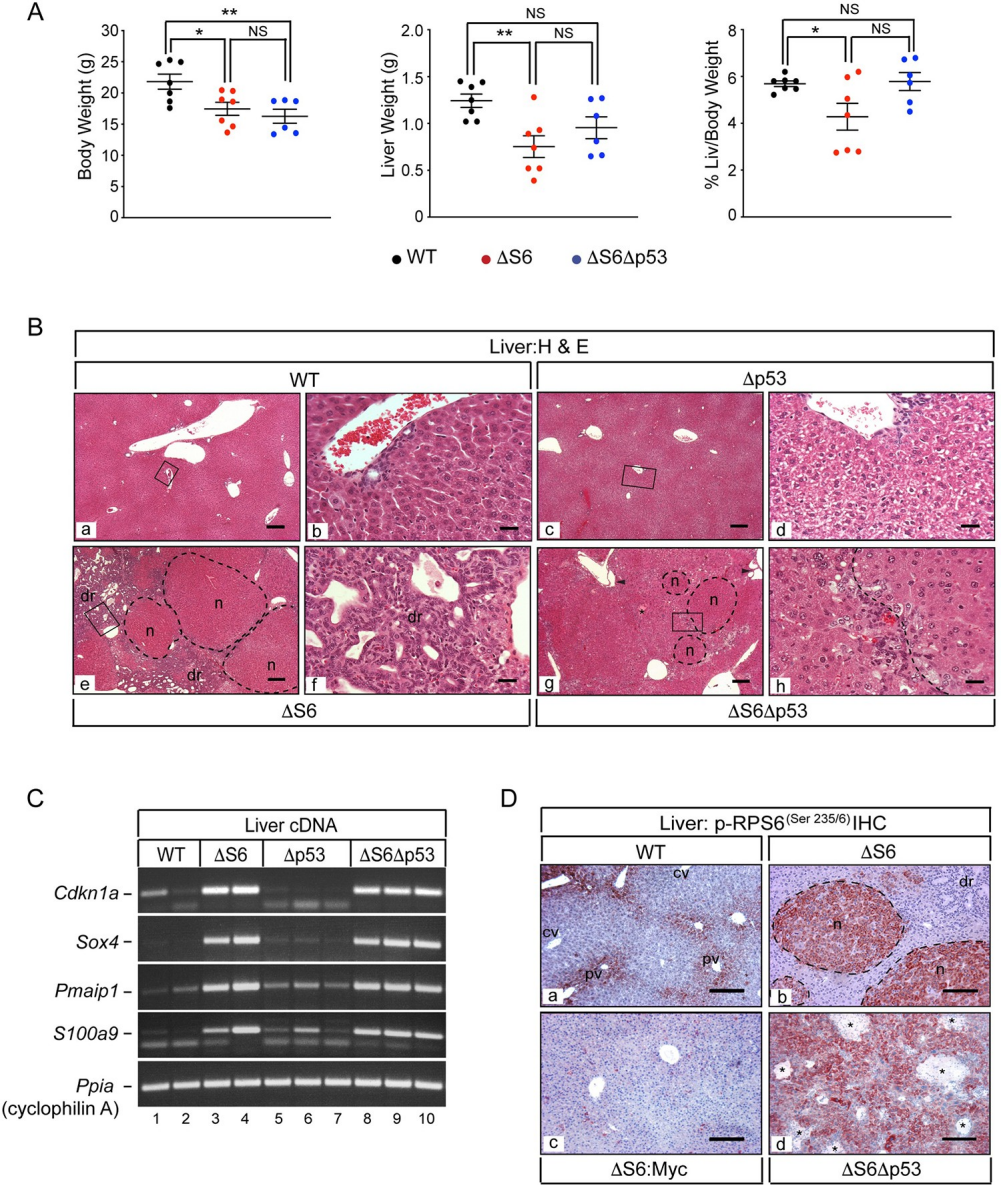
Loss of p53 does not rescue the ΔS6-associated growth defect or hepatic dysfunction. (A) Graphs of body weights, liver weights and % liver/body weights of 32–40 day old WT, ΔS6 and ΔS6Δp53 mice. *P* values: *P ≤ .05, **P ≤ .01. NS; not significant (2-tailed unpaired Student’s *t*-test). (B) Photomicrographs of H&E stained sections of livers from 4–5 week old WT, ΔS6, Δp53 and ΔS6Δp53 mice showing normal architecture and the presence of bile ducts in WT (a, b) and Δp53 (c, d) livers and well defined nodules and a prominent dr in the ΔS6 liver (e, f). Note moderate hepatocyte vacuolation in Δp53 livers (d). In the ΔS6Δp53 liver (g, h), nodules are present but are less defined due to the absence of a prominent dr which accentuates nodular boundaries in ΔS6 livers. Normal bile ducts are absent and biliary malformations are evident (arrow heads). Original magnifications; a, c, e and g (x 31.25; scale bars 100μ), b, d, f and h (x 250; scale bars 25μ). (C) Ethidium stained gels of sq-PCR performed on cDNA prepared from individual WT, ΔS6, Δp53 and ΔS6Δp53 livers. Note that loss of p53 fails to normalize the expression of any of the mRNAs induced in ΔS6 livers including *p21/Cdkn1a*. (D) IHC of livers of WT (a), ΔS6 (b), ΔS6:c-Myc (c) and ΔS6Δp53 (d) mice with a phospho-RPS6^(Ser235/6)^-specific antibody showing that mTOR activation in ΔS6 livers is suppressed by overexpression of c-Myc, but not by loss of p53. Original magnifications; a, c, d (x62.5; scale bars, 100μ), b (x125; scale bar 50μ). (AEC chromagen, red; hematoxylin counterstain, blue).

Having determined that expression of p53^QS^ mimicked the biliary, but not the hepatocyte defect in ΔS6 livers, we sought to determine the extent to which each aspect of liver disease depended on p53 by breeding *Rps6*^*lox/lox*^:*Alb-Cre* mice to *p53-/-* mice to generate mice with livers that were deficient for Rps6 and p53 (herein referred to as ΔS6:Δp53 mice). In setting benchmarks that would be used to determine the impact of p53 loss on ΔS6-associated hepatocyte death, we turned to ΔS6:c-Myc mice as our rescue paradigm given that augmentation of c-Myc in ΔS6 livers had preserved hepatocyte viability and corrected other hallmarks of Rps6-insufficiency by restoring normal liver mass and neonatal growth, suppressing the inflammatory signature and normalizing mTOR (Fig 7). Genotyping of progeny from the appropriate matings designed to generate ΔS6:Δp53 mice showed that mice lacking both copies of p53 were again underrepresented in litters irrespective of Rps6 status. Body weight analysis of WT, ΔS6 and ΔS6Δp53 mice between the ages of 4–6 weeks showed that ΔS6:Δp53 mice remained underweight indicating that the loss of p53 did not correct the neonatal growth deficit (Fig 9A). Moreover, although liver weights of half of the ΔS6:Δp53 mice remained within the normal range, the mean liver weight of the group was not significantly different from either ΔS6 mice or WT mice, suggesting that the impact of *p53*-nullizygosity on ΔS6-associated liver hypoplasia was mixed (Fig 9A). Moreover, because %L/BW is a function of body weight and liver weight, the skewing of ΔS6Δp53 liver weights towards normal coupled with their lower body weights translated into %L/BW values that were normal, precluding us from reaching a definitive conclusion as to whether loss of p53 impacted ΔS6-associated liver hypoplasia. In light of this ambiguity, we turned to LFTs to determine if loss of p53 improved hepatic function in ΔS6 livers. Of the 4 biochemical markers used to assess function, only alkaline phosphatase, a marker of cholestasis, showed a modest improvement in the absence of p53, while total bilirubin levels remained elevated and ALT and AST levels increased further indicating that loss of p53 was exacerbating hepatocyte dysfunction, rather than improving it (S18 Fig). Histological evaluation of ΔS6:Δp53 livers also confirmed that loss of p53 failed to confer the level of hepatoprotection afforded by c-Myc as livers were still being forced to regenerate (Fig 9B). However, nodules were smaller and appeared less distinct than in ΔS6 livers (panels g and h of Fig 9B), in large part due to lack of a robust dr that had accentuated nodule boundaries in ΔS6 livers (panels e and f of Fig 9B). Indeed, Sox9 IHC showed that while the cord-like streaming and duct-forming properties of Sox9-positive cells in ΔS6 livers (panel b of S19A Fig) clearly signified induction of a classical HPC-mediated-dr as a precursor to secondary bile duct development [42], none of the Sox9-positive cells in ΔS6:Δp53 livers were becoming incorporated into ductular structures (panels c and d of S19A Fig). Biliary malformations in the form of cysts in peri-portal areas (panel g of Fig 9B) and a paucity of normal Sox-9-expressing bile ducts indicated that loss of p53 was failing to correct the biliary defect (S19A Fig). Analysis showed that immature hepatoblast-like cells had also begun to accumulate in ΔS6:Δp53 livers by 4–5 months of age, which in some cases, were either proliferating in distinct clusters or had expanded to become the dominant cell-type (S19B Fig). Finally, sq-PCR analysis of *p21/Cdkn1a*, *Noxa* and *S100a9* mRNAs, all of which had been upregulated in ΔS6 livers and normalized by c-Myc, showed that all remained elevated in ΔS6Δp53 livers (Fig 9C), while immunoblotting showed that loss of p53 also failed to blunt Rps6 phosphorylation (Fig 9D). By showing that loss of p53 fails to significantly improve liver disease in ΔS6 mice, these results indicate that liver disease in ΔS6 is either p53-independent, or that p53 is but one arm of a much broader ribosomal stress response that drives disease in Rps6-deficient livers.

## Discussion

The number of disparate human diseases that comprise the ribosomopathies illustrates the breadth of the impact that dysfunctional ribosome biogenesis (RiBi) can have on almost every organ system in the body. Using a conditional approach to delete the *Rps6* gene in hepatoblasts of the embryonic liver or mature hepatocytes of the adult liver, we show that Rps6 is required for establishing and maintaining hepatic homeostasis and that during the perinatal period, a significant reduction, but not total loss of Rps6 is sufficient to induce a ribosomopathy-like phenotype that manifests as severe neonatal hepatic hypoplasia and cholestasis with a predisposition for tumor development. Moreover, deleting hepatic Rps6 prior to birth resulted in significantly greater hepatic dysfunction than deleting it in adult liver, a result that likely reflects a higher demand for RiBi during the first 4 weeks after birth; an energetically demanding period whereby liver mass increases at its fastest rate as the neonate adapts to shifting metabolic demands that occur at birth and at weaning [71] as they transition to solid food while bile duct development is being completed.

Our finding that hepatocytes are unable to survive without Rps6 contrasts with a previous study showing that loss of Rps6 inhibits hepatocyte proliferation during regeneration [34]. However, this result is consistent with reports of hepatocyte degeneration and increased cell turnover in livers lacking other RiBi-associated genes such as *Dkc1* (dyskerin) [72] or *Sbds* [73] even though loss of Rps6 proved to be far more deleterious to the liver than loss of either Dkc1 or Sbds. Given that experimental phenotypes resulting from the loss of different RiBi genes reflect their spatio-temporal expression as well as the choice of Cre driver [74] the differences between our studies and those of Volarevic et al. [34] and Finch et al. [73] can, in part, be reconciled by the fact that liver-specific Cre drivers that target genes in mature hepatocytes but not biliary cells were used, precluding assessment of the potential impact of their loss on the biliary compartment. As Rps6 is abundantly expressed in hepatocytes and biliary cells, our use of Alb-Cre to delete *Rps6* in hepatoblasts eliminated expression from both cell types, which likely compounded disease by exposing Rps6-deficient hepatocytes that were already intrinsically sensitized to death to hepatotoxic levels of bile acids which had accumulated as a consequence of the failure of ΔS6 livers to complete bile duct development. Importantly however, the ductopenia and cholestasis in ΔS6 livers contrasts sharply with the absence of any biliary abnormalities and relatively mild hepatic dysfunction observed in mice with Alb-Cre-mediated depletion of *Dkc1* which, like *Rps6*, also activates p53 and disrupts rRNA processing [72]. As Alb-Cre has the potential to also extinguish expression of Dkc1 in hepatoblasts, one possible explanation for the reported lack of biliary disease and generally milder phenotype in Dkc1-deficient livers is that it is expressed in hepatocytes, but not in biliary cells such that loss of hepatic Dkc1 perturbs hepatocyte turnover, but is of no consequence for the biliary compartment. However, disparate phenotypes could also reflect the collateral loss of functions of individual genes beyond their roles in RiBi. For instance, dyskerin is both a component of small nucleolar ribonucleoprotein complexes and a constituent of the telomerase complex [75] while Rps6 is a constituent of the 40S ribosome and a phosphorylation-dependent signaling effector for several protein kinase cascades, most notably the rapamycin-sensitive S6K branch of mTOR [44]. Given that the bulk of Rps6 in the majority of hepatocytes is phosphorylated, we have yet to rule out the possibility that functions that depend on post-translational modification of Rps6 downstream or independent of mTOR/S6K contribute to the loss of hepatocyte viability in ΔS6 livers [44,76–79].

To our knowledge, inhibition of bile duct development has not been reported in any other mouse models of genetically induced RiBi dysfunction. However, biliary disease in ΔS6 livers is highly reminiscent of the neonatal intrahepatic cholestasis and jaundice that is seen in patients with North American Indian Childhood Cirrhosis (NAIC) (OMIM #604901), a rare autosomal recessive ribosomopathy associated with homozygous missense mutations (R565W) in the Cirhin1A-encoding gene *CIRH1A/UTP4* [80]. Classification of NAIC as a ribosomopathy is based on functional studies showing that the *CIRH1A* mutation disrupts ribosome biogenesis by interfering with its ability to bind to NOL11, a component of the human ribosomal small subunit (SSU) processome [81,82]. While a pathognomonic role for the *CIRH1A* R565W mutation has yet to be formally demonstrated *in vivo*, compelling evidence that bile duct development is indeed sensitive to perturbations in a subset of RiBi genes comes from experiments showing that morpholino-based depletion of Cirhin1A in zebrafish results in biliary defects and cholestasis secondary to failed maturation of the intrahepatic bile ducts [83]. Although the mechanistic basis for the bile duct defect in ΔS6 livers remains to be determined, we note that, unlike hepatocytes, none of the Rps6 that is expressed in biliary cells is phosphorylated on Ser235/6 suggesting that the ductopenia is unlikely to reflect loss of Rps6’s phosphorylation-dependent functions. However, the paucity of Sox9-expressing biliary precursors and mature bile ducts in ΔS6 livers is strikingly similar to other models of RiBi dysfunction in which abnormal organ development stems from a deficiency of specific progenitor cell types due to aberrant translation of critically required transcription factors or signaling effectors that are normally translated at or near threshold levels [64,84,85]. Liver disease in NAIC overlaps with the hepatic component of Alagille syndrome (AGS; OMIM #118450), an autosomal dominant disease caused by mutations in the notch signaling effectors *JAG1* or *NOTCH2* [86–88] or the transcription factor *HNF1B* [89]. Overlap in hepatic phenotypes is also seen between ΔS6 mice and mice harboring loss-of-function alleles in genes encoding notch pathway signaling components [42,90–93] or certain liver-expressed transcription factors [40]. Given that bile duct development requires the highly coordinated and dynamic interplay between spatially-restricted transcription factors and signaling effectors of the notch, TGF-β, wnt and Hippo/YAP pathways [40], further work would determine if the cholangiopathy in ΔS6 livers stems from the cell-autonomous impact of Rps6 loss on presumptive cholangiocytes as they transition from hepatoblasts, or reflects a broader disruption of pan-hepatic gene expression or architecture such that the hepatic environment is no longer permissive for bile duct development.

That ΔS6 livers were disproportionately small-for-size during the neonatal period, yet overgrew and developed tumors as mice aged is reminiscent of the hypo- to hyper-proliferative transition that precedes tumor development in a subset of human ribosomopathies [25–27,94,95] and other experimental models of RiBi dysfunction [28,30,31,96–98]. While the fitness constraint imposed by dysfunctional RiBi is known to trigger compensatory mechanisms that not only limit the tissue damage caused by the death or senescence of cells exhibiting nucleolar or proteotoxic stress, but also stimulate cell proliferation, this can lead to replicational stress and inadvertently facilitate transformation as observed in DBA and SDS [99,100]. In considering what features of ΔS6 livers could render them tumor-prone, we were struck by the fact that that 80% of the adenomas/tumors that we surveyed not only expressed Rps6 but also demonstrated strong immunoreactivity for phospho-Rps6^Ser235/6^ relative to adjacent liver. Given that mTOR activation and loss of p53 have both been reported to bypass or overcome ribosomopathy-induced cell cycle arrest or apoptosis [101–103], a logical question is whether a subset of tumors that develop in ΔS6 livers originate from Rps6-expressing/mTOR-activated cells that persist after the regenerative phase and gain a growth advantage [101,104]. Experimental evidence for mTOR being a driving force for malignant progression in ΔS6 and ΔS6:ΔPTEN livers comes from studies showing that chronic activation of mTOR is sufficient to promote HCC in mice [105,106] and accelerates liver tumorigenesis in conjunction with hyperactivated PI3K/Akt [107]. Moreover, studies using Rps6^P-/-^ mice have shown that Rps6 phosphorylation facilitates Kras-dependent pancreatic cancer initiation [108] and Akt-driven pancreatic β-cell tumorigenesis [109], suggesting that Rps6 phosphorylation may also contribute to tumor development in ΔS6 livers, together with, or independent of the broader pro-oncogenic mTOR network.

A second feature of ΔS6 livers that we consider relevant for tumor development is the inflammatory environment as indicated by the many cytokines and DAMPs that were upregulated at the mRNA level. This signature implicated NF-κB and MAPK, both of which have previously been reported as being induced in several ribosomopathies [110–113] as mediators of inflammation, innate immunity activation and senescence. Inflammation is an established driver of many cancers including those of the liver [114–118] and NF-κB has been shown to promote HCC in the setting of cholestatic-induced liver injury akin to that seen in ΔS6 livers [119] as well as in the setting of Rpl22-deficiency via induction of the pluripotency factor Lin28b [120]. NF-κB and MAPK can also indirectly facilitate tumor growth by inducing senescence and the senescence-associated secretory phenotype or SASP [121–123] which stimulates the production of mitogenic growth factors within the microenvironment [124,125].

The paradox of the hypo- to hyper-proliferative switch in ribosomopathies poses a problem for physicians in that it remains to be seen if therapeutic regimens that are used to boost cell proliferation or prevent death as a means to manage hypoplastic disease in young patients will have unintended consequences later in life. Indeed, since L-Leucine was first shown to be effective at mitigating ribosomopathy-associated growth defects in animal models [126,127], this powerful mTOR activator has been considered as a possible therapeutic treatment for several ribosomopathies [128–130] even though inhibition of mTOR has also been shown to alleviate ribosomopathy-associated proteotoxic stress [131]. Analogous to the idea of activating mTOR to boost cell growth in ribosomopathies, our finding that a very modest overexpression of c-Myc ameliorated many of the deleterious effects of Rps6-insufficiency in the liver suggests that c-Myc augmentation may be yet another option for improving ribosomopathy-associated cytopenias. However, given that the same level of c-Myc that provided hepatoprotection to ΔS6 livers during the early phase of disease also accelerated tumor development in aged ΔS6:c-Myc mice, further work is needed to determine the extent to which c-Myc’s hepatoprotective or tumor-promoting activities are influenced by other factors that are impacted by Rps6 insufficiency such as mTOR activation [132] or inflammation [133]. Careful monitoring of any regimens that rely on the activation of powerful growth stimulators such as mTOR or c-Myc will likely be necessary to ensure that any therapeutic benefit is tilted in favor of “physiological” growth promotion rather than oncogenic stimulation during the different phases of a ribosomopathy.

The key question regarding stabilization of p53 in ΔS6 livers is to what extent it drives hepatobiliary disease. Although p53 has occupied center stage as a major effector of the ribosomal stress response [7,134], accumulating evidence indicates that that p53-dependent and -independent mechanisms contribute to ribosomopathy-associated pathogenesis [17,21–23,135–142]. Using a gain-of-function approach to investigate the role of p53 in ΔS6-associated liver disease, we found that hepatoblast-specific stabilization of p53^QS^ generally mimicked the cholangiopathy in ΔS6 livers but was unable to trigger the rapid or robust hepatocyte death that drove neonatal ΔS6 livers towards near-catastrophic failure. Indeed hepatocytes tolerated expression of p53^QS^ much better than loss of Rps6 and survived for several months longer than their Rps6-deficient counterparts. While this contrasts with a study showing that p53^QS^ precisely phenocopies pigmentation defects caused by ribosomal protein mutations *in vivo* [64], studies continue to reveal increasing complexity in the mechanisms by which p53 influences cell fate at the level of autocrine and paracrine signaling [143–148] meaning that the ability of p53 activation to mimic phenotypes induced by Rp-insufficiencies is likely to be highly cell- and context-dependent. An example of this is seen in the liver whereby hepatocyte-specific depletion of p53’s negative regulator Mdm2 induces widespread hepatocyte loss via apoptosis, necrosis and senescence [149], while biliary-specific inactivation of Mdm2 induces hepatocyte senescence via a paracrine mechanism involving hepatocyte-specific induction of p21 and TGF-β [150]. This not only demonstrates how different cell types within a single tissue can differ in their response to p53 stabilization but shows how the liver constantly senses the p53 status of hepatocytes and biliary cells to relay autocrine or paracrine signals to the cell-cycle or apoptotic machineries as a means to regulate hepatic homeostasis. While there is a clear distinction between the aforementioned Mdm2-deletion studies that describe phenotypes stemming from stabilization of wild-type p53 in either hepatocytes or biliary cells of the adult liver, and this study in which a hypomorphic p53 mutant is targeted to hepatoblasts of the embryonic liver, all provide valuable insight into how inappropriate activation of p53 might drive hepatobiliary disease while also indicating what arms of the p53 response might be involved. Indeed, the very different fates of Mdm2-deficient hepatocytes which undergo rapid death [149] and p53^QS^-expressing hepatocytes which do not, indicate that functions of p53 that are compromised in the p53^QS^ mutant are important for determining whether hepatocytes are driven towards arrest/senescence or die. Moreover, even though p53^QS^ is compromised for transcriptional activation of *p21* and *noxa* [62,63,151], both were upregulated in p53^QS^ livers suggesting that certain aspects of the phenotype are p53-independent. Future studies involving the selective targeting of hypomorphic p53 mutants to either hepatocytes or cholangiocytes will not only be important for determining how cell-autonomous and/or paracrine mechanisms related to p53 signaling drive hepatic dysfunction, but should also help to establish which arms of the p53 network are important for effecting arrest, senescence or death in each cell type, information that will undoubtedly be useful for furthering our understanding of the pathogenic underpinnings of liver disease.

Our results showing that loss of p53 not only failed to provide any major benefit to ΔS6 livers, but exacerbated certain aspects of liver dysfunction contrasts with reports of p53 deletion rescuing other phenotypes driven by *Rps6*-haploinsufficiency in mice [45,46] or Cirhin1A-deficiency in zebrafish [83]. While we acknowledge that p53 likely mediates some aspect(s) of ΔS6-associated liver dysfunction, we propose that rather than it being the sole driver, it is but one arm of a broader stress response to Rps6-insufficiency such that the impact of p53 ablation in the liver is masked. Indeed, although loss of p53 extends the viability of *Rps6*^*wt/del*^ embryos, it fails to correct the proliferative defect that is seen in the liver of these embryos [45] suggesting that the liver may favor p53-independent mechanisms to limit cell proliferation or induce death when RiBi is compromised. Furthermore, in reconciling why loss of p53 rescues hepatic Cirhin1A- but not Rps6-deficiency, we note that hepatic dysfunction in Cirhin1A-deficient livers was confined to the biliary compartment even though Cirhin1A is reported to be expressed in cholangiocytes and hepatocytes suggesting that hepatocytes do not require Cirhin1A for survival. Moreover, rRNA processing was disrupted in Rps6- but not Cirhin1A-deficient livers, a difference that can perhaps be reconciled by the fact that processing of 30S pre-RNA requires Rps6 phosphorylation [152] which is likely compromised in neonatal ΔS6 livers. Notwithstanding the loss of RiBi-related and phosphorylation-dependent signaling functions of Rps6 [44,103], what effectors or mechanisms could be acting in concert with p53 to mediate ΔS6-associated liver disease? On the basis of our results showing that overexpression of c-Myc blocked hepatocyte death in ΔS6 livers and that down regulation of c-Myc contributes to hypoplastic disease in other models of RiBi dysfunction [59], downregulation of c-Myc may be a possible mediator of the neonatal liver hypoplasia. Others include NF-κB which was not only deemed activated in ΔS6 livers but which has emerged as a hub for orchestrating the nucleolar stress response [153], and proteotoxic stress which promotes apoptosis as a consequence of overwhelming the protein degradation machinery [131,154].

Lastly, we propose that intrinsic cell- and tissue-specific functions of p53 unrelated to ribosomal stress have the potential to influence the extent to which p53-nullizygosity can rescue abnormalities caused by dysfunctional RiBi. For instance, although livers of young p53-/- mice appear normal with respect to bile duct development, liver mass and lobular architecture, hepatocytes become increasingly dysplastic as mice age. Moreover, as reported over 20 years ago [155], almost all of the p53-null livers that we analyzed contained immature hepatoblast-like cells suggesting that p53 may not only be required for restricting their unscheduled expansion in the adult liver, but may also regulate their differentiation. Other studies have also identified a previously unappreciated hepatoprotective role for p53 by showing that p53-null livers are more, not less susceptible to drug- or metabolic-induced injury [156–158]. Given that the liver’s ability to respond to acute or chronic injury depends on the successful execution of a regenerative program that can reconstitute functional hepatocytes and biliary cells in a timely manner, rendering ΔS6 livers null for p53 could hamper their ability to fully repair the damage caused by loss of Rps6, providing a plausible explanation as to why its loss not only fails to rescue the ΔS6 phenotype, but as we saw, exacerbated it.

Our results suggest that future investigations into the role of RiBi in mammalian hepatobiliary development and disease are warranted. Although NAIC currently stands as the only ribosomopathy known to affect the liver, a subset of SDS patients present with persistent cholestasis, biliary hamartomas or other indicators of hepatic disease [159–161]. Moreover, Sbds-deficiency not only induces atrophy of the pancreas, but also of the liver [139], suggesting that the liver may be more vulnerable in some of the ribosomopathies than previously appreciated. A complete picture of the requirement for a fully functioning RiBi machinery in mammalian hepatobiliary development and the consequences of its dysfunction for liver disease will require a comprehensive analysis of RiBi gene expression in cholangiocytes and hepatocytes coupled with conditional liver-specific targeting strategies that can drive expression down to a sufficiently low level in one or both hepatic compartments *in vivo* or in organoids. Existing rp-haploinsufficient mutants may also be useful for determining if livers are more susceptible to drug-induced or metabolic stress; information that could help determine whether certain dietary or life-style factors or pharmacological interventions used in the clinical management of ribosomopathy patients increase their risk of developing liver disease. Finally, although p53 has traditionally been viewed through the prism of tumor suppression, the fascinating new area of research that has begun to implicate aberrant activation of p53 in a number of developmental syndromes [143,162,163] raises the tantalizing question of whether defective RiBi or unscheduled p53 activation accounts for any cholangiopathies that are currently without a known etiology. The myriad of genes involved in the minting of ribosomes, together with the ease of generating CRISPR-induced mutations, and advances in ribosome profiling and quantifying protein synthesis *in vivo* lends itself to a virtually limitless number of experiments with the potential to yield new insight into these fascinating diseases.

## Materials and methods

### Ethics statement

All experiments were conducted in accordance with the National Institutes of Health Guide for the Care and Use of Laboratory Animals and with approval of the University of Texas Southwestern Medical Center Institutional Animal Care and Use Committee (IACUC).

### Mouse strains

Conditional *Rps6*^*lox/lox*^ mice, *Albumin-c-Myc* mice and conditional *Trp53*^LSL-Q25S26^ mice were generously provided by George Thomas (Catalan Institute of Oncology, Barcelona, Spain), Eric Sandgren (University of Wisconsin, Madison) and Laura Attardi (Stanford University) respectively. *Albumin-Cre* (*Speer6-ps1*^*Tg(Alb-cre)21Mgn*^, Stock # 003574), *Pten*^*lox/lox*^ (*Pten*^*tm1/Hwu*^, Stock # 004597) and *p53-/-* (*Trp53*^tm1Tyj^, Stock # 002101) mice were purchased from The Jackson Laboratory (Bar Harbor, ME). *ApoE-rtTA*:*TRE2-Cre* transgenic mice were generated as described in S8 Fig. Mixed hybrid strain (C57Bl6/129/SJL) male and female mice were used for all studies unless otherwise stated. Mice were housed in a conventional colony under standard 12 hour light/dark cycles and allowed access to water and chow *ad libitum*.

### Genotyping of mice and analysis and quantitation of recombination

Oligonucleotide sequences and conditions for PCR-genotyping of mouse strains and assessment of Cre-mediated recombination of the loxp-flanked *Rps6* and *Pten* alleles are shown in S5 Table. Ethidium stained gels showing recombination were imaged using Image J software [164]. To determine the % Rps6 recombination in livers, the intensity signal values for the imaged *Rps6*^*lox*^ (upper) band and the recombined ΔS6^*del*^ (lower) band were added together to produce an arbitrary value of 100%. The extent of recombination was determined by representing the signal value of the ΔS6^*del*^ (lower) band as a percentage of the total.

### Analysis of liver function

Peripheral blood was collected from the retro-orbital sinus using a heparinized glass capillary tube. Biochemical analysis of liver function (ALT, AST, T-Bil and Alk-Phos) was performed by UT Southwestern Medical Center’s Metabolic Phenotyping Core using a Vitros 250 bioanalyzer. Plasma was isolated from ≥3 individual mice/group.

### Histopathology and immunohistochemistry (IHC)

Small pieces (~1cm x 1cm) of freshly harvested liver were immersion fixed in 10% neutral-buffered formalin for 24–48 hours and processed to paraffin for sectioning at 5 microns for routine H & E staining or IHC as previously described [165]. Primary and secondary antibodies and reagents and conditions used for IHC are listed in S6 Table. Antigen retrieval (Retrievagen A pH 6.0, BD Pharmingen) was performed in a microwave at 95°C for 10 minutes for phospho-S6, total S6 and pan-CK, 20 minutes for p53 and 50 minutes for c-Myc IHC. All primary antibody incubations were performed overnight at 4°C except for c-Myc which was performed for 48 hours at 4°C. All secondary antibody incubations were performed at room temperature for 1–2 hours and slides were incubated with streptavidin horseradish peroxidase (HRP) conjugate at room temperature for 20–60 minutes. 3-Amino-9-Ethylcarbazole (AEC) chromagen was prepared as recommended by the manufacturer. After color development, sections were either immediately cover-slipped (no counterstain) or counterstained with Hematoxylin QS.

### Analysis of apoptosis

Liver cell apoptosis was determined using the DeadEnd Colorimetric TUNEL System (Promega, Catalog #G7360) applied to de-paraffinized liver sections as recommended by the manufacturer. For quantitation of the number of TUNEL-positive hepatocytes, at least 30-fields per liver section were counted. Livers from at least three mice per genotype and age were evaluated.

### Bile duct and biliary epithelial cell (BEC) quantitation

To quantify the number of patent bile ducts per portal vein (bds/PV) in H&E stained sections, 10 portal veins (sampled from three or more animals for each genotype and age) were scored. To quantify the number of Sox9+ biliary epithelial cells (BECs), livers from three mutant animals and three controls were examined and positive cells scored from at least ten portal tracts per animal. Portal veins were identified by the presence of five or more biliary cells in the perivascular region and associated bile ducts were counted. *P* values were calculated by Student’s *t*-test.

### RNA isolation, Northern blotting and probe labeling

Total liver RNA was prepared using RNA STAT 60 (Fisher Scientific, Catalog # CS-111) as previously described [165]. For Northern blotting, 12μg of total RNA was denatured at 50°C with glyoxal before separating on a 1.5% agarose-formaldehyde gel using a vertical gel apparatus. Fractionated RNA was transferred to Hybond-N^+^ membranes (GE Healthcare) overnight and hybridized to [α-^32^P]dCTP radiolabeled cDNA probes prepared using a Random Primed DNA labeling kit (Sigma, 11004760001). The following day membranes were washed and exposed to Blue Lite Film (ISC BioExpress, F-9024) film with intensifying screens at -80°C for 8–24 hours, after which they were stripped and re-probed with a radiolabeled oligonucleotide probe specific for 18S rRNA (5′ GCCGTGCGTACTTAGACATGCATG 3′ corresponding to nucleotides 50–73 of the rat ribosomal RNA gene) end-labeled with [γ-^32^P]ATP using T4 polynucleotide kinase (Fisher, Pittsburgh, PA) or a cyclophilin A-specific cDNA probe for assessment of RNA loading. The 840bp cDNA probe for *Rps6* was obtained by NotI/SalI digestion of plasmid pCMV-Sport6 containing mouse Rps6 cDNA (ATCC Product # 10538869). The 4.8kb cDNA probe for mouse *Myc* was isolated and purified by digesting plasmid pSV-c-Myc-1 (ATCC # 41029) with BamHI and XbaI. The 721bp mouse *Ppia*/cyclophilin A-specific probe was obtained from Ambion (AM 7375).

### mRNA expression analysis by semi-quantitative (sq) RT-PCR

1μl of cDNA synthesized from DNase 1-treated total liver RNA was used as a template for sq PCR amplification using TAq Polymerase (Qiagen) and primers and conditions listed in S7 Table. Confirmation of differential expression was obtained by running half of the PCR product on an agarose gel and quantifying relative band intensities using Image J software.

### Analysis of rRNA processing

Pre-rRNA processing was analyzed by Northern blotting using 2 synthetic radiolabeled oligonucleotide probes: ITS1 (5′ CTCTCACCTCACTCCAGACACCTCGCTCCA 3′), which is homologous to nucleotides 5977–6006 and ITS2 (5′ ACCCACCGCAGCGGGTGACGCGATTGATCG 3′) which is homologous to nucleotides 7036–7065 of the mouse 45S pre-ribosomal RNA (NCBI Ref. Seq. NR_046233.2). Blots were stripped and re-probed with a radiolabeled oligonucleotide probe specific for 18S rRNA.

### Preparation of proteins and immunoblotting

Preparation of total protein lysates was performed as previously described [47]. Nuclear and cytoplasmic proteins were fractionated as follows; ~ 250mg of freshly isolated or previously frozen (-80°C) liver was homogenized in 2.5 mls of ice-cold homogenization buffer (20mM Tris-Cl (pH7.4), 2mM MgCl_2_, 0.25M sucrose, 10mM EDTA, 10mM EGTA, 0.2mM Na_3_VO_4_, 1mM NaO_7_P_2_.10H_2_0, 10mM NaF containing protease inhibitors (cOmplete Protease Inhibitor cocktail (Millipore Sigma, Catalog #11697498001), 0.4μM Microcystin LR (Millpore Sigma, Catalog # 475815), 0.2μM Okadaic Acid (Enzo Life Sciences, Catalog # ALX-350-003-C100) and 30μg/ml PMSF Millipore Sigma, Catalog # 10837091001) using a Dounce tissue grinder and centrifuged at 1000 x g for 5 mins at 4°C. The supernatant and pellet were then processed as follows. The supernatant was removed and re-centrifuged at 100,000 x g for 1 hour at 4°C, after which the pellet was discarded and the supernatant (cytosolic fraction) aliquoted, snap frozen in liquid N_2_ and stored at -80°C. The pellet generated by the 1000 x g centrifugation was washed and gently resuspended in 3 mls of homogenization buffer and re-centrifuged at 1000 x g for 5 minutes at 4°C. After discarding the supernatant, the pellet was gently re-suspended in 200μl of ice-cold nuclear extract buffer (20mM HEPES (free acid) NaOH (pH7.6), 2.5% glycerol, 0.42M NaCl, 1.5mM MgCl_2_, 1mM EDTA, 1mM EGTA, 0.2mM Na_3_VO_4_, 1mM NaO_7_P_2_.10H_2_0, 10mM NaF containing protease inhibitors (cOmplete Roche Protease Inhibitor Cocktail), 0.5μM Microcystin LR, 0.25μM Okadaic acid and 30mg/ml PMSF) and incubated at 4°C with gentle rocking for 45 minutes. Following centrifugation at 100,000 x g for 30 minutes at 4°C, the supernatant containing nuclear proteins was removed and aliquoted, snap-frozen in liquid N_2_ and stored at -80°C. Immunoblotting was conducted as previously described [165]. 100 micrograms of protein (or 50 micrograms in the case of abundant housekeeping proteins such as GADPH) was loaded per lane. Antibodies used for immunoblotting are listed in S8 Table.

### Imaging and quantitation

Ethidium bromide-stained gels, Northern blot and immunoblot films were imaged using Image J software. mRNA and protein levels were quantified by determining fold-changes after normalizing signal intensities to cyclophilin A for sq-PCR cDNA analysis, 18S rRNA or cyclophilin A for Northern Blot analysis and GAPDH for immunoblot analysis.

### Rapamycin treatment

5–6 week old WT and ΔS6 mice received 6 intrapertioneal (i.p) injections of rapamycin (Calbiochem, Catalog # 553210) (2.5mg/kg body weight) or vehicle (DMSO/saline) on day 1, 3, 5, 7, 10 and 12. On day 13, mice were sacrificed and livers harvested for histology, IHC processing and protein lysate preparation. 4 mice (2 WT and 2 ΔS6 mice) received vehicle and 6 mice (3 WT and 3 ΔS6) mice received rapamycin.

### Microarray analysis and bioinformatics

Microarray analysis of WT and ΔS6 livers was performed using cDNA generated from pooled total liver RNA isolated from 3 individual WT and 3 individual ΔS6 male mice at 5 weeks of age. cDNA was hybridized to Affymetrix Mouse 430 v2.0 GeneChips and data normalized using the RMA procedure [166], followed by quantile normalization [167]. 235 differentially expressed genes (up or downregulated ≥8 fold) were identified in ΔS6 livers. Gene association network enrichment was performed using Ingenuity Pathway Analysis (IPA) (Ingenuity Systems, Inc. Redwood City, CA). The top 4 networks activated in ΔS6 liver (Cdkn1a, Jun/Spp1, NF-κB and MAPK; S7 Fig) were obtained from the 235 differentially expressed genes with an enrichment score of 38 to 36. Colored shapes (red: over-expression, green: under-expression) represent genes within the differential gene list while open shapes represent genes are not on the significant gene list but are associated with the network. Arrows represent positive regulation of gene expression, with solid arrows indicating direct regulation, and broken arrows indicating indirect regulation.

## Supporting information

S1 Fig Deletion of hepatic Rps6 stunts post-natal growth and causes neonatal hepatic hypoplasia. (A-D) Graphs of post-natal body weights, liver weights and %Liver/Body weights (%L/BWs) in WT and ΔS6 mice. In graphs (A and B) body weight values from post-natal day 1–15 (P1-P15) include male and female mice. From P21-24 onwards, graph A) represents values for males only while graph (B) represents values for females only. Gray boxes indicate ages at which body weight in ΔS6 mice differs significantly from WT. For males (A), *P* values range from .041 at P33-37 to .004 at P25-28. For females (B), *P* values range from .036 at P33-37 to < .0001 at P25-28. At P15, *P* = .0003; 2-tailed unpaired Student’s *t* test. (C and D) Graphs of liver weight (C) and %L/BW (D) in male WT and ΔS6 mice from P7-8 to P39. Gray boxes indicate ages at which liver weight and %L/BW values in ΔS6 mice differ significantly from WT. For liver weights (C), *P* values range from .016 at P21-25 to < .0001 at P15. For %L/BWs (D), *P* values range from .029 at P21-25 to .0028 at P27-32; 2- tailed unpaired Student’s *t*-test.
(TIF)

S2 Fig Incomplete Albumin-Cre-mediated deletion of *Rps6* results in mosaicism for RPS6 expression across the neonatal liver. (A) Ethidium stained agarose gel showing PCR analysis of recombination of the ΔS6^*del*^ allele (lower band) in control (WT) (*Rps6*^*lox/lox*^) (lane 1) and ΔS6 (*S6*^*lox/lox*^:*Alb-Cre*) livers (lanes 2–11) at different ages from P1 to P37. Recombination increases to a maximal level of ~50–60% by P37. (B) Graph showing quantitation of the gel shown in A) representing the % of the recombined ΔS6^*del*^ allele (lower band) relative to the total signal in each lane (sum of the recombined (lower) ΔS6^*del*^ band plus the non-recombined S6^lox^ allele (upper band)). (C) Northern blotting of 12 μg of total liver RNA from WT (lanes 1 and 2) and ΔS6 livers (lanes 3–6) with a p^32^-radiolabeled Rps6-specific probe. After stripping, the blot was incubated with a p^32^-radiolabeled 18S rRNA probe. (D) Graph showing quantitation of the Northern blot shown in (C) demonstrating that *Rps6* mRNA levels are decreased by 50–60% in ΔS6 livers relative to WT. (E) IHC of a P15 WT liver (a) and 2 individual ΔS6 livers (b and c) with an antibody that recognizes total RPS6 protein. RPS6 is expressed across the lobule in WT liver at P15 (a), but becomes more restricted to periportal zone 1 as the liver matures. Residual RPS6 protein is visible in the ΔS6 livers indicating that Alb-Cre-mediated deletion is incomplete and regional across individual livers and varies between mice. A bile infarct (*) is visible in the ΔS6 liver in (b). Original magnifications, all x 62.5. Scale bars; 100μ. AEC chromagen (red), hematoxylin counterstain (blue).
(TIF)

S3 Fig Neonatal ΔS6 livers display hepatic dysfunction that only partially resolves with age. Liver function tests (LFTs) performed on heparin-treated plasma isolated from ≥ 4 control (*Rps6*^*lox/lox*^) and 4 ΔS6 (*Rps6*^*lox/lox*^:*Alb-Cre*) mice at P32-36 and P57-60. While markers of hepatocellular (ALT and AST) and biliary (Alk-Phos and T-Bil) dysfunction are all markedly elevated in ΔS6 mice at P32-P36, hepatocellular dysfunction persists, while biliary function improves as mice age (ALT, alanine aminotransferase; AST, aspartate aminotransferase; Alk-Phos, alkaline phosphatase; T-Bil, total bilirubin). ****P < .0001, ***P.<0005, **P < .*005*, *P < .05. (2-tailed unpaired Student’s *t*-test).
(TIF)

S4 Fig IHC profiling of ΔS6 livers identifies immature hepatocytes and HPC/oval cells as constituents of regenerating nodules and the ductular reaction (dr) respectively. (A-D) Photomicrographs of H & E stained WT and ΔS6 livers between P35-P42 at low (A, B) and high (C, D) magnification showing regenerative nodules (n), (bounded by dotted lines) and the ductular reaction (dr) in ΔS6 livers. E-X) Photomicrographs of P35-P42 WT and ΔS6 livers after performing IHC for PCNA (e, f) and variety of markers known to be expressed in a cell-type or location-dependent manner within the liver. IHC profiling confirms that nodules are composed of highly proliferative AFP^+^, HNF4B^+^ immature hepatocytes (F, H), in contrast to cells within the dr, which are HNF1B^+^, SOX9^+^, pan-CK^+^, TROP2^+^ and EPCAM^+^ (L, N, P, R, T) consistent with an oval cell/HPC identity. Retention of β-catenin/CTNNB1 at the membrane of nodular hepatocytes (V) and the absence of staining of the hepatic β-catenin target GLUL (glutamine synthetase) in nodules (X) suggests that wnt signaling is not driving nodular growth in ΔS6 livers. AEC Chromagen (red), hematoxylin counterstain (blue). Original magnifications: A, B (x 75); C, D, O, P (x 112); E, F, W, X (x 62.5); G-N and Q-T (x 125); U, V (x 250). Scale bar; 50μ.
(TIF)

S5 Fig Analysis of total and phosphorylated RPS6 protein in WT liver and regenerating ΔS6 liver reveals differential mTOR activity in hepatocytes and biliary cells and that RPS6-expressing cells participate in regeneration in ΔS6 livers. (A) IHC of WT (a-d) and ΔS6 liver (e, f) with an antibody that recognizes total RPS6 irrespective of its phosphorylation status (a, c, e) and one that recognizes RPS6 only when phosphorylated on the mTOR-dependent Ser235/236 sites (b, d, f). In WT liver, although RPS6 is highly expressed in bile ducts (bd, arrowhead) and throughout the parenchyma in a decreasing periportal-pericentral gradient (a, c), phosphorylated-RPS6^(Ser235/6)^ is largely restricted to periportal hepatocytes and is absent from bile ducts (b, d). In ΔS6 liver, immature hepatocytes in nodules (n) and dr cells both express abundant RPS6 (e), yet it is only phosphorylated in nodular hepatocytes (f). Original magnifications: a, b, e, and f (x 112); c and d (x 225). Scales bars, 50μ. (B) Western blot of proteins isolated from WT (lanes 1 and 2) and ΔS6 livers (lanes 3 and 4) showing that mTOR signaling to 4E-BP1 and RPS6 is hyper-activated in ΔS6 livers, while Akt signaling is not. The absence of a visible p-RPS6^Ser235/6^ signal in WT livers reflects a level of Ser235/6 phosphorylation in regenerating ΔS6 livers (lanes 3 and 4) that is much higher than in WT liver (lanes 1 and 2) necessitating a short exposure that is not sufficient to visualize the p-RPS6 signal in WT liver. Phosphorylation of the mTOR target 4E-BP1 is indicated by an increase in the abundance of the higher molecular weight (γ) form of the protein in ΔS6 livers. (C and D). IHC (C) and Western Blotting (D) of livers with the total (a-d) and phospho-RPS6^(Ser235/6)^ antibodies (e, f) in vehicle (DMSO) (a, c, e) and rapamycin-treated (b, d, f) ΔS6 mice showing that RPS6 phosphorylation in nodules of ΔS6 livers is mTOR-dependent. The residual signal in cells of the dr in rapamycin-treated livers reflects the presence of RPS6 protein that is not phosphorylated on Ser235/6 (b, d). AEC chromagen (red), hematoxylin counterstain (blue). Original magnifications: a, b, e and f (x 62.5); c and d (x 125). PV, portal vein; CV, central vein. Scale bars, 50μ.
(TIF)

S6 Fig *Rps6*-deficiency induces p53 and disrupts rRNA processing. (A) Immunoblot of total protein lysates prepared from 2 WT (lanes 1 and 2) and 2 ΔS6 livers (lanes 3 and 4), p53-null Saos 2 cells (lane 5) and A431 cells that express high levels of mutant p53 (lane 6) with a p53-specific antibody and a GAPDH-specific antibody for load control. Abundant p53 protein is visible in ΔS6 livers (upper band, lanes 3 and 4) and A431 cells (lane 6). A faster migrating non-specific band (*) is present in all samples. (B) Photomicrographs of IHC of liver sections from an adult SV40 T-antigen (SV40-TAg) transgenic mouse (a) and from WT (b, c) and ΔS6 livers (d-g) at E17 and P7 incubated with a p53-specific antibody. p53 is stabilized in the nuclei of hepatocytes expressing SV40-TAg (a) and in a subset of hepatoblasts in ΔS6 livers in response to depletion of Rps6 (d-g). AEC chromogen (orange); no counterstain. Original magnifications; a, d, e (x 125; scale bars, 50μ); b, c, f, g (x 250; scale bars, 25μ). (C) Left: Northern blot of liver RNA from WT and ΔS6 mice hybridized to a radiolabeled ITS1 probe homologous to nucleotides 5977–6006 of the mouse 45S pre-ribosomal RNA (left) showing that *Rps6* deficiency causes an rRNA processing defect that results in the accumulation of 30S rRNA and a decrease in the amount of 21S rRNA, both of which are precursors of the mature 18S rRNA. Right: Northern blot analysis of the same RNAs hybridized to a radiolabeled ITS2 probe homologous to nucleotides 7026–7065 of the mouse 45S pre-ribosomal RNA showing decreased abundance of 17S rRNA. Graphs were generated using Image J to estimate fold-changes in the abundance of rRNA species in ΔS6 livers relative to WT (assigned an arbitrary value of 1).
(TIF)

S7 Fig Hepatic *Rps6*-deficiency alters the expression of a large number of mRNAs. (A) Scatter plot of the microarray data. (B) Ingenuity pathway analysis (IPA) of the microarray data showing that loss of Rps6 activates hepatic gene expression programs associated with cell cycle arrest/senescence (cdkn1a, MAPK), regeneration (jun/spp1) and inflammation/activation of innate immunity (NF-κB).
(TIF)

S8 Fig Schematic of ApoE-rtTA and TRE-Cre transgenes used to generate ApoE-rtTA-TRE-Cre transgenic mice. A 764bp fragment encoding the reverse tetracycline transactivator rtTA-M2 cDNA was subcloned into the Mun I-Cla I sites of plasmid pLIV11 containing the promoter, intron 1 and hepatic control region (HCR) of the human ApoE gene [50,168]. The resulting 10.5kb ApoE-rtTA transgene was excised from unwanted vector sequences by digestion with Sal I and Spe I. The TRE-Cre transgene was generated by subcloning a 1.1kb fragment encoding the Cre recombinase into the BamHI-Xba 1 sites of plasmid TRE2 containing the reverse tetracycline response element and minimal CMV promoter. The 2.8kb transgene was excised from the plasmid by digestion with Xho I and Sap I. Both transgenes were co-injected into one-cell embryos to generate *ApoE-rtTA-TRE2-Cre* bigenic mice in which liver-specific expression of Cre is induced in hepatocytes following doxycycline administration.
(TIF)

S9 Fig Analysis of recombination and mTOR and PI3K-dependent signaling in ΔS6ΔPTEN livers. (A) Ethidium stained agarose gel showing that the efficiency of recombination of either the ΔS6^*del*^ or ΔPTEN alleles is unaffected by the presence of the other in livers of mice doubly deficient for Rps6 and PTEN. (B) Western blots of proteins prepared from livers of 6–11 month old WT (lanes 1–3), ΔS6 (lanes 4–6), ΔPTEN (lanes 7–9) and ΔS6ΔPTEN (lanes 10–12) mice with antibodies specific for total and phospho-specific forms of RPS6 and AKT, PTEN and GAPDH (for load control). While RPS6, but not AKT is hyperphosphorylated in ΔS6 livers and AKT, but not RPS6, is hyperphosphorylated in ΔPTEN livers, both are hyperphosphorylated in ΔS6ΔPTEN livers indicating co-activation of both mTOR and PI3K. (C) Graphs showing Image J quantitation of phospho-RPS6^(Ser235/6)^, phospho-AKT^(Ser473)^ and PTEN protein levels from western blots shown in (B).
(TIF)

S10 Fig HPC/oval cells are the source of elevated c-Myc in ΔS6 livers. (A) Northern blot of total liver RNA (12μg/lane) from WT (lanes 1 and 2) and ΔS6 mice (lanes 3–6) and an Albumin-c-Myc transgenic mouse. The blot was first incubated with a p^32^-radiolabeled c-Myc-specific cDNA probe, followed by stripping and re-probing with an p^32^-radiolabeled cyclophilin A-specific cDNA probe. Note that the size of the endogenous *Myc* transcript differs from that transcribed from the Albumin-c-Myc transgene. (B) Graph of quantitation of relative *Myc* mRNA levels in Northern Blot shown in A). With *Myc* expression in WT liver set at an arbitrary value of 1, *Myc* mRNA is elevated ~4-5-fold in ΔS6 livers, slightly less than that expressed in livers of Albumin-c-Myc transgenic mice (~7-8-fold increase). (C) IHC with a c-Myc-specific antibody showing that MYC protein is undetectable in normal hepatocytes and biliary cells (arrowheads) in WT liver (a, b), but is abundantly expressed in HPCs within the ductular reaction (dr), but not regenerating nodules (n) of ΔS6 livers (c, d). Original magnifications; a, c x125; scale bars 50μ; b, d x312; scale bars, 25μ. (D) IHC of ΔS6 livers showing that MYC and pan-cytokeratin (pan-CK) are specifically expressed in and co-localize to HPCs within the dr. Original magnifications; a, b x 62.5; scale bars, 50μ. For all IHC, AEC Chromagen (red) with hematoxylin counterstain (blue).
(TIF)

S11 Fig Overexpression of c-Myc preserves hepatocyte viability, reduces injury and suppresses the expression of mRNAs associated with induction of NF-κB/innate immunity signaling in ΔS6 livers. (A) Ethidium-stained agarose gels showing PCR genotyping of mice (top 3 panels) and recombination of the ΔS6^*del*^ allele (lower panel) in livers of WT, ΔS6, Alb-c-Myc and ΔS6:c-Myc mice. By preserving hepatocyte viability, c-Myc overexpression inadvertently increases recombination of the ΔS6^*del*^ allele from ~50% in ΔS6 mice (lanes 3 and 4) to ~80% in ΔS6:c-Myc mice (lanes 7 and 8). (B) Ethidium-stained agarose gel and graph of quantitation showing that the efficiency of recombination in ΔS6 livers (lanes 1–3) is augmented by c-Myc overexpression (ΔS6:Myc livers, lanes 4–6) but not by loss of PTEN (ΔS6:ΔPTEN livers, lanes 7–10). (C) Northern Blot of 12μg of total RNA isolated from WT, ΔS6, Alb-c-Myc and ΔS6:c-Myc livers with p^32^-radiolabeled probes specific for Rps6 (top panel), c-Myc (second panel), 18S rRNA (third panel) and Ppia/cyclophilin A (bottom panel). Note that *Rps6* mRNA levels are lower in ΔS6:Myc livers (lanes 7 and 8) than in ΔS6 livers (lanes 3 and 4) reflecting the higher number of Rps6-negative hepatocytes in ΔS6:c-Myc livers as a consequence of c-Myc preserving hepatocyte viability. Note that the lower level of *Rps6* in ΔS6:c-Myc livers has also become limiting for 18S rRNA production. Quantitation of relative mRNA levels for *Rps6* and *18S* rRNA are shown in the graph below the blot. (D) Semi-quantitative (sq)-PCR of cDNA prepared from WT, ΔS6, Alb-c-Myc and ΔS6:c-Myc liver. c-Myc normalizes the expression of a specific subset of mRNAs related to activation of NF-κB and innate immunity and HPC-activation induced in response to loss of S6, but not classical p53 targets or imprinted genes. Results reflect sq-PCR conducted on cDNA synthesized from total RNA isolated from the livers of ≥3 individual mice of each genotype. (HPC, hepatic progenitor cell associated genes; imp, imprinted genes).
(TIF)

S12 Fig Overexpression of c-Myc rescues the neonatal growth defect and liver hypoplasia in ΔS6 mice. (A) Graphs of body weights of male (top 2 graphs) and female (bottom 2 graphs) WT, ΔS6, Alb-c-Myc (c-Myc) and ΔS6:c-Myc mice at different ages showing that body weights of ΔS6:c-Myc mice are indistinguishable from WT mice between ~3 and 5.5 weeks of age, the age at which ΔS6 mice show the greatest degree of growth retardation. (B) Graphs of % Liver/body weights in males and females showing that overexpression of c-Myc in ΔS6 livers also rescues the liver hypoplasia associated with Rps6-insufficiency. Significance was calculated using the 2-tailed unpaired Student’s *t*-test. Additional body weight analysis for one extra time point for both males and females is shown in accompanying S4 Table.
(TIF)

S13 Fig Overexpression of c-Myc in ΔS6 livers does not prevent overgrowth or tumor development. (A) Graph of %L/BWs of WT, ΔS6, Alb-c-Myc and ΔS6:c-Myc mice at ≥ 6 months of age showing that livers of ΔS6, Alb-c-Myc and ΔS6:c-Myc mice are all predisposed to overgrow as they age. Mean %L/BWs (+/- SEM) (number of mice): WT (4.5 +/0.15) (n = 29); ΔS6 (7.8 +/- 0.59) (n = 34); Alb-c-Myc (9.1 (+/- 0.92) (n = 56); ΔS6:c-Myc (17.0 +/- 2.19) (n = 19). The statistical difference in %L/BWs between ΔS6:Myc and ΔS6 mice; *P*< .0001, and between ΔS6:c-Myc mice and Alb-c-Myc mice; *P* = .002 (2 tailed unpaired Student’s *t*-test). (B) Graph showing the % of WT, ΔS6, Alb-c-Myc and ΔS6:c-Myc mice at ≥ 6 months of age with % L/BWs in normal range (3.5–6%) or larger than normal (> 6%). Note that nearly 70% of ΔS6:c-Myc mice have %L/BWs of >10% indicative of moderate to extreme hepatomegaly. The numbers at the top of each bar denote the % of mice of each genotype with livers within the indicated size ranges (data derived from %/L/BW values in A). (C) Kaplan-Meier curve of tumor latency (% tumor-free mice) in WT, ΔS6, Alb-c-Myc and ΔS6:c-Myc mice. Age at which 50% of mice of develop at least 1 tumor: ΔS6, 374 days; Alb-c-Myc, 373 days; ΔS6:Alb-c-Myc; 334 days. ΔS6:c-Myc mice show a modest, but statistically significant decrease in tumor latency relative to ΔS6 (*P* = .022) or Alb-c-Myc (*P* = .0003) mice (Log-rank (Mantel-Cox) test).
(TIF)

S14 Fig Hepatoblast-specific expression of p53^QS^ does not stunt neonatal growth or induce liver hypoplasia and results in mild hepatic dysfunction that is not sufficient to trigger regeneration. (A) Graph of body weights in WT and p53^QS^ male mice from post-natal day 8 (P8) to ~19 weeks showing that, in contrast to loss of Rps6, hepatoblast-specific expression of p53^QS^ does not stunt growth. *P* values range from .06 to.79; 2-tailed unpaired Student’s *t*-test. (B) Graph of %L/BW values in WT and p53^QS^ male mice from P8 to ~18 weeks showing that p53^QS^ does not induce neonatal hepatic hypoplasia. p53^QS^ livers do however demonstrate a trend towards hepatomegaly as mice age, the extent to which varies between mice. *P* values range from .08 to .71, except for P41-47 where *P =* .02 *; 2-tailed unpaired Student’s *t*-test. (C) Photomicrographs of H&E stained livers from neonatal and young adult WT, p53^QS^ and ΔS6 mice. At P7 and P15, p53^QS^ livers remain relatively normal and fail to demonstrate any of the early pathophysiological signs of hepatic dysfunction seen in age-matched ΔS6 livers such as feathery degeneration (fd) of hepatocytes (c) or biliary infarcts (*) (f). Evidence of biliary dysfunction, however, becomes apparent in p53^QS^ livers as mice reach adulthood (bile infarcts, *) (h), but unlike loss of Rps6 (i), it is not sufficient to trigger nodular regenerative growth (n) or a dr. Original magnifications; all x 112.5; scale bars, 50μ.
(TIF)

S15 Fig Partial resolution of liver dysfunction in p53^QS^ mice in the absence of an obvious regenerative response. Liver function tests (LFTs) performed on plasma isolated from ≥ 3 WT and p53^QS^ mice at P28-35 and P49-60. While markers of hepatocellular (ALT and AST) and biliary (Alk-Phos and T-Bil) dysfunction are all elevated in p53^QS^ mice at 4–5 weeks, all show significant improvement by ~7–9 weeks of age despite the absence of an obvious regenerative response. *P* values were calculated using a 2-tailed unpaired Student’s *t*-test.
(TIF)

S16 Fig Hepatoblast-specific expression of p53^QS^ inhibits bile duct development. (A) SOX9 IHC of livers from WT and p53^QS^ mice at P8 and P15. p53^QS^ livers have fewer SOX9-positive cells and fail to form recognizable patent bile ducts (arrows). While at least 1 bile duct is visible adjacent to the portal vein in WT liver at P8 and P15, only ductal plate remnants remain in p53^QS^ livers by P15 (arrowheads). Original magnifications; a,b x 125; scale bars, 50μ; c,d x 250; scale bars, 25μ; d,e x 78; scale bars, 50μ). AEC chromagen, red; no counterstain. (B) Graph showing quantitation of the number of SOX9-positive cells/portal vein (PV) in WT and p53^QS^ livers at P8 and P15. Mean +/- SEM. p53^QS^ livers have ~ 50% of the normal number of SOX9-positive cells at P8 (22.5 vs 11.4; *P* < .0001) and at P15 (13.4 vs 7.5 *P* < .0001); 2 tailed unpaired Student’s *t*-test). (C) Graph showing quantitation of the number of fully formed bile ducts/PV in WT and p53^QS^ livers at P8 and P15. In contrast to WT livers which have an average of 1–2 fully formed bile ducts/PV at P8 and P15, p53^QS^ livers have <0.5 (*P* < .0001); 2 tailed unpaired Student’s *t* test. Bars represent mean +/- SEM. (D) SOX9 IHC of 3 month old age-matched WT (a) and p53^QS^ livers (b-d) showing the abnormal expansion of SOX9-positive cells around portal veins that are extending out into the parenchyma and attempting to form bile ducts. Arrows indicate possible bile ducts in p53^QS^ livers although they are small and barely patent. Hepatocyte heterogeneity is also evident with some hepatocytes having grossly enlarged nuclei (arrowheads). Original magnifications, all x 125; scale bars 50μ (inset in (a), x 250; scale bar, 25μ). AEC chromagen, red; hematoxylin counterstain, blue.
(TIF)

S17 Fig Liver failure in p53^QS^ mice is due to the loss of p53^QS^-expressing hepatocytes with livers becoming repopulated with immature liver cells that fail to express the p53^QS^ mutant. Photomicrographs of H&E stained sections (a, b) and IHC (c-h) of a p53^QS^ liver from a 4 month old mouse showing repopulation of the liver with immature liver cells that do not express the p53^QS^ mutant. In (a), the dashed lines represent borders between the immature cells (small tightly packed cells with high nuclear:cytoplasmic ratio) and residual hepatocytes adjacent to an area of hepatocyte necrosis (*). (b) Higher magnification of the immature cells with a high nuclear to cytoplasmic ratio and “tiled” arrangement resembling E12-14 hepatoblasts. (c) Low power image of p53 IHC of a p53^QS^ liver that has lost most of its p53^QS^ expressing hepatocytes that is being repopulated with immature cells that do not express the p53^QS^ mutant (arrows indicate several clusters of residual hepatocytes that still express abundant p53^QS^ (dark staining nuclei)). (d) High power image of a p53^QS^ liver showing p53^QS^-expressing hepatocytes (right of the dashed line) juxtaposed with small crowded p53^QS^-naïve immature cells (left of the dashed line). Low (e and g) and high (f and h) power images of the same p53^QS^ liver shown in a-d stained with an antibody specific for MYC (e, f) or EPCAM (g, h). While virtually all of the immature cells show abundant nuclear expression of MYC (e, f), only a subset show membrane expression of EPCAM (g, h) suggesting that livers are being repopulated by immature hepatic cells that have most likely been stalled at various stages of differentiation. Original magnifications; a) x 62.5, b) x 250, c, e, g) x 32.5, d, f, h) x 375. Scale bars; a) 100μ, b) 50μ, c, e, g) 200μ, d, f, h) 25μ. AEC chromagen, red/brown; hematoxylin counterstain, blue.
(TIF)

S18 Fig Co-deletion of p53 fails to significantly improve and even exacerbates liver dysfunction in ΔS6 livers. Liver function tests (LFTs) performed on heparin-treated plasma isolated from 32–40 day old WT, ΔS6 and ΔS6:Δp53 mice (n = ≥5). Values represent mean +/- SEM. While markers of biliary dysfunction either improved slightly (Alk-Phos) or remained the same (T-Bil), deletion of p53 exacerbated hepatocellular dysfunction in ΔS6 livers as seen by hyper-elevation of ALT and AST. **** *P* < .0001; all other *P* values as stated; 2-tailed unpaired Student’s *t*-test.
(TIF)

S19 Fig Loss of p53 does not restore bile duct development in *Rps6*-deficient livers. (A) IHC of livers from ~5 week old WT (a), ΔS6 (b) and ΔS6Δp53 (c and d) mice with a SOX9-specific antibody. Normal bile ducts in WT liver (a) are indicated (arrow heads), while an active ductular reaction (dr) composed of SOX9-positive cells attempting to form bile ducts is evident in regenerating ΔS6 livers (b). In ΔS6Δp53 livers, abundant SOX9-positive cells in the vicinity of portal veins radiate out into the parenchyma, but do not appear to be attempting to form bile ducts or ductules (c and d). AEC Chromagen (red/orange); no counterstain. Original magnifications, all x 112.5. All scale bars, 50μ. (B) H & E stained liver sections from a 4 month old (a and b) or 5 month old (c and d) ΔS6Δp53 mouse showing re-population of the parenchyma with immature hepatoblast-like cells. Original magnifications, x 62.5 (a and c); x 250 (b and d). Scale bars, 100μ (a and c); 50μ (b and d).
(TIF)

S20 Fig Hepatobiliary disease induced by Rps6 insufficiency is rescued by increasing the level of hepatic c-Myc but not by loss of p53 and is only partially mimicked by liver-specific expression of p53^QS^. (A) Schematic showing that Albumin-Cre-mediated deletion of Rps6 in the liver stabilizes p53, delays neonatal growth and results in hypoplastic liver development by inhibiting bile duct development and inducing hepatocyte death. Despite being severely runted and jaundiced as neonates, biliary function improves and livers regenerate allowing adult ΔS6 mice to reach normal size. With age, however, ΔS6 livers are predisposed to overgrow and develop tumors, which is accelerated by loss of the tumor-suppressor PTEN. While the neonatal growth defect and hepatobiliary disease are both significantly improved by bolstering the level of c-Myc in ΔS6 livers, loss of p53 fails to ameliorate and even exacerbates aspects of ΔS6-associated liver disease. (B) Schematic showing that Albumin-Cre-mediated expression of an MDM2-resistant p53 mutant (p53^QS^) mimics the bile duct defect but not the hepatocyte defect in ΔS6 livers resulting in a normal rate of post-natal growth and normal sized livers. In contrast to Rps6-deficient heptocytes which die, p53^QS^ expressing hepatocytes remain viable but enter a prolonged period of cell cycle arrest/senescence that later results in liver failure due to the eventual loss of p53^QS^-expressing hepatocytes.
(TIF)

S1 Table Differentially expressed genes in *Rps6*-deficient livers. (XLSM)

S2 Table % Liver/Body Weights of WT, ΔS6, ΔPTEN & ΔS6ΔPTEN mice. (XLSX)

S3 Table Incidence of prominent histopathology features of ΔS6ΔPTEN mice. (XLSX)

S4 Table Body weights of WT ΔS6 c-Myc & ΔS6:c-Myc mice. (XLSX)

S5 Table Oligonucleotide Primers for Genotyping. (XLSX)

S6 Table Antibodies reagents and IHC Conditions. (XLSX)

S7 Table Primers for sq-RT-PCR. (XLSX)

S8 Table Antibodies used for Immunoblotting. (XLSX)

S9 Table Raw Datasets for Figs 1–9 Graphs. (XLSX)

S10 Table Raw Datasets for S1–S20 Figs Graphs. (XLSX)
