# Supplementary figures and images for: Hepatic ribosomal protein S6 (Rps6) insufficiency results in failed bile duct development and loss of hepatocyte viability; a ribosomopathy-like phenotype that is partially p53-dependent
Source: PLoS Genet. 2023 Jan 19;19(1):e1010595. doi: 10.1371/journal.pgen.1010595 (PMC9888725; doi:10.1371/journal.pgen.1010595)

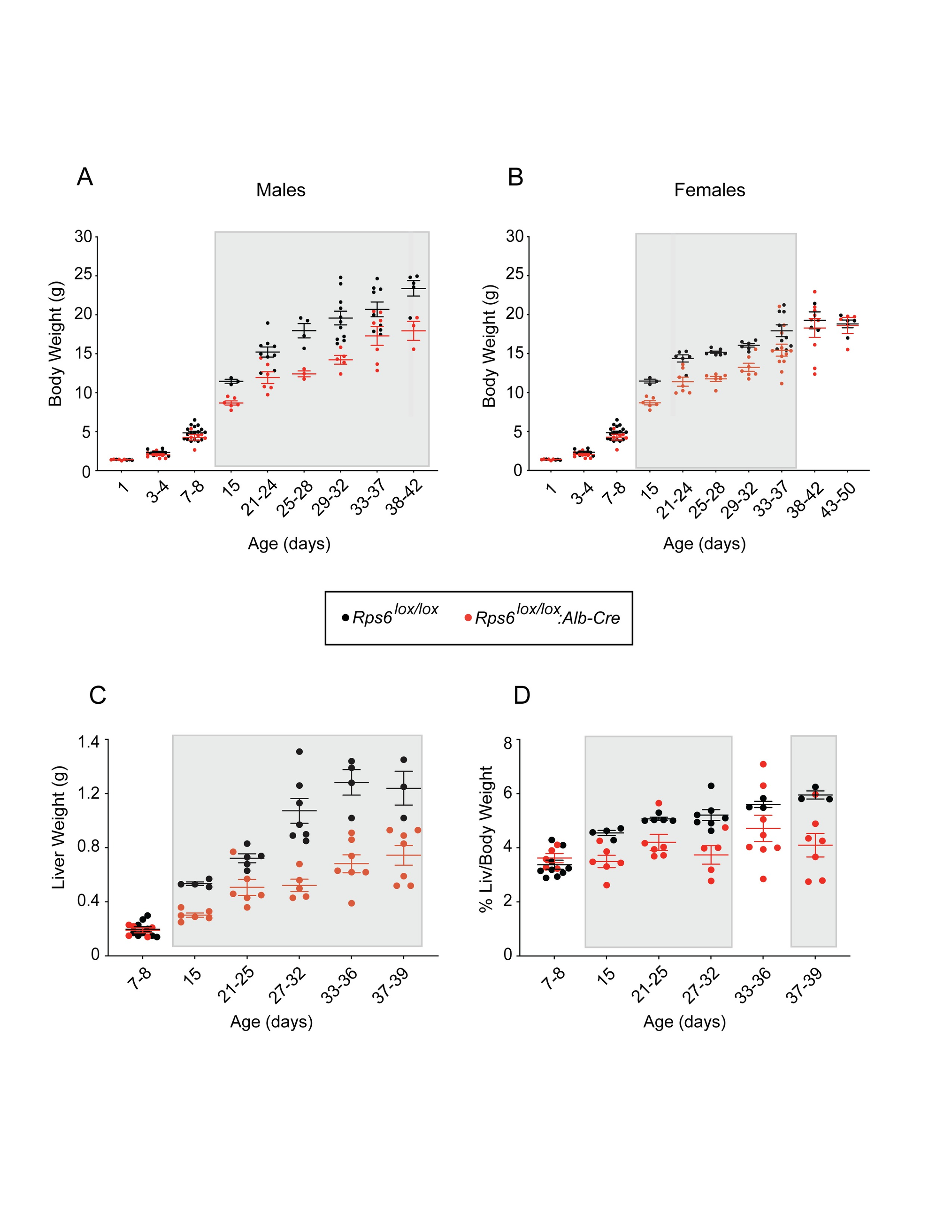

Supplement: S1 Fig — (A-D) Graphs of post-natal body weights, liver weights and %Liver/Body weights (%L/BWs) in WT and ΔS6 mice. In graphs (A and B) body weight values from post-natal day 1–15 (P1-P15) include male and female mice. From P21-24 onwards, graph A) represents values for males only while graph (B) represents values for females only. Gray boxes indicate ages at which body weight in ΔS6 mice differs significantly from WT. For males (A), P values range from .041 at P33-37 to .004 at P25-28. For females (B), P values range from .036 at P33-37 to < .0001 at P25-28. At P15, P = .0003; 2-tailed unpaired Student’s t test. (C and D) Graphs of liver weight (C) and %L/BW (D) in male WT and ΔS6 mice from P7-8 to P39. Gray boxes indicate ages at which liver weight and %L/BW values in ΔS6 mice differ significantly from WT. For liver weights (C), P values range from .016 at P21-25 to < .0001 at P15. For %L/BWs (D), P values range from .029 at P21-25 to .0028 at P27-32; 2- tailed unpaired Student’s t-test. (TIF) [file pgen.1010595.s001.tif]

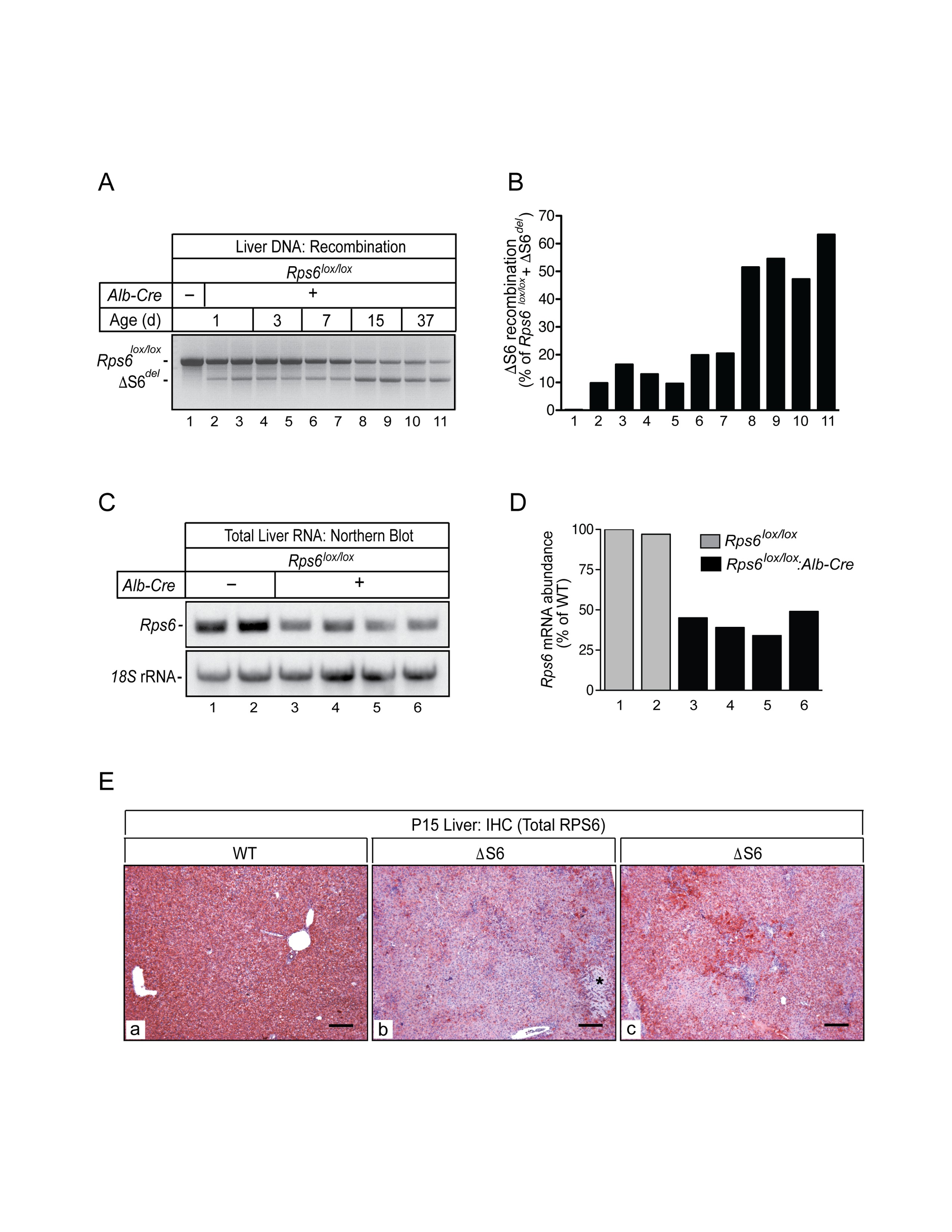

Supplement: S2 Fig — (A) Ethidium stained agarose gel showing PCR analysis of recombination of the ΔS6del allele (lower band) in control (WT) (Rps6lox/lox) (lane 1) and ΔS6 (S6lox/lox:Alb-Cre) livers (lanes 2–11) at different ages from P1 to P37. Recombination increases to a maximal level of ~50–60% by P37. (B) Graph showing quantitation of the gel shown in A) representing the % of the recombined ΔS6del allele (lower band) relative to the total signal in each lane (sum of the recombined (lower) ΔS6del band plus the non-recombined S6lox allele (upper band)). (C) Northern blotting of 12 μg of total liver RNA from WT (lanes 1 and 2) and ΔS6 livers (lanes 3–6) with a p32-radiolabeled Rps6-specific probe. After stripping, the blot was incubated with a p32-radiolabeled 18S rRNA probe. (D) Graph showing quantitation of the Northern blot shown in (C) demonstrating that Rps6 mRNA levels are decreased by 50–60% in ΔS6 livers relative to WT. (E) IHC of a P15 WT liver (a) and 2 individual ΔS6 livers (b and c) with an antibody that recognizes total RPS6 protein. RPS6 is expressed across the lobule in WT liver at P15 (a), but becomes more restricted to periportal zone 1 as the liver matures. Residual RPS6 protein is visible in the ΔS6 livers indicating that Alb-Cre-mediated deletion is incomplete and regional across individual livers and varies between mice. A bile infarct (*) is visible in the ΔS6 liver in (b). Original magnifications, all x 62.5. Scale bars; 100μ. AEC chromagen (red), hematoxylin counterstain (blue). (TIF) [file pgen.1010595.s002.tif]

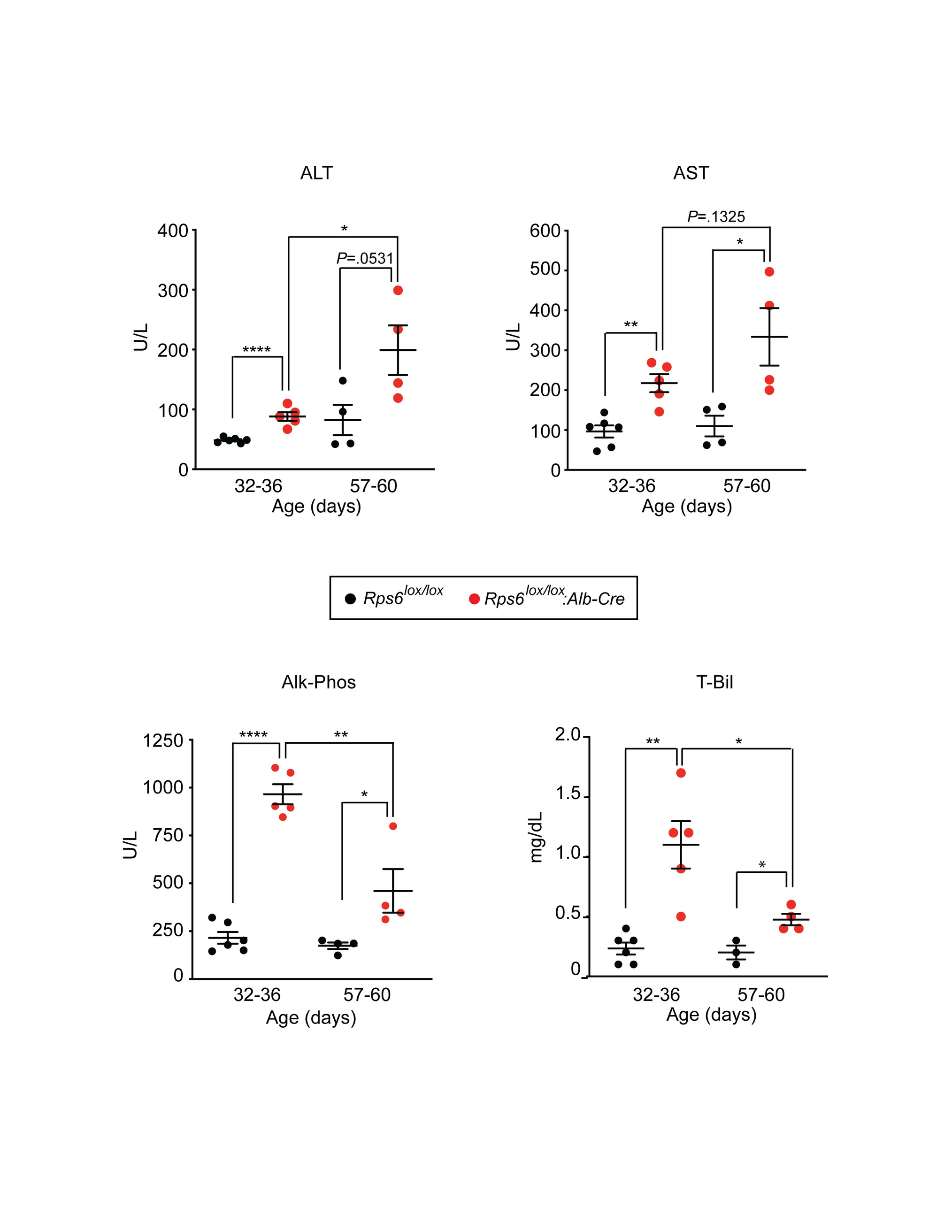

Supplement: S3 Fig — Liver function tests (LFTs) performed on heparin-treated plasma isolated from ≥ 4 control (Rps6lox/lox) and 4 ΔS6 (Rps6lox/lox:Alb-Cre) mice at P32-36 and P57-60. While markers of hepatocellular (ALT and AST) and biliary (Alk-Phos and T-Bil) dysfunction are all markedly elevated in ΔS6 mice at P32-P36, hepatocellular dysfunction persists, while biliary function improves as mice age (ALT, alanine aminotransferase; AST, aspartate aminotransferase; Alk-Phos, alkaline phosphatase; T-Bil, total bilirubin). ****P < .0001, ***P.<0005, **P < .005, *P < .05. (2-tailed unpaired Student’s t-test). (TIF) [file pgen.1010595.s003.tif]

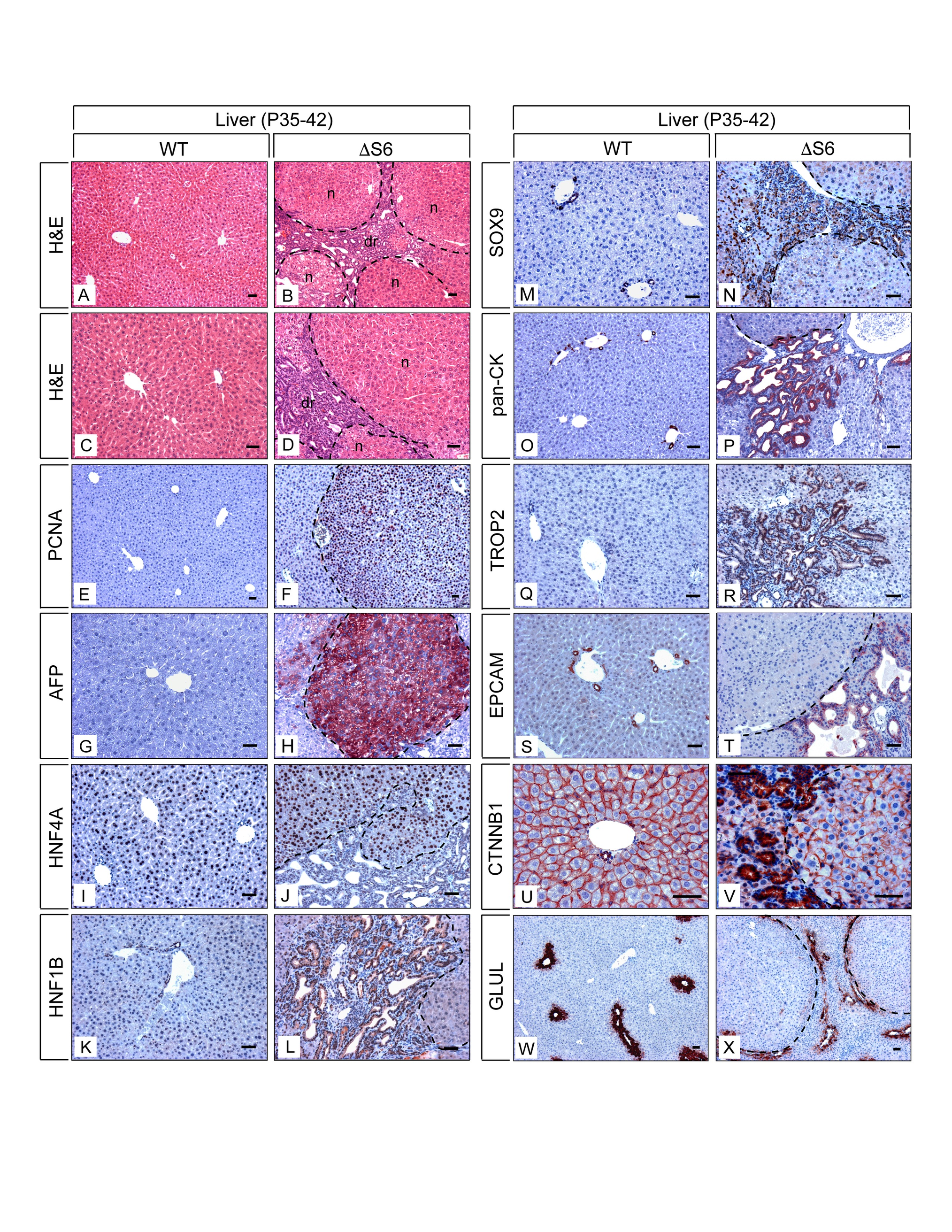

Supplement: S4 Fig — (A-D) Photomicrographs of H & E stained WT and ΔS6 livers between P35-P42 at low (A, B) and high (C, D) magnification showing regenerative nodules (n), (bounded by dotted lines) and the ductular reaction (dr) in ΔS6 livers. E-X) Photomicrographs of P35-P42 WT and ΔS6 livers after performing IHC for PCNA (e, f) and variety of markers known to be expressed in a cell-type or location-dependent manner within the liver. IHC profiling confirms that nodules are composed of highly proliferative AFP+, HNF4B+ immature hepatocytes (F, H), in contrast to cells within the dr, which are HNF1B+, SOX9+, pan-CK+, TROP2+ and EPCAM+ (L, N, P, R, T) consistent with an oval cell/HPC identity. Retention of β-catenin/CTNNB1 at the membrane of nodular hepatocytes (V) and the absence of staining of the hepatic β-catenin target GLUL (glutamine synthetase) in nodules (X) suggests that wnt signaling is not driving nodular growth in ΔS6 livers. AEC Chromagen (red), hematoxylin counterstain (blue). Original magnifications: A, B (x 75); C, D, O, P (x 112); E, F, W, X (x 62.5); G-N and Q-T (x 125); U, V (x 250). Scale bar; 50μ. (TIF) [file pgen.1010595.s004.tif]

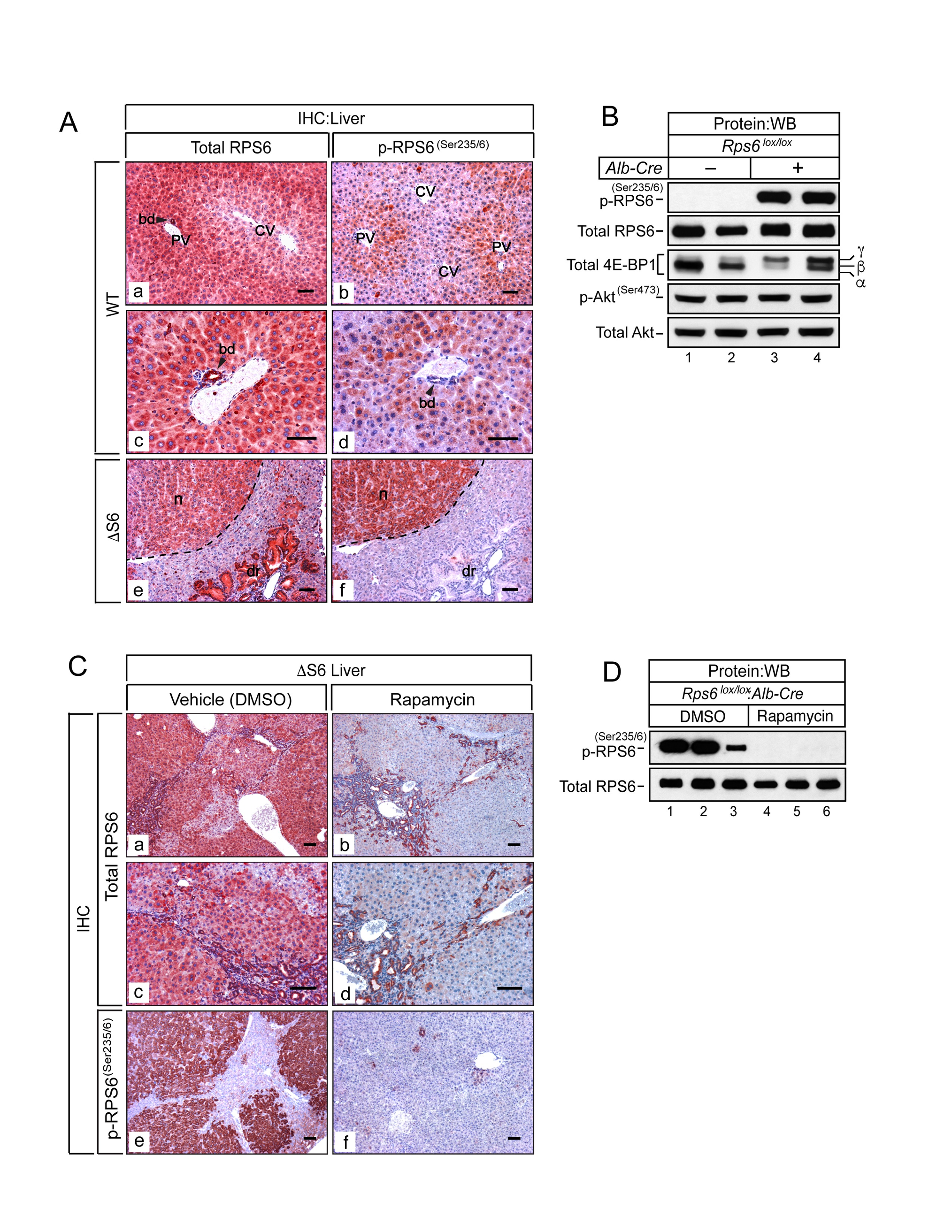

Supplement: S5 Fig — (A) IHC of WT (a-d) and ΔS6 liver (e, f) with an antibody that recognizes total RPS6 irrespective of its phosphorylation status (a, c, e) and one that recognizes RPS6 only when phosphorylated on the mTOR-dependent Ser235/236 sites (b, d, f). In WT liver, although RPS6 is highly expressed in bile ducts (bd, arrowhead) and throughout the parenchyma in a decreasing periportal-pericentral gradient (a, c), phosphorylated-RPS6(Ser235/6) is largely restricted to periportal hepatocytes and is absent from bile ducts (b, d). In ΔS6 liver, immature hepatocytes in nodules (n) and dr cells both express abundant RPS6 (e), yet it is only phosphorylated in nodular hepatocytes (f). Original magnifications: a, b, e, and f (x 112); c and d (x 225). Scales bars, 50μ. (B) Western blot of proteins isolated from WT (lanes 1 and 2) and ΔS6 livers (lanes 3 and 4) showing that mTOR signaling to 4E-BP1 and RPS6 is hyper-activated in ΔS6 livers, while Akt signaling is not. The absence of a visible p-RPS6Ser235/6 signal in WT livers reflects a level of Ser235/6 phosphorylation in regenerating ΔS6 livers (lanes 3 and 4) that is much higher than in WT liver (lanes 1 and 2) necessitating a short exposure that is not sufficient to visualize the p-RPS6 signal in WT liver. Phosphorylation of the mTOR target 4E-BP1 is indicated by an increase in the abundance of the higher molecular weight (γ) form of the protein in ΔS6 livers. (C and D). IHC (C) and Western Blotting (D) of livers with the total (a-d) and phospho-RPS6(Ser235/6) antibodies (e, f) in vehicle (DMSO) (a, c, e) and rapamycin-treated (b, d, f) ΔS6 mice showing that RPS6 phosphorylation in nodules of ΔS6 livers is mTOR-dependent. The residual signal in cells of the dr in rapamycin-treated livers reflects the presence of RPS6 protein that is not phosphorylated on Ser235/6 (b, d). AEC chromagen (red), hematoxylin counterstain (blue). Original magnifications: a, b, e and f (x 62.5); c and d (x 125). PV, portal vein; CV, central vein. Scale bars [file pgen.1010595.s005.tif]

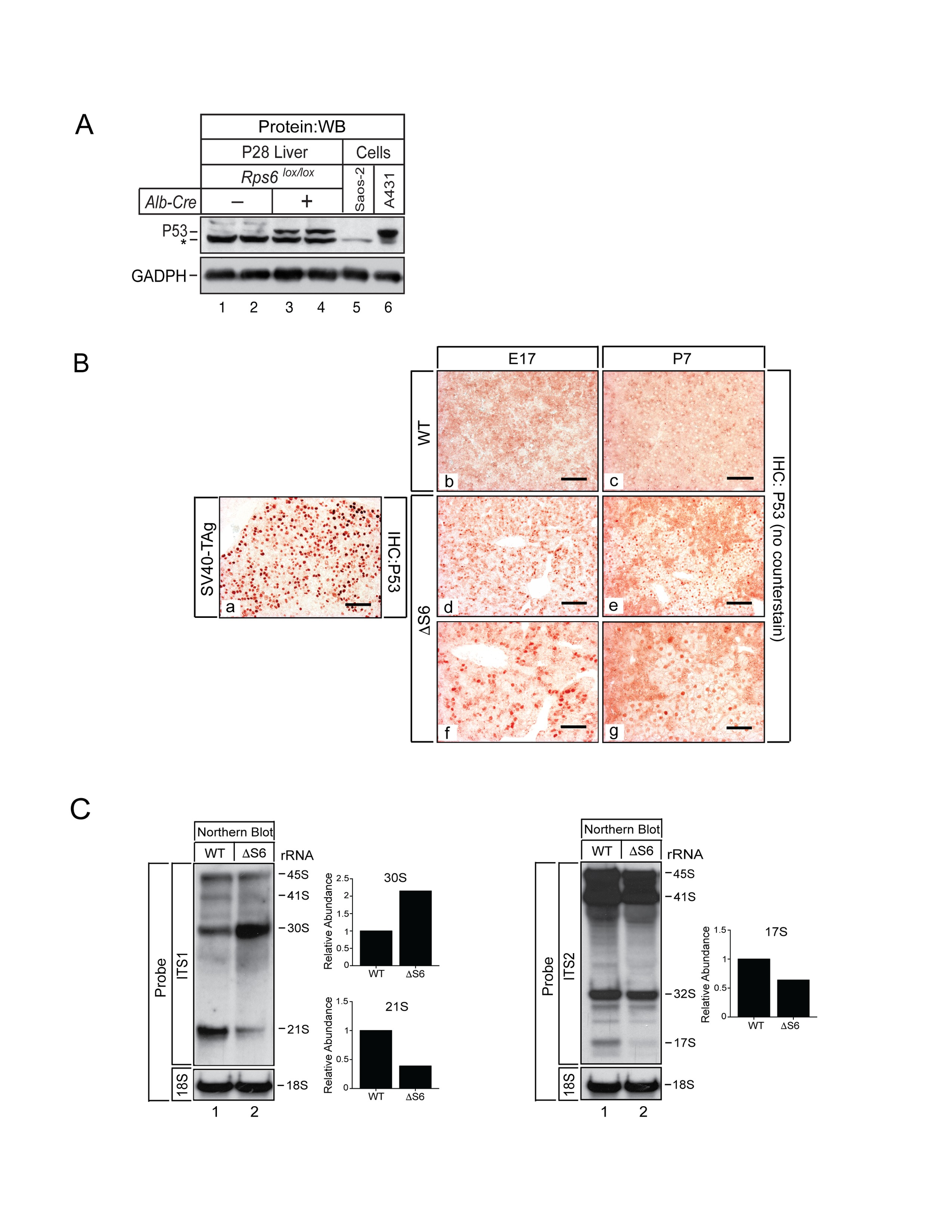

Supplement: S6 Fig — (A) Immunoblot of total protein lysates prepared from 2 WT (lanes 1 and 2) and 2 ΔS6 livers (lanes 3 and 4), p53-null Saos 2 cells (lane 5) and A431 cells that express high levels of mutant p53 (lane 6) with a p53-specific antibody and a GAPDH-specific antibody for load control. Abundant p53 protein is visible in ΔS6 livers (upper band, lanes 3 and 4) and A431 cells (lane 6). A faster migrating non-specific band (*) is present in all samples. (B) Photomicrographs of IHC of liver sections from an adult SV40 T-antigen (SV40-TAg) transgenic mouse (a) and from WT (b, c) and ΔS6 livers (d-g) at E17 and P7 incubated with a p53-specific antibody. p53 is stabilized in the nuclei of hepatocytes expressing SV40-TAg (a) and in a subset of hepatoblasts in ΔS6 livers in response to depletion of Rps6 (d-g). AEC chromogen (orange); no counterstain. Original magnifications; a, d, e (x 125; scale bars, 50μ); b, c, f, g (x 250; scale bars, 25μ). (C) Left: Northern blot of liver RNA from WT and ΔS6 mice hybridized to a radiolabeled ITS1 probe homologous to nucleotides 5977–6006 of the mouse 45S pre-ribosomal RNA (left) showing that Rps6 deficiency causes an rRNA processing defect that results in the accumulation of 30S rRNA and a decrease in the amount of 21S rRNA, both of which are precursors of the mature 18S rRNA. Right: Northern blot analysis of the same RNAs hybridized to a radiolabeled ITS2 probe homologous to nucleotides 7026–7065 of the mouse 45S pre-ribosomal RNA showing decreased abundance of 17S rRNA. Graphs were generated using Image J to estimate fold-changes in the abundance of rRNA species in ΔS6 livers relative to WT (assigned an arbitrary value of 1). (TIF) [file pgen.1010595.s006.tif]

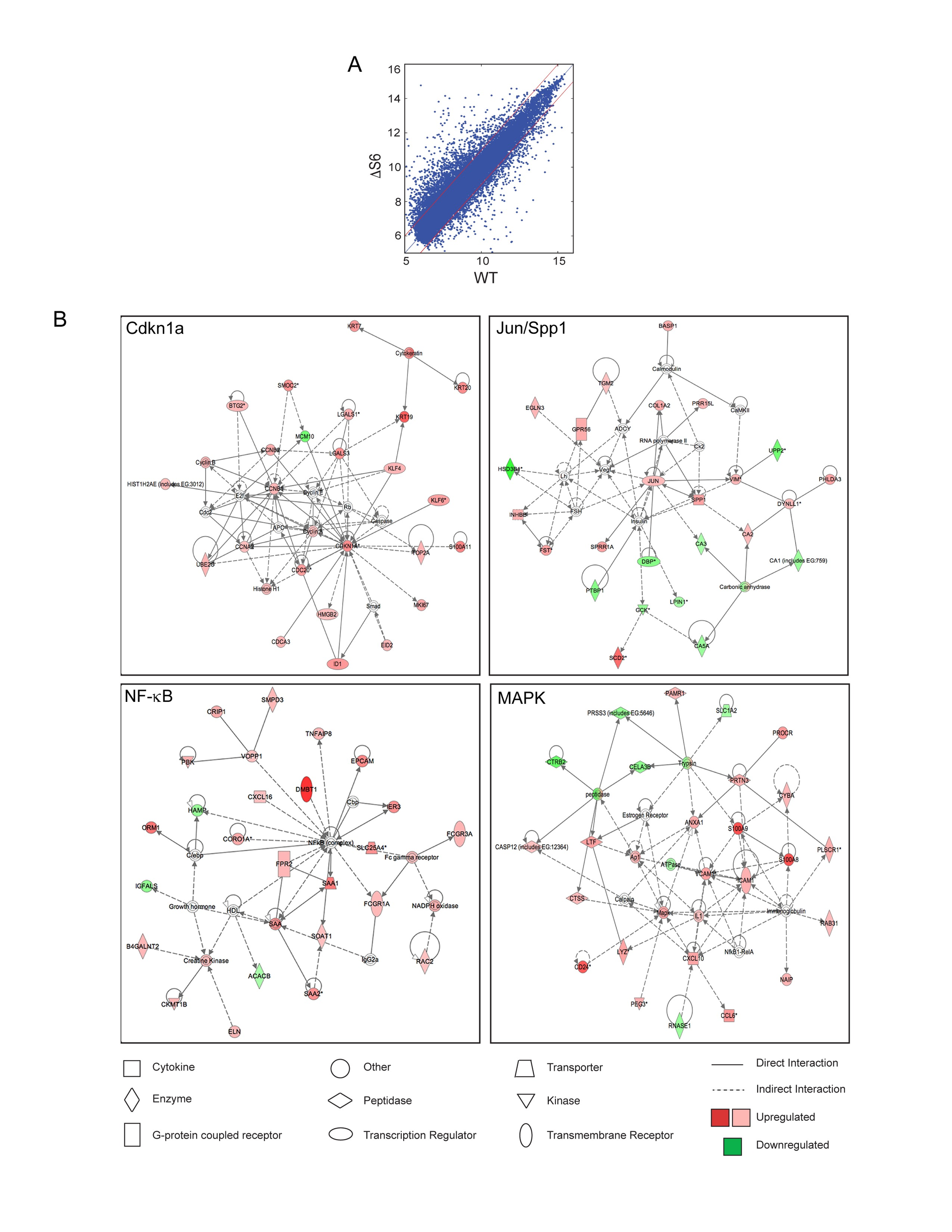

Supplement: S7 Fig — (A) Scatter plot of the microarray data. (B) Ingenuity pathway analysis (IPA) of the microarray data showing that loss of Rps6 activates hepatic gene expression programs associated with cell cycle arrest/senescence (cdkn1a, MAPK), regeneration (jun/spp1) and inflammation/activation of innate immunity (NF-κB). (TIF) [file pgen.1010595.s007.tif]

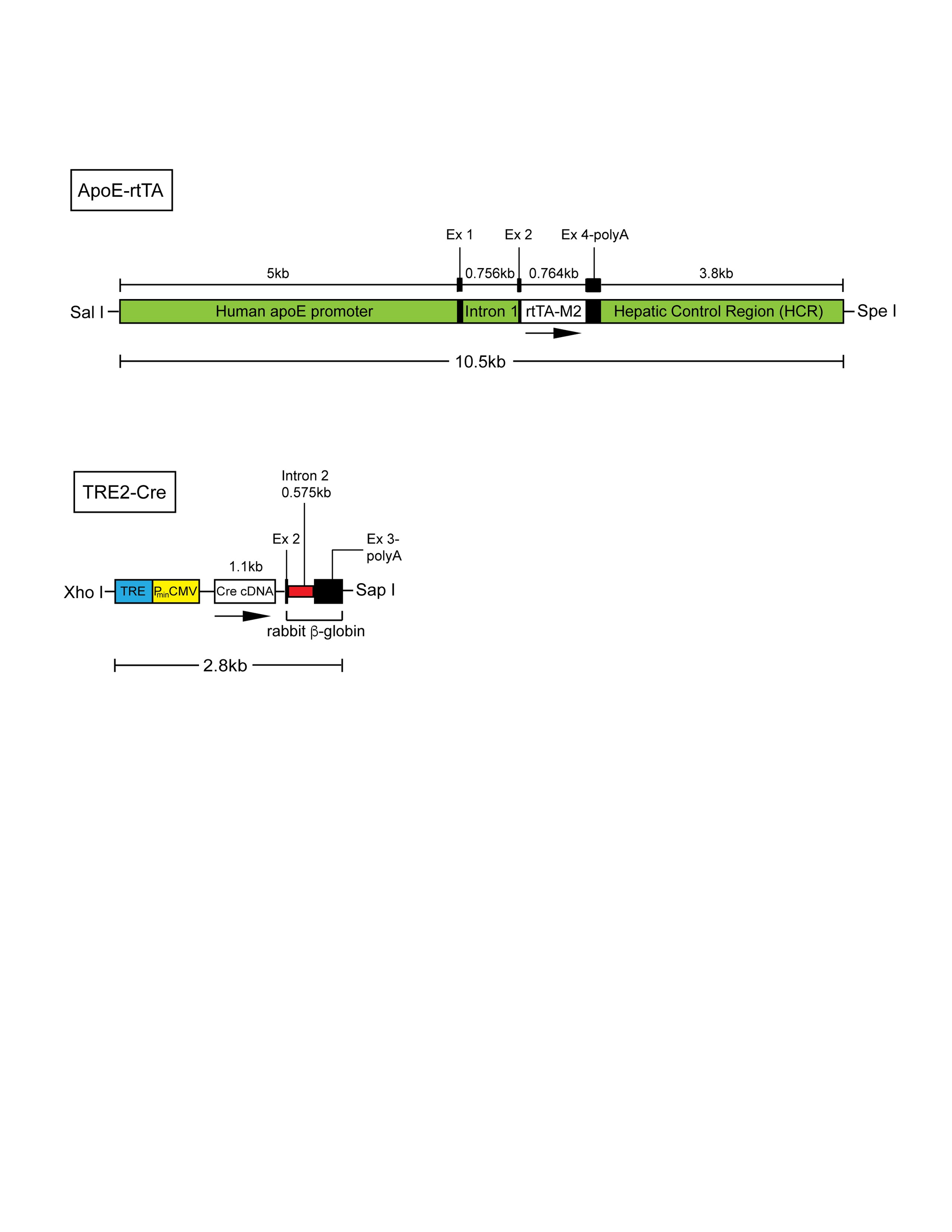

Supplement: S8 Fig — A 764bp fragment encoding the reverse tetracycline transactivator rtTA-M2 cDNA was subcloned into the Mun I-Cla I sites of plasmid pLIV11 containing the promoter, intron 1 and hepatic control region (HCR) of the human ApoE gene [50,168]. The resulting 10.5kb ApoE-rtTA transgene was excised from unwanted vector sequences by digestion with Sal I and Spe I. The TRE-Cre transgene was generated by subcloning a 1.1kb fragment encoding the Cre recombinase into the BamHI-Xba 1 sites of plasmid TRE2 containing the reverse tetracycline response element and minimal CMV promoter. The 2.8kb transgene was excised from the plasmid by digestion with Xho I and Sap I. Both transgenes were co-injected into one-cell embryos to generate ApoE-rtTA-TRE2-Cre bigenic mice in which liver-specific expression of Cre is induced in hepatocytes following doxycycline administration. (TIF) [file pgen.1010595.s008.tif]

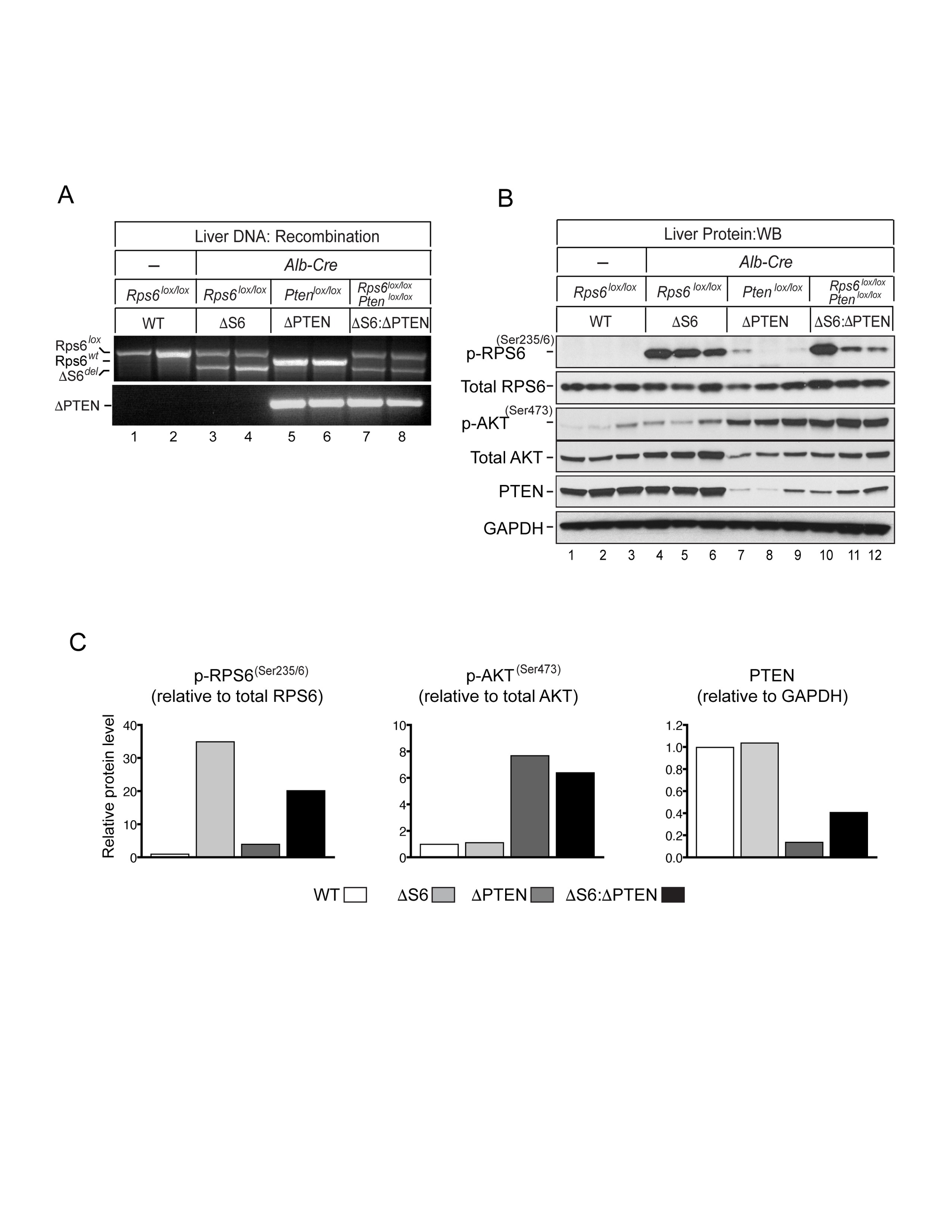

Supplement: S9 Fig — (A) Ethidium stained agarose gel showing that the efficiency of recombination of either the ΔS6del or ΔPTEN alleles is unaffected by the presence of the other in livers of mice doubly deficient for Rps6 and PTEN. (B) Western blots of proteins prepared from livers of 6–11 month old WT (lanes 1–3), ΔS6 (lanes 4–6), ΔPTEN (lanes 7–9) and ΔS6ΔPTEN (lanes 10–12) mice with antibodies specific for total and phospho-specific forms of RPS6 and AKT, PTEN and GAPDH (for load control). While RPS6, but not AKT is hyperphosphorylated in ΔS6 livers and AKT, but not RPS6, is hyperphosphorylated in ΔPTEN livers, both are hyperphosphorylated in ΔS6ΔPTEN livers indicating co-activation of both mTOR and PI3K. (C) Graphs showing Image J quantitation of phospho-RPS6(Ser235/6), phospho-AKT(Ser473) and PTEN protein levels from western blots shown in (B). (TIF) [file pgen.1010595.s009.tif]

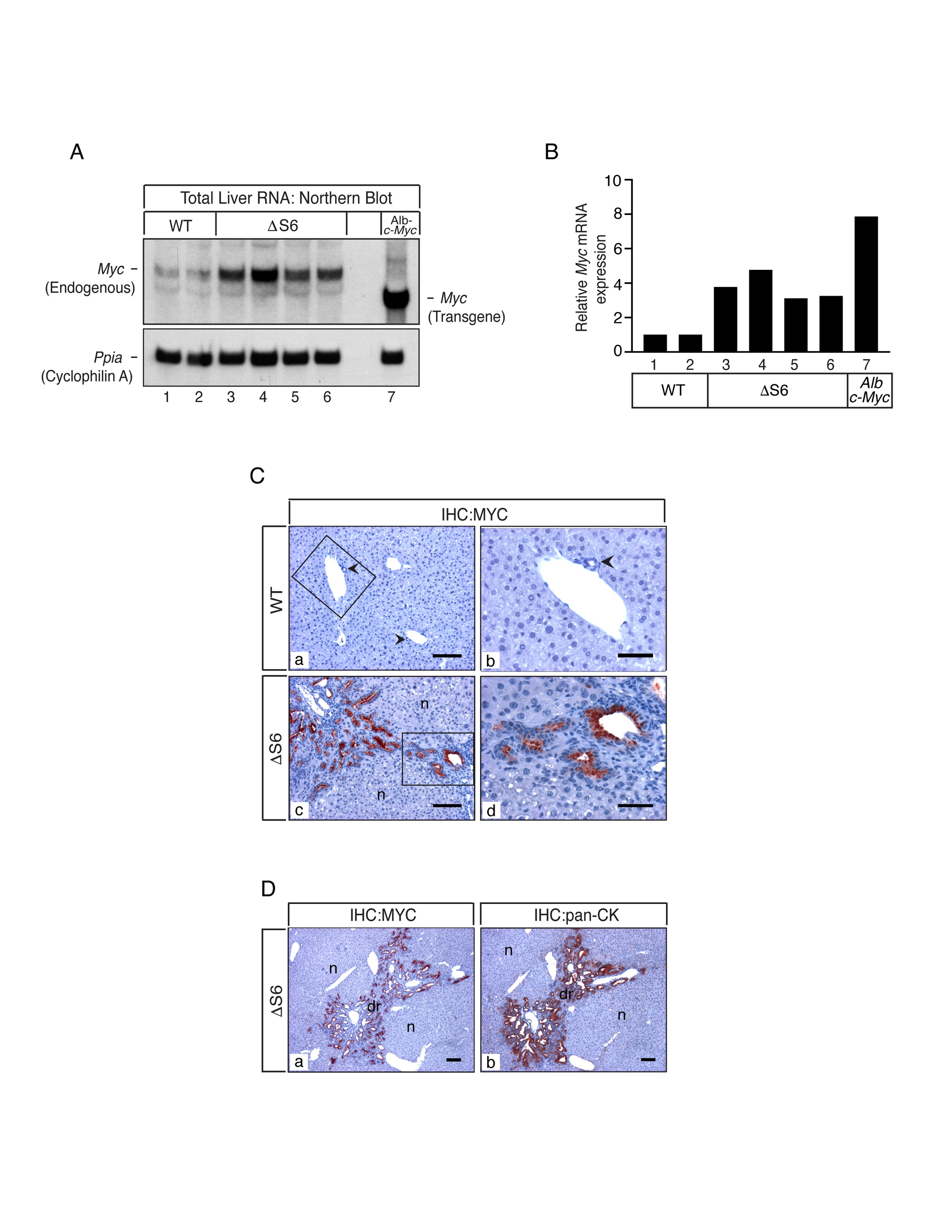

Supplement: S10 Fig — (A) Northern blot of total liver RNA (12μg/lane) from WT (lanes 1 and 2) and ΔS6 mice (lanes 3–6) and an Albumin-c-Myc transgenic mouse. The blot was first incubated with a p32-radiolabeled c-Myc-specific cDNA probe, followed by stripping and re-probing with an p32-radiolabeled cyclophilin A-specific cDNA probe. Note that the size of the endogenous Myc transcript differs from that transcribed from the Albumin-c-Myc transgene. (B) Graph of quantitation of relative Myc mRNA levels in Northern Blot shown in A). With Myc expression in WT liver set at an arbitrary value of 1, Myc mRNA is elevated ~4-5-fold in ΔS6 livers, slightly less than that expressed in livers of Albumin-c-Myc transgenic mice (~7-8-fold increase). (C) IHC with a c-Myc-specific antibody showing that MYC protein is undetectable in normal hepatocytes and biliary cells (arrowheads) in WT liver (a, b), but is abundantly expressed in HPCs within the ductular reaction (dr), but not regenerating nodules (n) of ΔS6 livers (c, d). Original magnifications; a, c x125; scale bars 50μ; b, d x312; scale bars, 25μ. (D) IHC of ΔS6 livers showing that MYC and pan-cytokeratin (pan-CK) are specifically expressed in and co-localize to HPCs within the dr. Original magnifications; a, b x 62.5; scale bars, 50μ. For all IHC, AEC Chromagen (red) with hematoxylin counterstain (blue). (TIF) [file pgen.1010595.s010.tif]

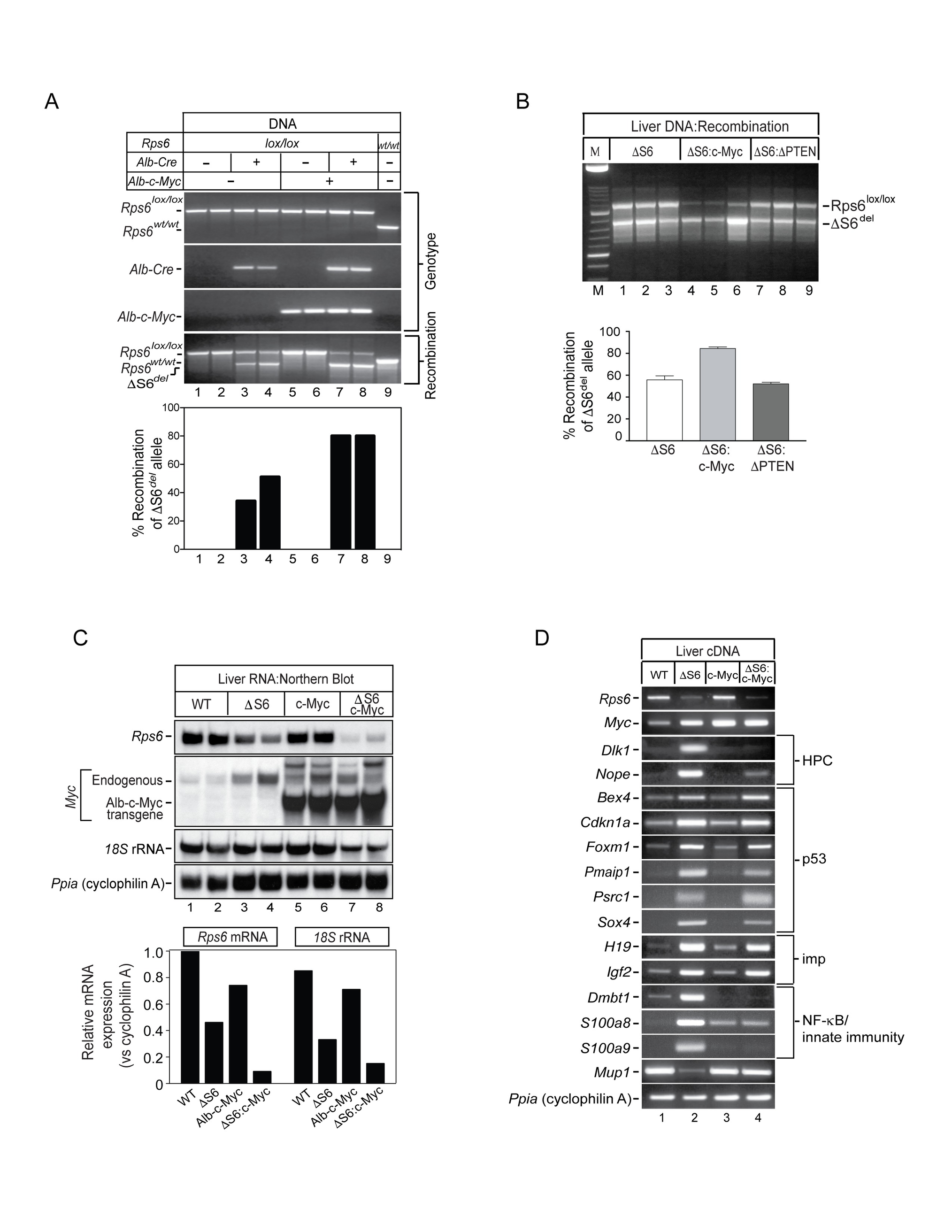

Supplement: S11 Fig — (A) Ethidium-stained agarose gels showing PCR genotyping of mice (top 3 panels) and recombination of the ΔS6del allele (lower panel) in livers of WT, ΔS6, Alb-c-Myc and ΔS6:c-Myc mice. By preserving hepatocyte viability, c-Myc overexpression inadvertently increases recombination of the ΔS6del allele from ~50% in ΔS6 mice (lanes 3 and 4) to ~80% in ΔS6:c-Myc mice (lanes 7 and 8). (B) Ethidium-stained agarose gel and graph of quantitation showing that the efficiency of recombination in ΔS6 livers (lanes 1–3) is augmented by c-Myc overexpression (ΔS6:Myc livers, lanes 4–6) but not by loss of PTEN (ΔS6:ΔPTEN livers, lanes 7–10). (C) Northern Blot of 12μg of total RNA isolated from WT, ΔS6, Alb-c-Myc and ΔS6:c-Myc livers with p32-radiolabeled probes specific for Rps6 (top panel), c-Myc (second panel), 18S rRNA (third panel) and Ppia/cyclophilin A (bottom panel). Note that Rps6 mRNA levels are lower in ΔS6:Myc livers (lanes 7 and 8) than in ΔS6 livers (lanes 3 and 4) reflecting the higher number of Rps6-negative hepatocytes in ΔS6:c-Myc livers as a consequence of c-Myc preserving hepatocyte viability. Note that the lower level of Rps6 in ΔS6:c-Myc livers has also become limiting for 18S rRNA production. Quantitation of relative mRNA levels for Rps6 and 18S rRNA are shown in the graph below the blot. (D) Semi-quantitative (sq)-PCR of cDNA prepared from WT, ΔS6, Alb-c-Myc and ΔS6:c-Myc liver. c-Myc normalizes the expression of a specific subset of mRNAs related to activation of NF-κB and innate immunity and HPC-activation induced in response to loss of S6, but not classical p53 targets or imprinted genes. Results reflect sq-PCR conducted on cDNA synthesized from total RNA isolated from the livers of ≥3 individual mice of each genotype. (HPC, hepatic progenitor cell associated genes; imp, imprinted genes). (TIF) [file pgen.1010595.s011.tif]

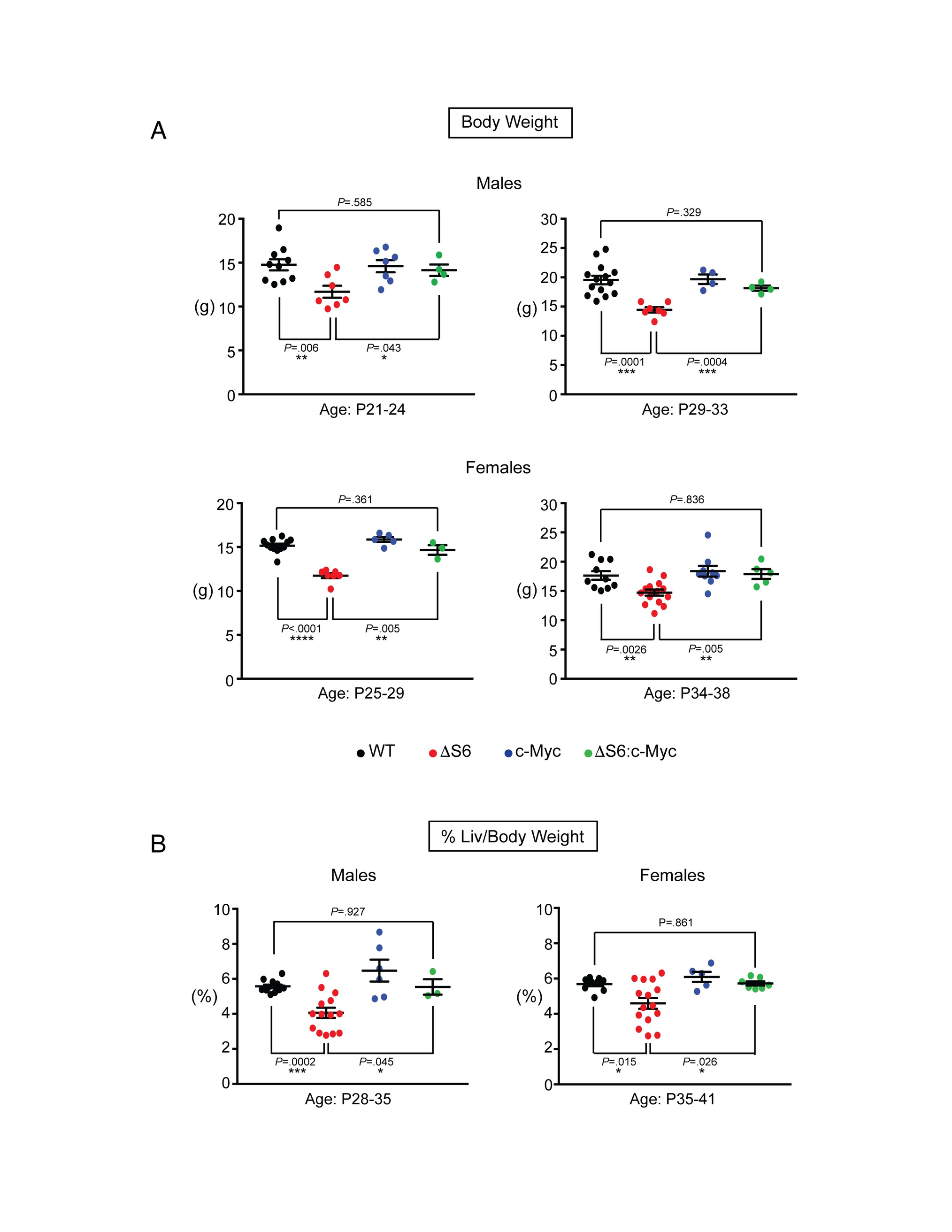

Supplement: S12 Fig — (A) Graphs of body weights of male (top 2 graphs) and female (bottom 2 graphs) WT, ΔS6, Alb-c-Myc (c-Myc) and ΔS6:c-Myc mice at different ages showing that body weights of ΔS6:c-Myc mice are indistinguishable from WT mice between ~3 and 5.5 weeks of age, the age at which ΔS6 mice show the greatest degree of growth retardation. (B) Graphs of % Liver/body weights in males and females showing that overexpression of c-Myc in ΔS6 livers also rescues the liver hypoplasia associated with Rps6-insufficiency. Significance was calculated using the 2-tailed unpaired Student’s t-test. Additional body weight analysis for one extra time point for both males and females is shown in accompanying S4 Table. (TIF) [file pgen.1010595.s012.tif]

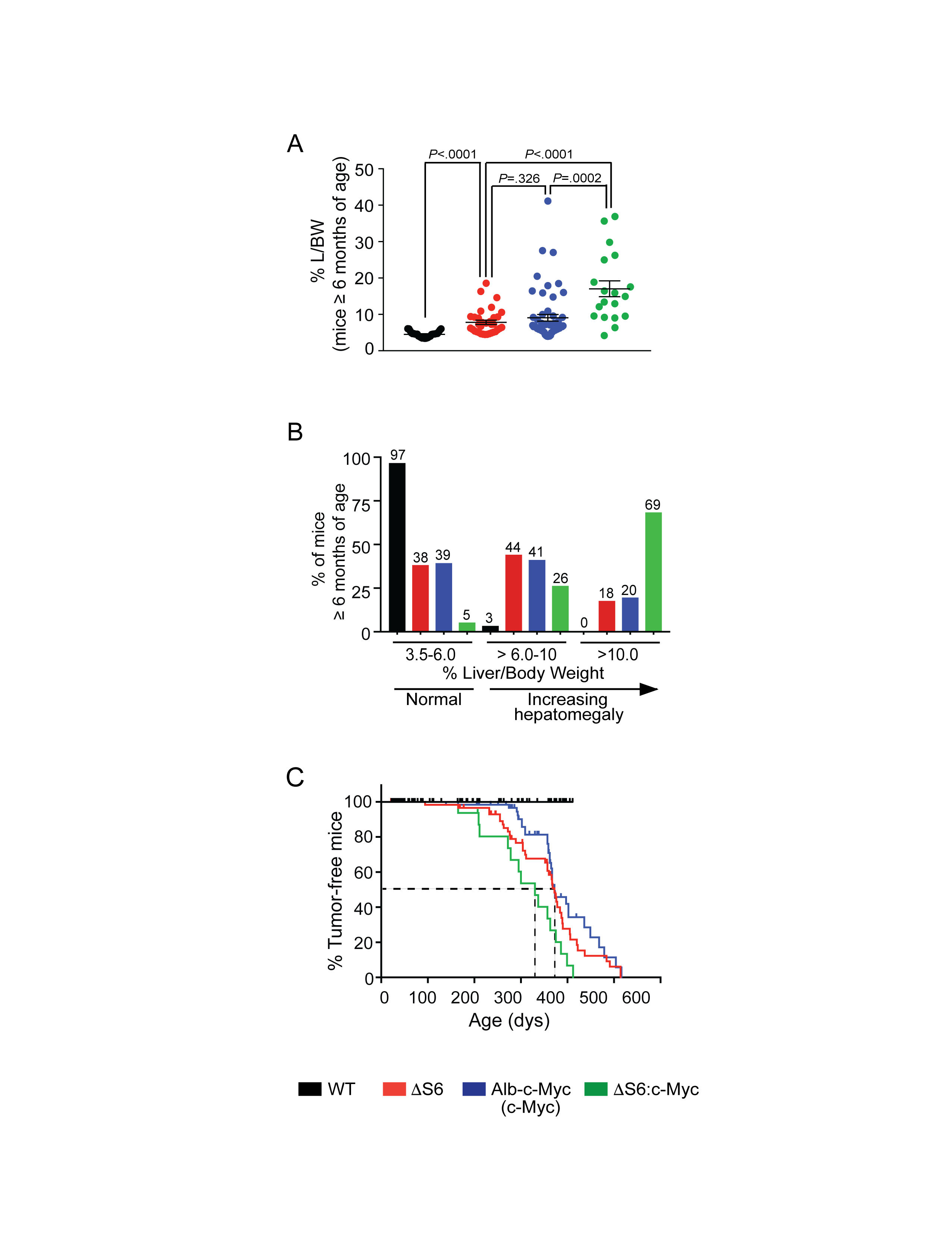

Supplement: S13 Fig — (A) Graph of %L/BWs of WT, ΔS6, Alb-c-Myc and ΔS6:c-Myc mice at ≥ 6 months of age showing that livers of ΔS6, Alb-c-Myc and ΔS6:c-Myc mice are all predisposed to overgrow as they age. Mean %L/BWs (+/- SEM) (number of mice): WT (4.5 +/0.15) (n = 29); ΔS6 (7.8 +/- 0.59) (n = 34); Alb-c-Myc (9.1 (+/- 0.92) (n = 56); ΔS6:c-Myc (17.0 +/- 2.19) (n = 19). The statistical difference in %L/BWs between ΔS6:Myc and ΔS6 mice; P< .0001, and between ΔS6:c-Myc mice and Alb-c-Myc mice; P = .002 (2 tailed unpaired Student’s t-test). (B) Graph showing the % of WT, ΔS6, Alb-c-Myc and ΔS6:c-Myc mice at ≥ 6 months of age with % L/BWs in normal range (3.5–6%) or larger than normal (> 6%). Note that nearly 70% of ΔS6:c-Myc mice have %L/BWs of >10% indicative of moderate to extreme hepatomegaly. The numbers at the top of each bar denote the % of mice of each genotype with livers within the indicated size ranges (data derived from %/L/BW values in A). (C) Kaplan-Meier curve of tumor latency (% tumor-free mice) in WT, ΔS6, Alb-c-Myc and ΔS6:c-Myc mice. Age at which 50% of mice of develop at least 1 tumor: ΔS6, 374 days; Alb-c-Myc, 373 days; ΔS6:Alb-c-Myc; 334 days. ΔS6:c-Myc mice show a modest, but statistically significant decrease in tumor latency relative to ΔS6 (P = .022) or Alb-c-Myc (P = .0003) mice (Log-rank (Mantel-Cox) test). (TIF) [file pgen.1010595.s013.tif]

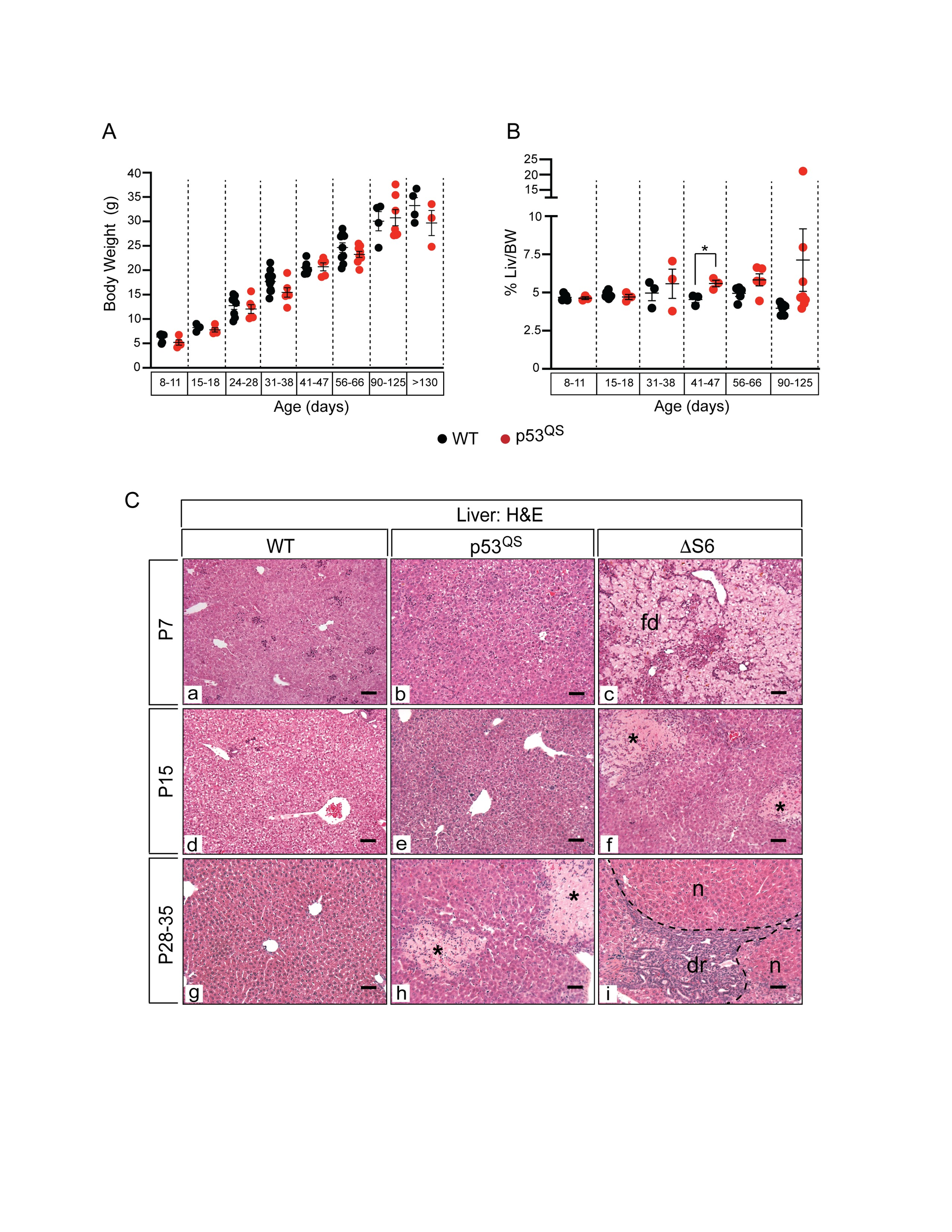

Supplement: S14 Fig — (A) Graph of body weights in WT and p53QS male mice from post-natal day 8 (P8) to ~19 weeks showing that, in contrast to loss of Rps6, hepatoblast-specific expression of p53QS does not stunt growth. P values range from .06 to.79; 2-tailed unpaired Student’s t-test. (B) Graph of %L/BW values in WT and p53QS male mice from P8 to ~18 weeks showing that p53QS does not induce neonatal hepatic hypoplasia. p53QS livers do however demonstrate a trend towards hepatomegaly as mice age, the extent to which varies between mice. P values range from .08 to .71, except for P41-47 where P = .02 *; 2-tailed unpaired Student’s t-test. (C) Photomicrographs of H&E stained livers from neonatal and young adult WT, p53QS and ΔS6 mice. At P7 and P15, p53QS livers remain relatively normal and fail to demonstrate any of the early pathophysiological signs of hepatic dysfunction seen in age-matched ΔS6 livers such as feathery degeneration (fd) of hepatocytes (c) or biliary infarcts (*) (f). Evidence of biliary dysfunction, however, becomes apparent in p53QS livers as mice reach adulthood (bile infarcts, *) (h), but unlike loss of Rps6 (i), it is not sufficient to trigger nodular regenerative growth (n) or a dr. Original magnifications; all x 112.5; scale bars, 50μ. (TIF) [file pgen.1010595.s014.tif]

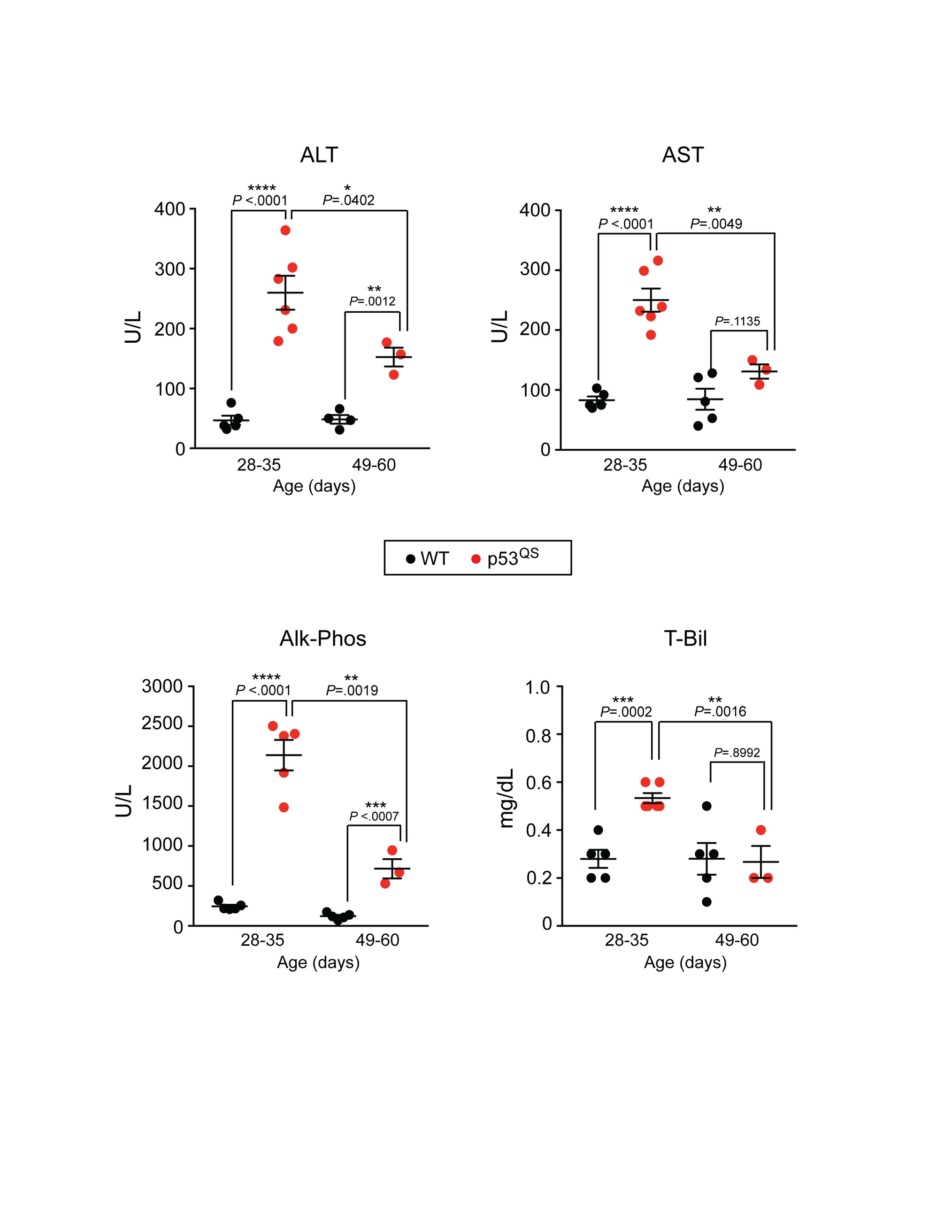

Supplement: S15 Fig — Liver function tests (LFTs) performed on plasma isolated from ≥ 3 WT and p53QS mice at P28-35 and P49-60. While markers of hepatocellular (ALT and AST) and biliary (Alk-Phos and T-Bil) dysfunction are all elevated in p53QS mice at 4–5 weeks, all show significant improvement by ~7–9 weeks of age despite the absence of an obvious regenerative response. P values were calculated using a 2-tailed unpaired Student’s t-test. (TIF) [file pgen.1010595.s015.tif]

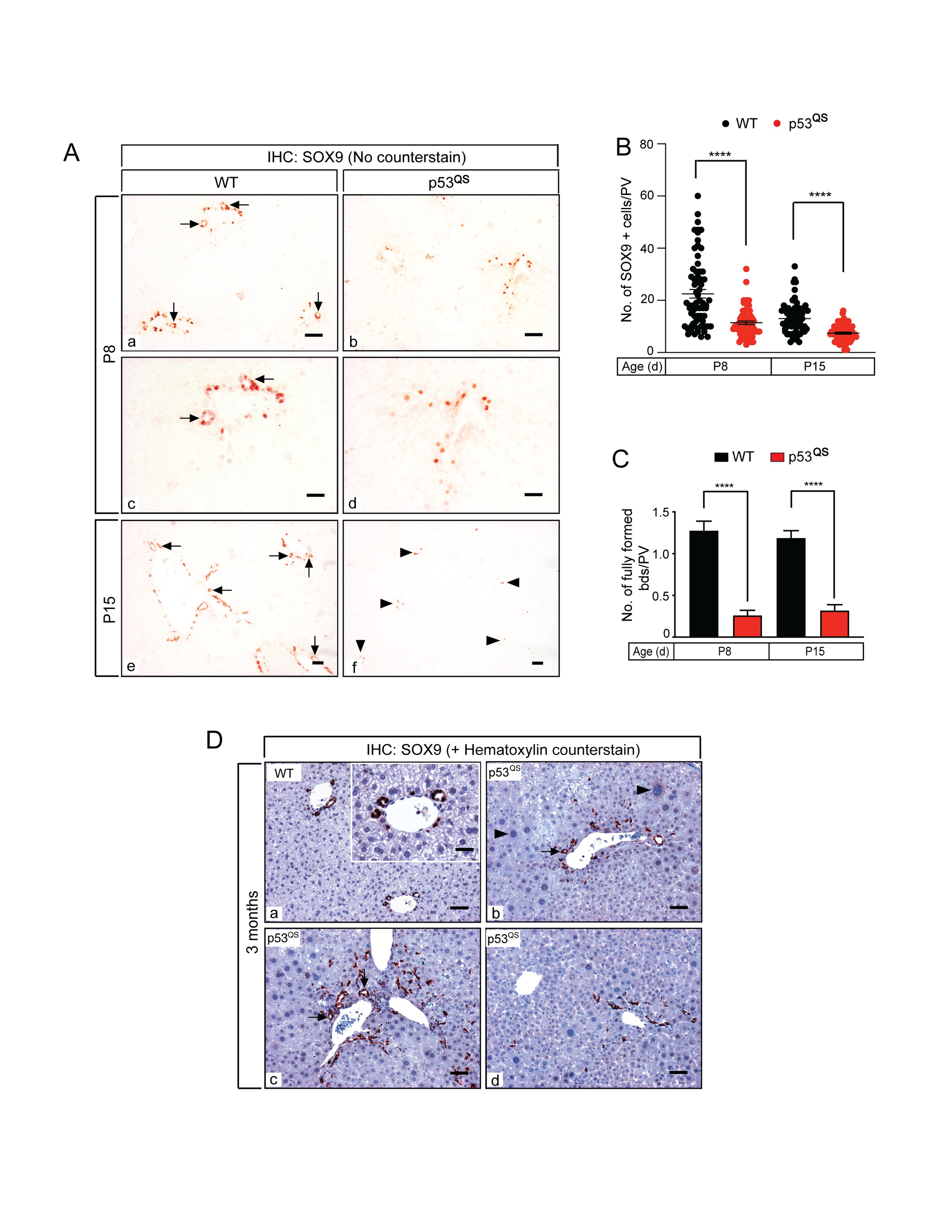

Supplement: S16 Fig — (A) SOX9 IHC of livers from WT and p53QS mice at P8 and P15. p53QS livers have fewer SOX9-positive cells and fail to form recognizable patent bile ducts (arrows). While at least 1 bile duct is visible adjacent to the portal vein in WT liver at P8 and P15, only ductal plate remnants remain in p53QS livers by P15 (arrowheads). Original magnifications; a,b x 125; scale bars, 50μ; c,d x 250; scale bars, 25μ; d,e x 78; scale bars, 50μ). AEC chromagen, red; no counterstain. (B) Graph showing quantitation of the number of SOX9-positive cells/portal vein (PV) in WT and p53QS livers at P8 and P15. Mean +/- SEM. p53QS livers have ~ 50% of the normal number of SOX9-positive cells at P8 (22.5 vs 11.4; P < .0001) and at P15 (13.4 vs 7.5 P < .0001); 2 tailed unpaired Student’s t-test). (C) Graph showing quantitation of the number of fully formed bile ducts/PV in WT and p53QS livers at P8 and P15. In contrast to WT livers which have an average of 1–2 fully formed bile ducts/PV at P8 and P15, p53QS livers have <0.5 (P < .0001); 2 tailed unpaired Student’s t test. Bars represent mean +/- SEM. (D) SOX9 IHC of 3 month old age-matched WT (a) and p53QS livers (b-d) showing the abnormal expansion of SOX9-positive cells around portal veins that are extending out into the parenchyma and attempting to form bile ducts. Arrows indicate possible bile ducts in p53QS livers although they are small and barely patent. Hepatocyte heterogeneity is also evident with some hepatocytes having grossly enlarged nuclei (arrowheads). Original magnifications, all x 125; scale bars 50μ (inset in (a), x 250; scale bar, 25μ). AEC chromagen, red; hematoxylin counterstain, blue. (TIF) [file pgen.1010595.s016.tif]

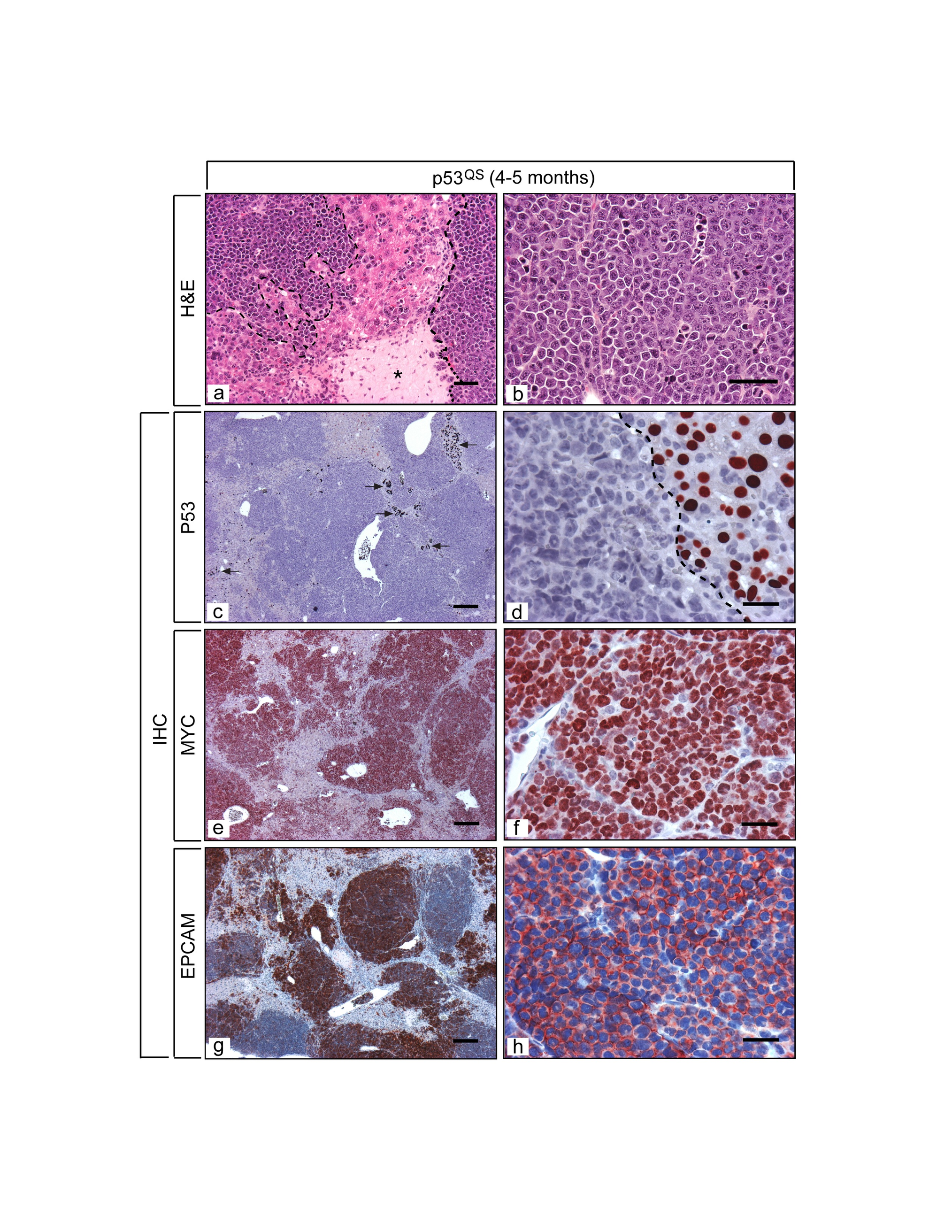

Supplement: S17 Fig — Photomicrographs of H&E stained sections (a, b) and IHC (c-h) of a p53QS liver from a 4 month old mouse showing repopulation of the liver with immature liver cells that do not express the p53QS mutant. In (a), the dashed lines represent borders between the immature cells (small tightly packed cells with high nuclear:cytoplasmic ratio) and residual hepatocytes adjacent to an area of hepatocyte necrosis (*). (b) Higher magnification of the immature cells with a high nuclear to cytoplasmic ratio and “tiled” arrangement resembling E12-14 hepatoblasts. (c) Low power image of p53 IHC of a p53QS liver that has lost most of its p53QS expressing hepatocytes that is being repopulated with immature cells that do not express the p53QS mutant (arrows indicate several clusters of residual hepatocytes that still express abundant p53QS (dark staining nuclei)). (d) High power image of a p53QS liver showing p53QS-expressing hepatocytes (right of the dashed line) juxtaposed with small crowded p53QS-naïve immature cells (left of the dashed line). Low (e and g) and high (f and h) power images of the same p53QS liver shown in a-d stained with an antibody specific for MYC (e, f) or EPCAM (g, h). While virtually all of the immature cells show abundant nuclear expression of MYC (e, f), only a subset show membrane expression of EPCAM (g, h) suggesting that livers are being repopulated by immature hepatic cells that have most likely been stalled at various stages of differentiation. Original magnifications; a) x 62.5, b) x 250, c, e, g) x 32.5, d, f, h) x 375. Scale bars; a) 100μ, b) 50μ, c, e, g) 200μ, d, f, h) 25μ. AEC chromagen, red/brown; hematoxylin counterstain, blue. (TIF) [file pgen.1010595.s017.tif]

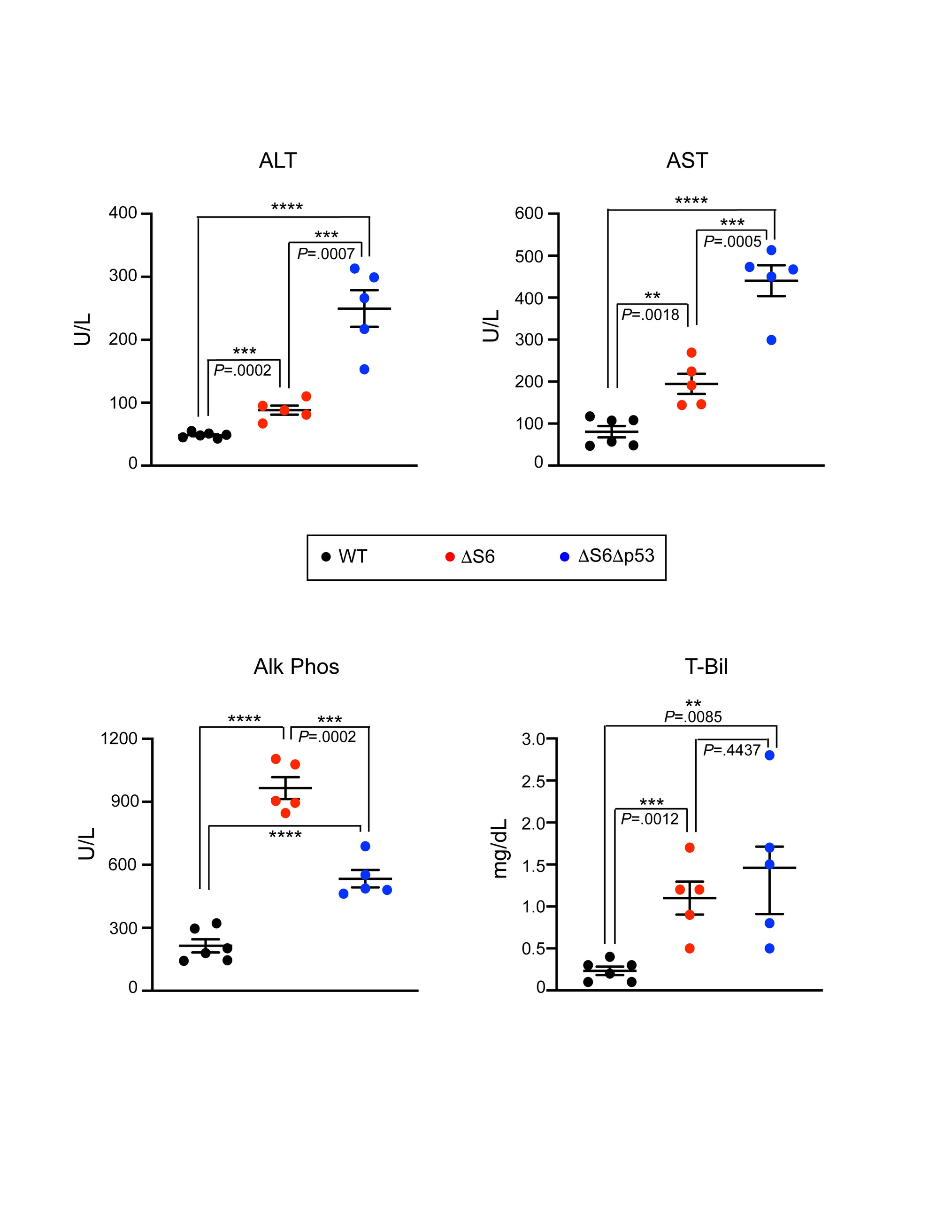

Supplement: S18 Fig — Liver function tests (LFTs) performed on heparin-treated plasma isolated from 32–40 day old WT, ΔS6 and ΔS6:Δp53 mice (n = ≥5). Values represent mean +/- SEM. While markers of biliary dysfunction either improved slightly (Alk-Phos) or remained the same (T-Bil), deletion of p53 exacerbated hepatocellular dysfunction in ΔS6 livers as seen by hyper-elevation of ALT and AST. **** P < .0001; all other P values as stated; 2-tailed unpaired Student’s t-test. (TIF) [file pgen.1010595.s018.tif]

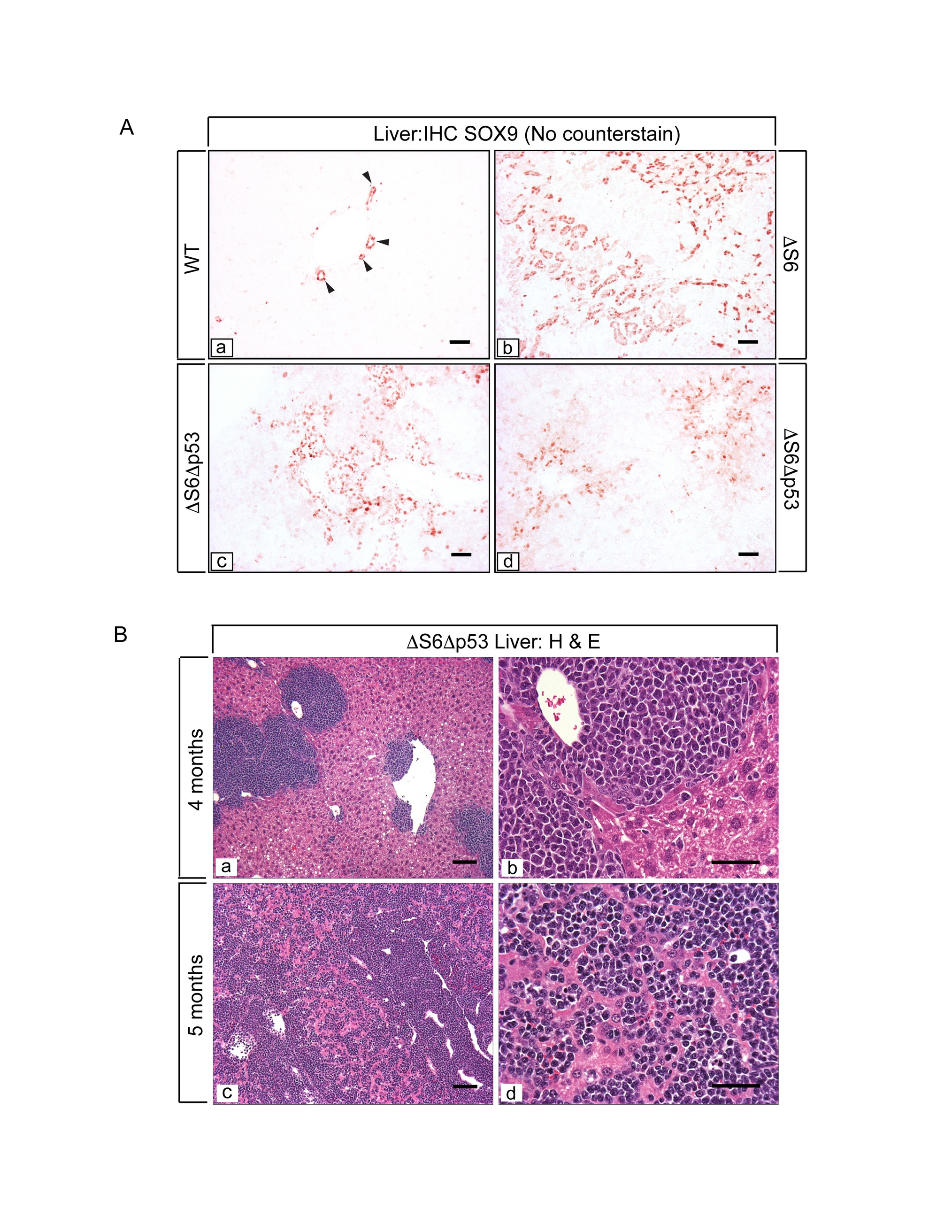

Supplement: S19 Fig — (A) IHC of livers from ~5 week old WT (a), ΔS6 (b) and ΔS6Δp53 (c and d) mice with a SOX9-specific antibody. Normal bile ducts in WT liver (a) are indicated (arrow heads), while an active ductular reaction (dr) composed of SOX9-positive cells attempting to form bile ducts is evident in regenerating ΔS6 livers (b). In ΔS6Δp53 livers, abundant SOX9-positive cells in the vicinity of portal veins radiate out into the parenchyma, but do not appear to be attempting to form bile ducts or ductules (c and d). AEC Chromagen (red/orange); no counterstain. Original magnifications, all x 112.5. All scale bars, 50μ. (B) H & E stained liver sections from a 4 month old (a and b) or 5 month old (c and d) ΔS6Δp53 mouse showing re-population of the parenchyma with immature hepatoblast-like cells. Original magnifications, x 62.5 (a and c); x 250 (b and d). Scale bars, 100μ (a and c); 50μ (b and d). (TIF) [file pgen.1010595.s019.tif]

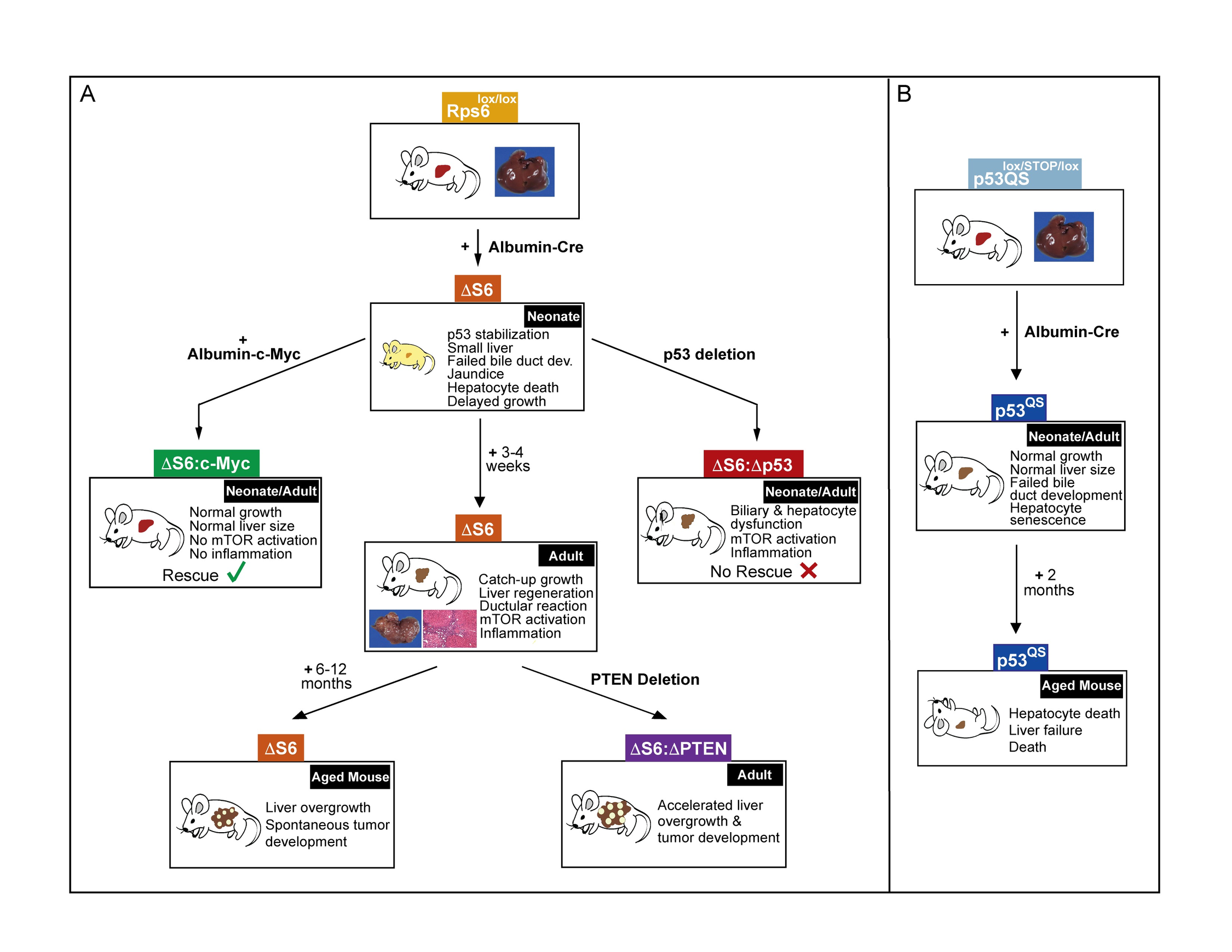

Supplement: S20 Fig — (A) Schematic showing that Albumin-Cre-mediated deletion of Rps6 in the liver stabilizes p53, delays neonatal growth and results in hypoplastic liver development by inhibiting bile duct development and inducing hepatocyte death. Despite being severely runted and jaundiced as neonates, biliary function improves and livers regenerate allowing adult ΔS6 mice to reach normal size. With age, however, ΔS6 livers are predisposed to overgrow and develop tumors, which is accelerated by loss of the tumor-suppressor PTEN. While the neonatal growth defect and hepatobiliary disease are both significantly improved by bolstering the level of c-Myc in ΔS6 livers, loss of p53 fails to ameliorate and even exacerbates aspects of ΔS6-associated liver disease. (B) Schematic showing that Albumin-Cre-mediated expression of an MDM2-resistant p53 mutant (p53QS) mimics the bile duct defect but not the hepatocyte defect in ΔS6 livers resulting in a normal rate of post-natal growth and normal sized livers. In contrast to Rps6-deficient heptocytes which die, p53QS expressing hepatocytes remain viable but enter a prolonged period of cell cycle arrest/senescence that later results in liver failure due to the eventual loss of p53QS-expressing hepatocytes. (TIF) [file pgen.1010595.s020.tif]
